# Dual XH–π Interaction of Hexafluoroisopropanol with Arenes

**DOI:** 10.3390/molecules26154558

**Published:** 2021-07-28

**Authors:** Le Lu, Ruimao Hua

**Affiliations:** Key Laboratory of Organic Optoelectronics & Molecular Engineering of Ministry of Education, Department of Chemistry, Tsinghua University, Beijing 100084, China; lul18@mails.tsinghua.edu.cn

**Keywords:** HFIP, hydrogen bond–π, non-classical hydrogen bond, orbital interactions, arenes

## Abstract

The dual XH (OH and CH) hydrogen-bond-donating property of 1,1,1,3,3,3-hexafluoroisopropanol (HFIP) and the strong dual XH–π interaction with arenes were firstly disclosed by theoretical studies. Here, the high accuracy post-Hartree–Fock methods, CCSD(T)/CBS, reveal the interaction energy of HFIP/benzene complex (−7.22 kcal/mol) and the contribution of the electronic correlation energy in the total interaction energy. Strong orbital interaction between HFIP and benzene was found by using the DFT method in this work to disclose the dual XH–π intermolecular orbital interaction of HFIP with benzene-forming bonding and antibonding orbitals resulting from the orbital symmetry of HFIP. The density of states and charge decomposition analyses were used to investigate the orbital interactions. Isopropanol (IP), an analogue of HFIP, and chloroform (CHCl_3_) were studied to compare them with the classical OH–π, and non-classical CH–π interactions. In addition, the influence of the aggregating effect of HFIP, and the numbers of substituted methyl groups in benzene rings were also studied. The interaction energies of HFIP with the selected 24 common organic compounds were calculated to understand the role of HFIP as solvent or additive in organic transformation in a more detailed manner. A single-crystal X-ray diffraction study of hexafluoroisopropyl benzoate further disclosed and confirmed that the CH of HFIP shows the non-classical hydrogen-bond-donating behavior.

## 1. Introduction

1,1,1,3,3,3-Hexafluoroisopropanol (HFIP) recently appeared as a popular solvent in the past few decades due to its special physical and chemical properties. HFIP is widely used to promote reactions such as C-H bond activation [1,2], Lewis acid-catalyzed reactions [3,4], electrophilic aromatic substitution (S*_E_*Ar) [5,6,7,8,9], synthetic polymers [10,11,12], proteomics [13], and electrochemical synthesis [14,15]. A comprehensive review on the application of HFIP has appeared recently [16]. The special proprieties result from its contribution to the acidity and aggregating effect, forming a micelle-like microstructure [17], observed by using the small-angle X-ray scatter method (SAXS) [18,19]. On the other hand, the CH–π hydrogen bond with range of *ca* 1–5 kcal/mol each unit interaction [20,21] has been regarded as an important interaction in biological macromolecules [22]. Therefore, the detailed interaction behavior between HFIP (OH and/or CH bonds) and hydrocarbons is expected to be interesting and important to understand the solvent effect in organic transformation and in biological studies (HFIP as solvent), which remains to be investigated.

Nakagawa suggested that dipole–quadrupole interaction plays an important role in the CH–π interaction between arenes [23]. HFIP has an O-H bond, which is about 10^8^-fold more acidic (pKa = 9.3) [24] than isopropanol (IP, pKa = 17.1) [25] in aqueous solution due to two strong electron-withdrawing groups of CF_3_; thus, HFIP is a strong hydrogen bond donor. In addition, the C-H bond in HFIP shows a non-classical hydrogen bond donor due to the carbon directly attached to two CF_3_ groups and one hydroxyl group with a strong inductive effect by electron-withdrawing groups, and the high electronegativity of oxygen atoms. Shahi and coworkers investigated the strong hydrogen bond between HFIP and water, and the rotational behavior of HFIP in vacuum via the microwave spectroscopic and theoretical method [26,27]. The charge–dipole interactions between HFIP and halides were also studied by Wang’s group using photoelectron spectroscopy and the computational method [28]. However, the relative studies on the non-classical hydrogen bond of HFIP and its interaction with organic compounds on the orbital interaction level have not been reported yet. Therefore, we report here our finding in the strong orbital interaction between HFIP and arenes by computational methods, to disclose a dual XH–π (O-H and C-H with π) interaction. In addition, in this work, the CH of HFIP showing the non-classical hydrogen-bond-donating manner has been confirmed by the experimental evidence. Moreover, for comparison, the interaction energies of HFIP with 24 selected common organic compounds have also been calculated to give a comprehensive understanding of the behavior of HFIP in intermolecular interaction.

## 2. Computational Methods

To investigate the interaction between HFIP and common organic compounds, including hydrogen bonds, electrostatic interaction, and weak intermolecular forces such as dispersion forces, and van der Waals forces, the choice of a reasonable calculation method is critical to properly calculate the interaction energy. M06-2X is a hybrid-meta general gradient approximation (HM-GGA) method [29] and has been suggested for use with a Pople basis set 6-311++G(2d,2p) in the prediction of the interaction energy of hydrogen bond–π interaction, which can give the closest results to the experimental values, and is comparable to the precision of CCSD(T)/CBS [30]. In this work, therefore, M06-2X/6-311++G(2d,2p) will be used for geometry optimization and the calculation of interaction energies of the XH–π complexes. All calculations were performed by using Gaussian 16 quantum software package [31]. The density of states (DOS), the partial density of states (PDOS), and the charge decomposition analysis (CDA) were calculated by using Multiwfn [32]. VMD [33] and CYLView [34] were used for the visualization of the results.

## 3. Results and Discussion

**Figure 1:**
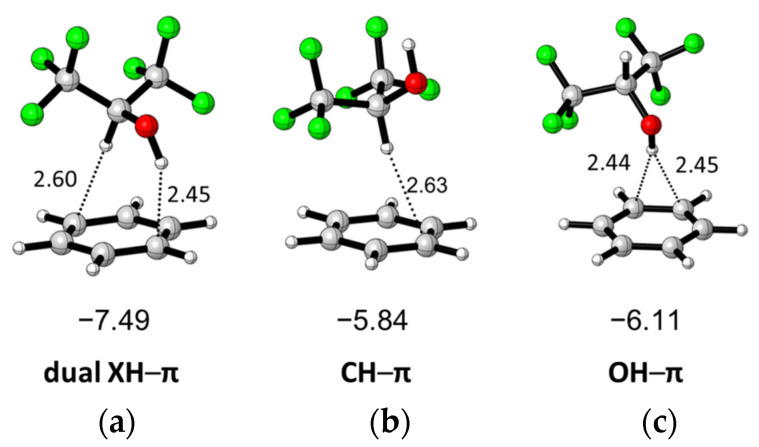
Three different optimized complexes of HFIP/PhH including (**a**) dual XH–π, (**b**) CH–π, and (**c**) OH–π interaction and their complexation energies (kcal/mol).

Three conformers of HFIP, including antiperplanar (AP), synclinal (SC), and synperiplanar (SP), were investigated by a computational method reported by Berkessel and coworkers, and it was found that the electronic energy of HFIP(SP) is the global minimum structure and HFIP(AP) is a local minimum, with 0.5 kcal/mol higher energy than HFIP(SP) in a PCM solvation model [35]. On the basis of their results, and three interaction complexes of HFIP (SP and AP) with benzene having XH–π interaction can be found as shown in Figure 1 after geometry optimization. The interaction energies of these complexes were calculated at M06-2X/6-311++G(2d,2p) level in vacuum with counterpoise correction to minimize the basis set superposition error (BSSE) [36,37]. The hfip(SP)/benzene complex shows the highest interaction energy, -7.49 kcal/mol, with dual XH–π interaction. HFIP(AP) interacts with benzene to form two different complexes containing CH–π or OH–π interactions, respectively. The interaction energy of HFIP(AP)/benzene in the CH–π complex is −5.84 kcal/mol, which is 0.64 kcal/mol higher than the experimental value of the CH–π complex of CHCl_3_/benzene (5.2 ± 0.2 kcal/mol) [38]. The interaction energy of HFIP(AP)/benzene in the OH–π complex is -6.11 kcal/mol, which is 0.27 kcal/mol higher than the CH–π complex.

Meanwhile, interaction energies between CHCl_3_/PhH and IP/PhH (IP: isopropanol, an analogue of HFIP) were also calculated at the same theory level to compare with the interaction energy of the HFIP(SP)/PhH complex. It was found that the interaction energy of CHCl_3_/PhH is −5.65 kcal/mol, which is 0.45 kcal/mol higher than the experimental value [38], and the interaction energy of IP/PhH is −4.53 kcal/mol. Therefore, the interaction energy of the HFIP(SP)/PhH complex is much higher than its analogue complex of IP/PhH.

The M06-2X/6-311++G(2d,2p)-level method was used in the geometry optimization of HFIP(SP)/PhH, CHCl_3_/PhH, and IP/PhH complexes, as concluded in Table 1. The electronic interaction energies of these complexes were also calculated with different methods including counterpoise corrections to confirm the use of the M06-2X/6-311++G(2d,2p) method to be the suitable choice.

Hartree–Fock (HF) is known to miss the electronic correlation energy term which is important to describe dispersion forces. Therefore, interaction energy calculation by using Hartree–Fock method only shows repulsion, the positive interaction energies. To account for the electronic correlation energy term, the post-Hartree–Fock method including MP2 (Møller–Plesset second-order perturbation theory), and CCSD(T) (coupled-cluster method with single and double substitutions and noniterative triple excitations) were used to calculate the interaction energy of hydrogen bond–π complexes mentioned above. In addition, the computational cost of CCSD(T)/CBS is notably large and is regarded as the gold standard in computational chemistry, but it is difficult to calculate directly. Thus, the energies of CCSD(T)/CBS were calculated as the sum of MP2/CBS and the estimated CCSD(T) correction term Δ(CCSD(T)-MP2).

The CCSD(T) correction terms were separately calculated with 6-31+G(d,p) and 6-311++G(d,p) basis sets to examine the effect by using small basis sets to the CCSD(T) correction term. The complete basis set (CBS) was achieved by the extrapolation method reported by Truhlar’s group [39,40,41]. The counterpoise method was used in the correction of BSSE in all calculations of interaction energies. The CCSD(T)/CBS using a 6-31+G(d,p) basis set in the correction term (Δ(CCSD(T)-MP2)) shows the interaction energy of HFIP/PhH (−7.21 kcal/mol), CHCl_3_/PhH (−6.11 kcal/mol), and IP/PhH (−4.43 kcal/mol) are slightly different than using the larger 6-311++G(d,p) basis set in the correction term in the interaction energy of HFIP/PhH (−7.22 kcal/mol), CHCl_3_/PhH (−5.97 kcal/mol), and IP/PhH (−4.36 kcal/mol). To compare with the experimental result of the binding energy of CHCl_3_/PhH (5.2 ± 0.2 kcal/mol), the result of CCSD(T)/CBS is already in chemical accuracy without the consideration of zero-point energy (ZPE) correction. As the compared method, M06-2X/6-311++G(2d,2p) shows a comparable value (−5.65 kcal/mol) in the prediction of the interaction energy in CHCl_3_/PhH to the CCSD(T)/CBS method, and performs well in the prediction of the dissociation energy the CHCl_3_/PhH complex (5.10 kcal/mol), including zero-point vibrational energy correction (ΔZPE).

On the basis of the M06-2X/6-311++G(2d,2p) method, the interaction energy of HFIP/PhH (−7.49 kcal/mol) is 1.84 kcal/mol larger than CHCl_3_/PhH, showing a dual XH–π interaction in HFIP/PhH. In order to understand the origin of the dual XH–π interaction in the HFIP/PhH complex, the electron correlation energy (*E*_corr_) was calculated by the difference of *E*_CCSD(T)/CBS_ and *E*_HF/CBS_. It was found that the electron correlation energy is actually an important component in the interaction energy in HFIP/PhH (*E*_corr_ = −7.47 kcal/mol), CHCl_3_/PhH (*E*_corr_ = −7.16 kcal/mol), and IP/PhH (*E*_corr_ = −6.00 kcal/mol).

**Figure 2:**
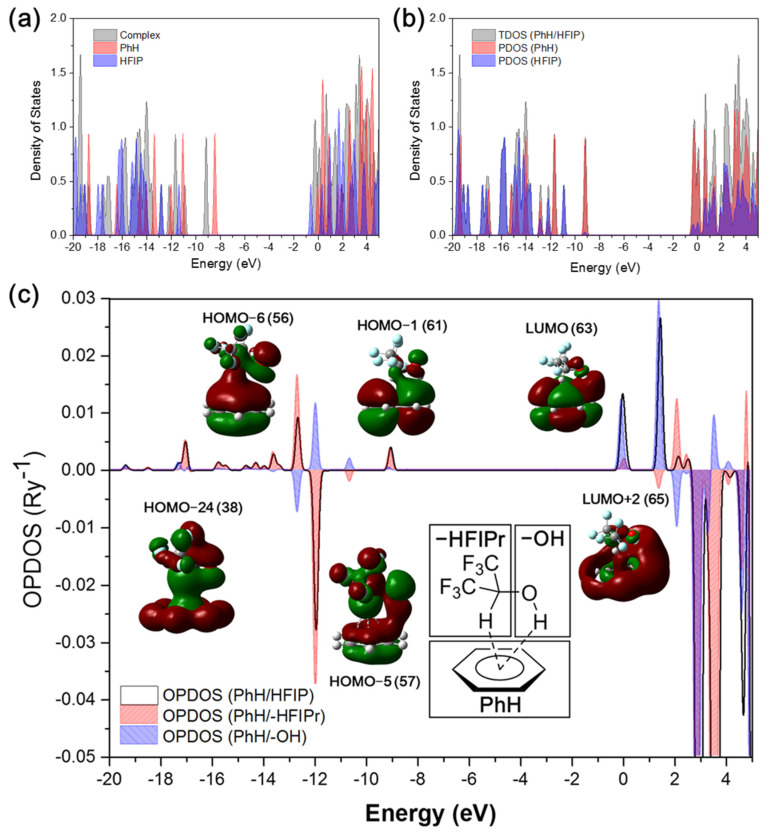
(**a**) The DOS of benzene (PhH), HFIP, and PhH/HFIP complex, and (**b**) the PDOS of the PhH/HFIP complex using M06-2X/6-311++G(2d,2p). (**c**) The OPDOS of the Ph/HFIP complex (black line), PhH/-HFIP (red), and PhH/-OH (blue).

The correlation energies are mainly composed of dispersion interaction; thus, the results containing the dispersion forces play an important role in the dual XH–π interaction between HFIP and benzene. In order to reveal the effect of HFIP on the molecular orbital of benzene, the density of states (DOS) of isolated benzene, isolated HFIP, and the PhH/HFIP complexes were plotted as in Figure 2a with the Hirshfeld method. The gray area shows the DOS of the PhH/HFIP complex, where the energy level of HOMO of benzene (−8.41 eV) is significantly lower from the red peak to the gray peak (−9.14 eV) after the formation of the complex. This phenomenon indicates that the energy level of the HFIP/PhH complex is not a simple superposition of HFIP and benzene. Most of the energy levels of the HFIP/PhH complex are largely decreased about −0.73 eV, which shows the dual XH–π interaction between HFIP and benzene induced the dipole–quadrupole interaction. In Figure 2b, the total DOS (TDOS) of the HFIP/PhH complex, and partial DOS (PDOS) of benzene and HFIP are shown as a different color. Peaks at −12.17 and −12.81 eV show larger intermolecular orbital overlap, which are the HOMO-5 and HOMO-6 of the HFIP/PhH complex. The results show both HFIP and benzene contributing to the HOMO-5, HOMO-6, and higher unoccupied orbitals. To explore the intermolecular orbital interaction between HFIP and benzene, the overlap population DOS (OPDOS) of HFIP/PhH was calculated at the M06-2X/6-311G(2d,2p) level with Mulliken orbital composition method, since the diffuse function should be avoided. The result of the OPDOS is shown in Figure 2c as the black line. Here, we notice that LUMO+2 (1.42 eV), LUMO (−0.04 eV), HOMO-1 (−9.06 eV), HOMO-6 (−12.68 eV), and HOMO-24 (−17.05 eV) show positive values in OPDOS, indicating the bonding orbital between HFIP and benzene. On the opposite, HOMO-5 (−11.96 eV) shows a negative value, indicating a net antibonding property between HFIP and benzene, while the OH bond shows a good overlap with the MO of benzene.

To evaluate the contribution of orbital overlap of the –OH group and –HFIPr group in the HFIP to the MO of benzene separately, partial orbital population DOS (POPDOS) was performed on the PhH/-HFIPr (Figure 2c, red area) and the PhH/-OH (Figure 2c, blue area) fragments. HOMO-5 shows bonding orbital overlap between PhH/-OH orbitals (blue), and antibonding between PhH/-HFIPr with a larger area. Therefore, the total OPDOS shows a negative value. However, even though the total OPDOS shows a negative value, the delocalization and constructive orbital overlap over PhH/-OH still contribute to lowering the electronic energy of MO [43,44] such as the energy change of HOMO-5 of the PhH/HFIP complex shown in Figure 2a. HOMO-6 shows the opposite results in the orbital overlap of PhH/-HFIPr and PhH/-OH to compared with HOMO-5. HOMO-24 and HOMO-1 also show positive values in the total OPDOS and POPDOS (PhH/-HFIPr), indicating the constructive orbital interaction. The LUMO and LUMO+2 also show strong positive values in OPDOS.

**Figure 3:**
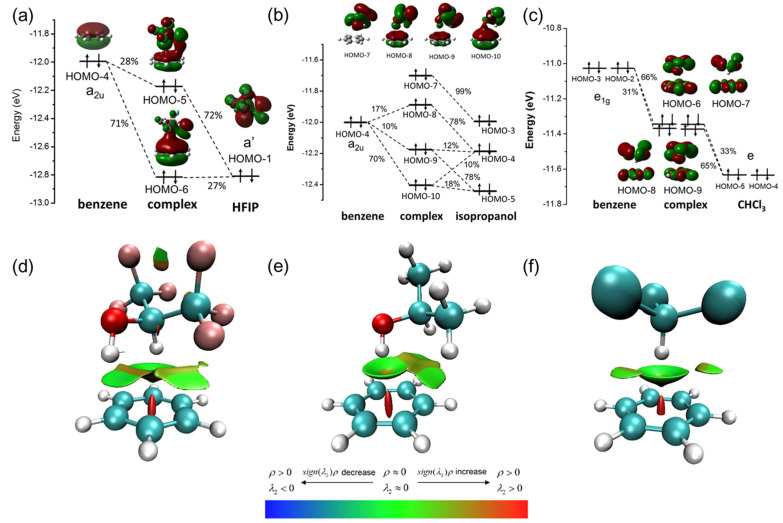
The CDA of (**a**) HFIP/PhH complex, (**b**) IP/PhH complex, and (**c**) CHCl_3_/PhH complex, and RDG isosurfaces of (**d**) HFIP/PhH complex, (**e**) IP/PhH complex, and (**f**) CHCl_3_/PhH complex.

Strong orbital interaction between HFIP/PhH was revealed by OPDOS as described above, and the exact orbital contribution was calculated by performing charge decomposition analysis (CDA) for the complex of HFIP/PhH. As shown in Figure 3a, it shows that HOMO-1 (a’) of HFIP is hybridized with HOMO-4 (a_2u_) of benzene to form HOMO-5 and HOMO-6 of the HFIP/PhH complex. HOMO-1 of HFIP contributes 72% to HOMO-5 and 27% to HOMO-6 of the complex, and HOMO-4 of benzene contributes 28% to HOMO-5 and 71% to HOMO-6 of the complex. The energy levels of HOMO-5 and HOMO-6 of the HFIP/PhH complex are lower than that of HOMO-4 of the isolated benzene. The total orbital energy change of the hybridization with HOMO-1 of HFIP is −0.277 eV. The CDA of IP/PhH (Figure 3b), and CHCl_3_/PhH (Figure 3c) were also calculated to be compared with the HFIP/PhH complex. The IP/PhH complex, HOMO-4 (a_2u_) of benzene, HOMO-4 and HOMO-5 of an IP are hybridized into three MOs (HOMO-8, HOMO-9, HOMO-10) of the IP/PhH with higher energy levels. The total orbital energy change is 0.162 eV, and these MOs show highly delocalized on the two molecules. However, only HOMO-10 shows a constructive orbital overlap between the OH group of IP and the benzene. In addition, the CHCl_3_/PhH complex shows two groups of two degenerate states (HOMO-9 to HOMO-6) due to the high symmetry of CHCl_3_, benzene, and the complex. These MOs, HOMO-9 to HOMO-6, are highly delocalized on CHCl_3_ and the benzene. It should be noted that here, the MOs are hybridized by e_1g_ orbitals of benzene with the e orbital of CHCl_3_ instead of the a_2u_ orbital of benzene.

The reduced density gradient (RDG) is introduced to visualize the intermolecular interaction between complexes (Figure 3d–f) with VMD [33]. In Figure 3d, the interaction of O-H of HFIP with benzene shows the green disk-shaped isosurface, and the interaction of C-H of HFIP with benzene shows the green bowl-shaped isosurface. It indicates that the interaction between HFIP and benzene is close to van der Waals force, and the observed isosurface supports the existence of a dual XH–π interaction between HFIP and benzene. In Figure 3e, a green bowl-shaped isosurface at the front of O-H of IP shows van der Waals interaction, and a brown isosurface between C-H of IP and benzene shows repulsion interaction. In Figure 3f, a green bowl-shaped isosurface between CHCl_3_ and benzene shows van der Waals forces.

**Figure 4:**
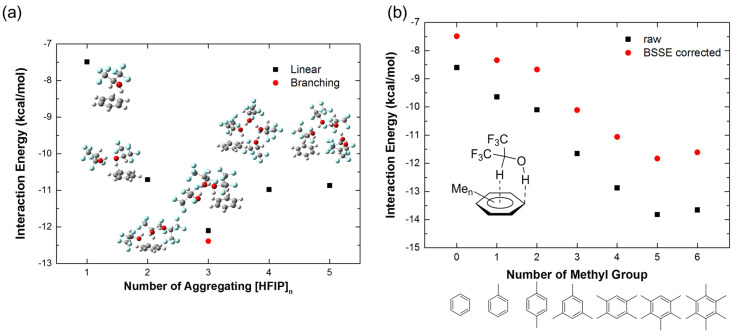
The interaction energies (kcal/mol) of (**a**) [HFIP]n/PhH complexes (n = 1–5) with BSSE correction and (**b**) HFIP/PhMen (n = 0–6) with and without BSSE correction.

In addition, the aggregation effect of [HFIP]_n_ is also considered in the calculation of interaction energies of HFIP with benzene. The interaction energies of different numbers of aggregating [HFIP]_n_ (n = 1–5) with benzene are shown in Figure 4a. The interaction energy of three HFIPs in the branched structure with a benzene molecule shows the strongest interaction (−12.39 kcal/mol) to compare with the linear connected HFIP (−12.10 kcal/mol), and other aggregating numbers. The interaction energies of benzene with two, four, and five HFIP molecules are near to −11 kcal/mol with 0.2 kcal/mol differences. Therefore, two molecules of HFIP are sufficient to describe the enhancement of interaction energy with benzene caused by the aggregating effect of [HFIP]_n_. Moreover, the delocalization of orbitals of benzene to [HFIP]_n_ molecules significantly lowers the orbital energy levels of benzene, as shown in the CDA diagram (n = 2–5 in Figures S1–S5, in Supplementary Materials), which also shows [HFIP]_n_ are involved in the hybridization of MOs of complexes of [HFIP]_n_/PhH. Highly delocalized molecular orbitals are observed in the aggregating model, as shown in Figures S6–S11.

The interaction energies between HFIP and different numbers of methyl-substituted benzene, including toluene, *p*-xylene, mesitylene, durene, pentamethylbenzene (PMB), and hexamethylbenzene (HMB), are compared in Figure 4b (for optimized structures, see Figure S15). It shows that the interaction energy turns more negative when more methyl groups appear in benzene, indicating an electron-rich π-system to favorably form a complex with HFIP. The HFIP/PMB shows the strongest interaction energy with BSSE correction (−11.83 kcal/mol, BSSE = −1.99 kcal/mol), which is slightly stronger than HMB due to closer contact and a better docking shape between HFIP and PMB, while HMB has higher steric hindrance to increase the interaction distance, resulting in the decrease in intermolecular dispersion forces.

In addition, the interaction energies of HFIP, IP, and CHCl_3_ with selected common organic compounds bearing typical functional groups were also calculated at M06-2X/6-311++G(2d,2p) level with consideration of the basis set supposition error (Table 2, and see Table S1 for raw values, and all optimized structures can be found in Figures S12–S14). From entries 1 to 3, the absolute values of interaction energies of alkane, alkene, and alkyne with HFIP in order are acetylene > ethylene > ethane. Alkyne shows strongest interaction (−5.87 kcal/mol) between HFIP due to the electron-rich π-bonds. The electron density on the ethylene is smaller than alkyne, leading to the smaller interaction energy between ethylene/HFIP (−5.31 kcal/mol). Ethane shows very weak interaction (−1.50 kcal/mol) with HFIP due to no electron-rich π-bond on the alkane group. To compare with HFIP, IP shows smaller interaction energies from entries 1 to 3. Even acetylene only gives -3.16 kcal/mol interaction energy. CHCl_3_ also gives results similar to IP. These results indicate that HFIP has a strong XH–π interaction with the π-bond system.

Entry 4 shows the interaction energy between benzene and HIFP is very strong (−7.49 kcal/mol), which is much higher than the hydrogen bond of water (−3.16 kcal/mol) [45], OH–π of H_2_O/benzene (−3.19 kcal/mol) [46], parallel-alignment interaction of H_2_O/benzene (−1.60 kcal/mol) [47], and H_2_O-HC of H_2_O/benzene (−1.38 kcal/mol) [48]. The interaction energies between benzene and IP (−4.53 kcal/mol) and benzene and CHCl_3_ (−5.65 kcal/mol) are medium, resulting from their polar–π interaction.

From entries 5 to 24, the interaction energies of the selected organic compounds bearing typical functional groups with HFIP, IP, CHCl_3_ are concluded, and only the interaction energies with HFIP are discussed in detail.

In entries 5 and 6, HMB is an electron-rich aromatic π-system, and hexafluorobenzene (HFB) is used as an electron-deficient aromatic π-system. HMB shows very strong interaction with HFIP with −11.61 kcal/mol interaction energy, and the scale of the interaction energy is comparable to ion–dipole interaction. As expected, the electron-deficient HFB shows weak interaction with HFIP, and the interaction energy is in the range between ethane/HFIP and ethylene/HFIP.

In entries 7 and 8, anisole and naphthalene are used as different types of electron-rich π-system. The interaction energies of anisole and naphthalene with HFIP are −8.60 kcal/mol and −7.71 kcal/mol, respectively. Noted that the interaction energies of anisole and naphthalene with HFIP or IP, or CHCl_3_ are stronger than those of HFIP/benzene, IP/benzene and CHCl_3_/benzene.

Entries 9 to 11 show the interaction energies of cycloalkanes. The absolute values of interaction energies with HFIP in order are cyclopropane > cubane > cyclohexane. The trend reveals that the highly bent C-C bond in hydrocarbons contributes to the higher composition of *p*-orbital character to interact with HFIP. The trend shows little difference in IP and CHCl_3_. With IP or CHCl_3_, the absolute value of interaction energies in order are cubane > cyclopropane > cyclohexane, due to the better staking of IP and CHCl_3_ by cubane.

From entries 12 to 24, water, ethers, sulfoxides, ketones, acetic acid, amine, nitriles, and amides, regarded as common hydrogen bond acceptors, are investigated. In entries 12 to 17, HFIP shows interaction energies with sp^3^ oxygen in water and ethers in the range from −9.83 to −12.21 kcal/mol. Ether contains electron-rich alkyl groups showing stronger interaction with HFIP than water. Only in the case of ethylene oxide, it shows a slightly smaller interaction energy than water.

Entries 18 to 21 show the interaction of sp^2^ hybridized oxygen of DMSO, sulfolane, acetone, and acetic acid with HFIP, IP and CHCl_3_. The results show that the interaction between DMSO and HFIP is quite high, and the interaction energy is −15.62 kcal/mol, which results mainly from the strong dipole–dipole interaction. Sulfolane shows slightly weaker interaction energy (−12.94 kcal/mol) compared to DMSO, due to the decrease in electron density on the sulfur atom. Oxygen atoms on C=O group in acetone and acetic acid show similar strength interaction with HFIP (−11.88 and −11.74 kcal/mol).

In entry 22, trimethylamine, as a common organic base and a traditional hydrogen bond acceptor, forms a stronger hydrogen bond interaction with HFIP, with −15.09 kcal/mol interaction energy. The nitrogen atom on acetonitrile provides unpaired electrons as a hydrogen bond acceptor with HFIP with −9.52 kcal/mol interaction energy, similar to H_2_O (entry 23). Dimethylacetamide (DMAc) is a common high polar solvent, which can also form a strong hydrogen bond with HFIP (−14.88 kcal/mol) (entry 24).

In general, the interaction energies concluded in Table 2 between HFIP and common organic compounds are usually larger than those from IP and CHCl_3_, excluding the electron-deficient compound of HFB, and saturated alkane of ethane. The interaction energies of HFIP with the selected 24 compounds are 1.48 ± 0.35 times of IP, and 1.61 ± 0.48 times of CHCl_3_, confirming the dual XH (OH and CH) hydrogen-bond-donating property of HFIP.

**Figure 5:**
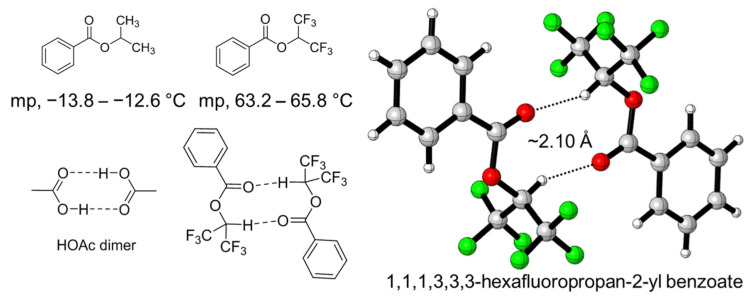
HFIPBz dimer observed by single-crystal X-ray diffraction, the measured melting point of HFIPBz and isopropyl benzoate.

On the other hand, single-crystal X-ray diffraction is the most straight forward and important technique in the structural determination of crystalline materials for understanding their structure–property relationships. The XH–π interaction between HFIP and arenes can be found in many reported crystal structures. For example, Halmiton et al. reported the crystal structures containing macrocyclics and HFIP, and a very short distance of CH–π between HFIP and the arene in macrocyclics can be found (2.28–2.28 Å) [49]. To experimentally observe the hydrogen-bond-donating property of the C-H bond in HFIP without the influence of the O-H bond, the benzoyl group was introduced to mask the O-H group in HFIP, and the corresponding ester, 1,1,1,3,3,3-hexaflurorisopropyl benzoate (HFIPBz), was prepared. Surprisingly, HFIPBz shows a greatly higher melting point (63.2–65.8 °C), compared to its analogue of isopropyl benzoate (IPBz) (−13.8–−12.6 °C). The unexpected high melting point apparently indicates the strong interaction among of HFIPBz with the high degree of molecular orientation due to the hydrogen bonding, resulting in the formation of HFIPBz dimer, as shown in Figure 5 [50]. The hydrogen bond between C-H of one hexafluoroisopropyl group and oxygen atom of carbonyl group in another HFIPBz is shown to be 2.10 Å.

## 4. Conclusions

The X-H-donating property of HFIP has been studied with arenes and various common organic compounds in the computational method with the high-accuracy CCSD(T)/CBS method and M06-2X/6-311++G(2d,2p), showing comparable accuracy with a lower computational cost. The dual X-H-donating property of HFIP shows significant interaction with arenes to form dual XH–π interaction, which is even higher than the interaction energy of water dimer, and shows the importance of the unusual interaction. Strong dispersion forces were revealed from the HFIP/PhH complex by post-HF methods. Charge decomposition analysis, TDOS, PDOS, and OPDOS reveal strong orbital interactions between HFIP and benzene which largely decrease the energy level of molecular orbitals of benzene. The aggregating effect of [HFIP]_n_ delocalizes electrons from benzene that significantly lower the energy level of MOs of benzene. HFIP are shown to have stronger interaction energy and dual XH–π interaction with electron-rich arenes such as multi-methyl-substituted benzenes, naphthalene, and anisole. Some molecules with strong dipole moment, such as DMSO, acetone, DMAc, and acetonitrile, show strong interaction energies with HFIP. Hydrocarbons with higher unsaturation numbers and higher ring strain show stronger interaction energy with HFIP. The XRD of HFIPBz shows the crystal structure in which the intermolecular hydrogen bonds between the C-H group on HFIP and the carbonyl group on another HFIPBz molecule are observed. These findings offer insight into the nature of the O-H and C-H bonds of HFIP as the dual hydrogen bond donor interacting with arenes, and the strong dual XH–π interaction between HFIP and arenes is expected to affect the reactivity manner and the mechanism of arenes for opening the new transformation or functionalization of arenes [51].

## Figures and Tables

**Table 1:** Electronic interaction energies (E_int_), including counterpoise corrections.

Method	HFIP/PhH	CHCl_3_/PhH	IP/PhH
HF/aug-cc-pVDZ	0.28	1.26	1.71
HF/aug-cc-pVTZ	0.26	1.21	1.66
HF/CBS *^a^*	0.25	1.19	1.64
MP2/aug-cc-pVDZ	−6.88	−5.99	−4.21
MP2/aug-cc-pVTZ	−7.79	−6.89	−4.86
MP2/CBS *^b^*	−8.17	−7.27	−5.13
MP2/6-31+G(d,p)	−5.25	−3.65	−2.58
MP2/6-311++G(d,p)	−5.69	−4.33	−3.16
CCSD(T)/6-31+G(d,p)	−4.29	−2.49	−1.88
CCSD(T)/6-311++G(d,p)	−4.74	−3.03	−2.39
Δ(CCSD(T)-MP2) *^c^*	0.96	1.16	0.70
Δ(CCSD(T)-MP2) *^d^*	0.95	1.30	0.77
CCSD(T)/CBS *^e, c^*	−7.21	−6.11	−4.43
CCSD(T)/CBS *^e, d^*	−7.22	−5.97	−4.36
*E*_corr_ *^f^*	−7.47	−7.16	−6.00
ΔZPE *^g^*	0.85	0.55	0.54
M06-2X/6-311++G(2d,2p) *^h^*	−7.49	−5.65	−4.53
*D*_0_ (calculated) *^i^*	6.64	5.10	3.99
*D*_0_ (experimental)		5.2 ± 0.2 *^j^*	

*^a^* Extrapolated from HF/aug-cc-pVDZ and HF/aug-cc-pVTZ with the method of Truhlar et al. [39,40,41]. ^*b*^ Extrapolated from MP2/aug-cc-pVDZ and MP2/aug-cc-pVTZ with the method of Truhlar et al. [39,40,41]. *^c^* The correction term was calculated with 6-31+G(d,p) basis set. *^d^* The correction term was calculated with 6-311++G(d,p) basis set. *^e^* The value of CCSD(T)/CBS was obtained by the equation: *E*_CCSD(T)/CBS_ = *E*_MP2/CBS_ + *E*_Δ(CCSD(T) − MP2)_ [39,42]. *^f^* The electron correlation energy, *E*_corr_, was calculated by the equation, *E*_corr_ = *E*_CCSD(T)/CBS_ − *E*_HF/CBS_. *^g^* Difference of zero-point vibrational energy by the formation of complex calculate with M06-2X/6-311++G(d,p) method. *^h^* The best ranking method in the benchmark of hydrogen bond–π of Ramos et al. [30]. *^i^* Binding energy of complex (*D*_0_ = *D*_e_ − ΔZPE, *D*_e =_ *E*_int_). *^j^* Binding energy of the CHCl_3_/PhH complex [38].

**Table 2:** Interaction energies of HFIP, isopropanol (IP), and chloroform (CHCl_3_) between common molecules calculated at M06-2X/6-311++G(2d,2p) level with BSSE correction.

		X-H Donor		
Entry	X-H Acceptor	HFIP	IP	CHCl_3_
1	ethane	−1.50	−2.49	−2.24
2	ethylene	−5.31	−3.23	−3.27
3	acetylene	−5.87	−3.16	−2.80
4	benzene	−7.49	−4.53	−5.65
5	HMB	−11.61	−7.80	−9.14
6	HFB	−2.71	−4.61	−2.96
7	anisole	−8.60	−5.51	−6.63
8	naphthalene	−7.71	−5.79	−6.25
9	cyclohexane	−3.80	−2.11	−2.34
10	cyclopropane	−5.42	−3.43	−2.06
11	cubane	−5.26	−3.64	−3.89
12	H_2_O	−9.83	−6.52	−4.64
13	Me_2_O	−10.45	−6.20	−5.41
14	Et_2_O	−12.21	−7.60	−6.81
15	THF	−11.71	−7.42	−6.34
16	1,4-dioxane	−10.38	−6.46	−5.21
17	ethylene oxide	−9.60	−6.21	−5.62
18	DMSO	−15.62	−9.38	−7.24
19	sulfolane	−12.94	−6.24	−6.17
20	acetone	−11.88	−7.06	−6.01
21	acetic acid	−11.74	−5.63	−6.38
22	trimethylamine	−15.09	−8.41	−6.50
23	acetonitrile	−9.52	−4.90	−3.94
24	DMAc	−14.88	−8.83	−7.94

## Data Availability

Not applicable.

## Supplementary Materials

The following are available online. The computational studies on the complexation energies of complexes, charge decomposition analysis, molecular orbitals of complexes, optimized geometries and cartesian coordinates of complexes, and the X-ray structural details of 1,1,1,3,3,3-hexaflurorisopropyl benzoate (HFIPBz).

## Sample Availability

The sample of 1,1,1,3,3,3-hexaflurorisopropyl benzoate (HFIPBz) is available from the authors.
