# Supplementary material for: Dual XH–π Interaction of Hexafluoroisopropanol with Arenes
Source: Molecules. 2021 Jul 28;26(15):4558. doi: 10.3390/molecules26154558 (PMC8347120; doi:10.3390/molecules26154558)
Supplement: Supplementary file 1 [file molecules-26-04558-s001.zip › molecules-1306653-supplementary.pdf]

Supplementary Information for

Dual XH–π Interactions of Hexafluoroisopropanol with Arenes

Le Lu and Ruimao Hua*

Department of Chemistry, Tsinghua University, Key Laboratory of Organic Optoelectronics & Molecular Engineering of Ministry of Education, Beijing 100084, China

Contents:

1. Complexation energies of complexes 3

Table S1. Complexation energies of HFIP, isopropanol (IP), and chloroform between common H-bond acceptors calculated at M06-2X/6-311++G(2d,2p) level with counterpoise correction (kcal/mol). 3

2. Charge decomposition analysis 4

Figure S1. The charge decomposition analysis of the $[HFIP]_2$/PhH complex. 4
Figure S2. The charge decomposition analysis of the branched $[HFIP]_3$/PhH complex. 5
Figure S3. The charge decomposition analysis of the linear $[HFIP]_3$/PhH complex. 6
Figure S4. The charge decomposition analysis of the $[HFIP]_4$/PhH complex. 7
Figure S5. The charge decomposition analysis of the $[HFIP]_5$/PhH complex. 8

3. Molecular orbitals of complexes 9

Figure S6. Molecular orbitals of the HFIP/PhH complex calculated by M06-2X/6-311++G(2d,2p) theory level. The isovalue is ± 0.02 9
Figure S7. Molecular orbitals of the $[HFIP]_2$/PhH complex calculated by M06-2X/6-311++G(2d,2p) theory level. The isovalue is ± 0.02 9
Figure S8. Molecular orbitals of the branched $[HFIP]_3$/PhH complex calculated by M06-2X/6-311++G(2d,2p) theory level. The isovalue is ± 0.02 10
Figure S9. Molecular orbitals of the linear $[HFIP]_3$/PhH complex calculated by M06-2X/6-311++G(2d,2p) theory level. The isovalue is ± 0.02 11
Figure S10. Molecular orbitals of the $[HFIP]_4$/PhH complex calculated by M06-2X/6-311++G(2d,2p) theory level. The isovalue is ± 0.02 12
Figure S11. Molecular orbitals of the $[HFIP]_5$/PhH complex calculated by M06-2X/6-311++G(2d,2p) theory level. The isovalue is ± 0.02 13

4. Optimized geometries and cartesian coordinates of complexes 14

Figure S12. Optimized geometries of HFIP/H-bond acceptors calculated by M06-2X/6-311++G(2d,2p) theory level. 14
Figure S13. Optimized geometries of IP/H-bond acceptors calculated by M06-2X/6-311++G(2d,2p) theory level. 15
Figure S14. Optimized geometries of $CHCl_3$/H-bond acceptors calculated by M06-2X/6-311++G(2d,2p) theory level. 16
Figure S15. Optimized geometries of HFIP/$PhMe_n$ complexes (n = 1-6) calculated by M06-2X/6-311++G(2d,2p) theory level. 17
Cartesian coordinates of $[HFIP]_n$/PhH complexes 18
Cartesian coordinates of optimized geometries of HFIP/H-bond acceptors 21
Cartesian coordinates of optimized geometries of IP/H-bond acceptors 29
Cartesian coordinates of optimized geometries of $CHCl_3$/H-bond acceptors 38
Cartesian coordinates of optimized geometries of HFIP/PhMen complexes (n = 1-6) 45
Cartesian coordinates of optimized geometries of HFIP/PhH conformers 47

5. Single crystal x-ray diffraction of HFIPBz 49

1. Complexation energies of complexes

Table S1. Complexation energies of HFIP, isopropanol (IP), and chloroform between common H-bond acceptors calculated at M06-2X/6-311++G(2d,2p) level with counterpoise correction (kcal/mol).

		X-H donor					
		HFIP		IP		$CHCl_3$	
entry	X-H acceptor	raw	BSSE corrected	raw	BSSE corrected	raw	BSSE corrected
1	ethane	-1.93	-1.50	-2.72	-2.49	-2.80	-2.24
2	ethylene	-5.75	-5.31	-3.40	-3.23	-3.77	-3.27
3	acetylene	-6.22	-5.87	-3.31	-3.16	-3.22	-2.8
4	benzene	-8.60	-7.49	-4.92	-4.53	-6.51	-5.65
5	HMB	-13.65	-11.61	-8.44	-7.80	-10.83	-9.14
6	HFB	-4.69	-2.71	-5.84	-4.61	-4.51	-2.96
7	anisole	-9.97	-8.60	-6.12	-5.51	-7.82	-6.63
8	Naphthalene	-9.04	-7.71	-6.34	-5.79	-7.46	-6.25
9	Cyclohexane	-4.68	-3.80	-2.41	-2.11	-3.24	-2.34
10	cyclopropane	-6.00	-5.42	-3.66	-3.43	-2.74	-2.06
11	cubane	-6.52	-5.26	-4.16	-3.64	-4.97	-3.89
12	H_2O	-10.51	-9.83	-6.98	-6.52	-5.24	-4.64
13	Me_2O	-11.22	-10.45	-6.70	-6.20	-6.26	-5.41
14	Et_2O	-13.26	-12.21	-8.15	-7.60	-7.96	-6.81
15	THF	-12.62	-11.71	-7.99	-7.42	-7.34	-6.34
16	1,4-dioxane	-11.53	-10.38	-7.06	-6.46	-6.17	-5.21
17	ethylene oxide	-10.53	-9.60	-6.74	-6.21	-6.43	-5.62
18	DMSO	-16.74	-15.62	-10.02	-9.38	-8.12	-7.24
19	sulfolane	-14.35	-12.94	-6.90	-6.24	-7.14	-6.17
20	acetone	-12.73	-11.88	-7.48	-7.06	-6.86	-6.01
21	acetic acid	-12.86	-11.74	-6.08	-5.63	-7.12	-6.38
22	Trimethylamine	-16.14	-15.09	-8.87	-8.41	-7.40	-6.50
23	acetonitrile	-9.92	-9.52	-5.13	-4.90	-4.30	-3.94
24	DMAc	-16.11	-14.88	-9.40	-8.83	-8.97	-7.94

2. Charge decomposition analysis

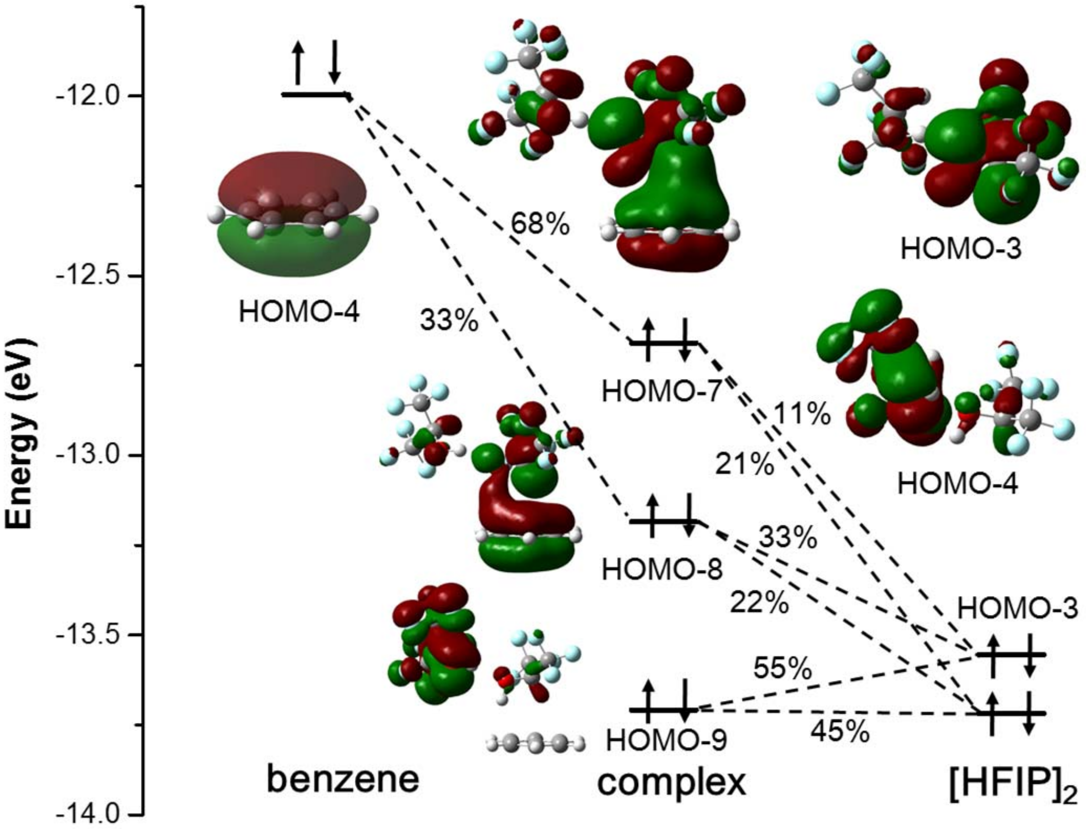

Figure S1. The charge decomposition analysis of the $[HFIP]_2$/PhH complex.

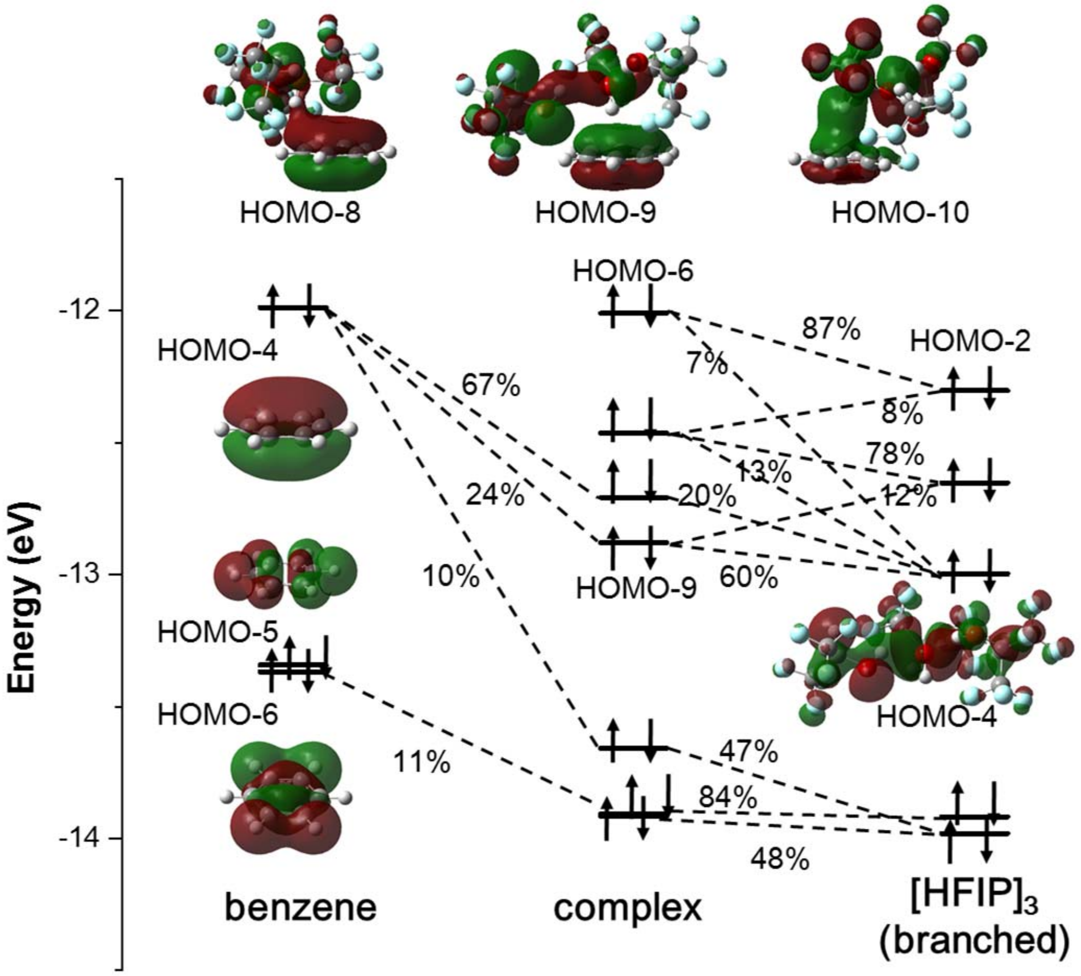

Figure S2. The charge decomposition analysis of the branched $[HFIP]_3$/PhH complex.

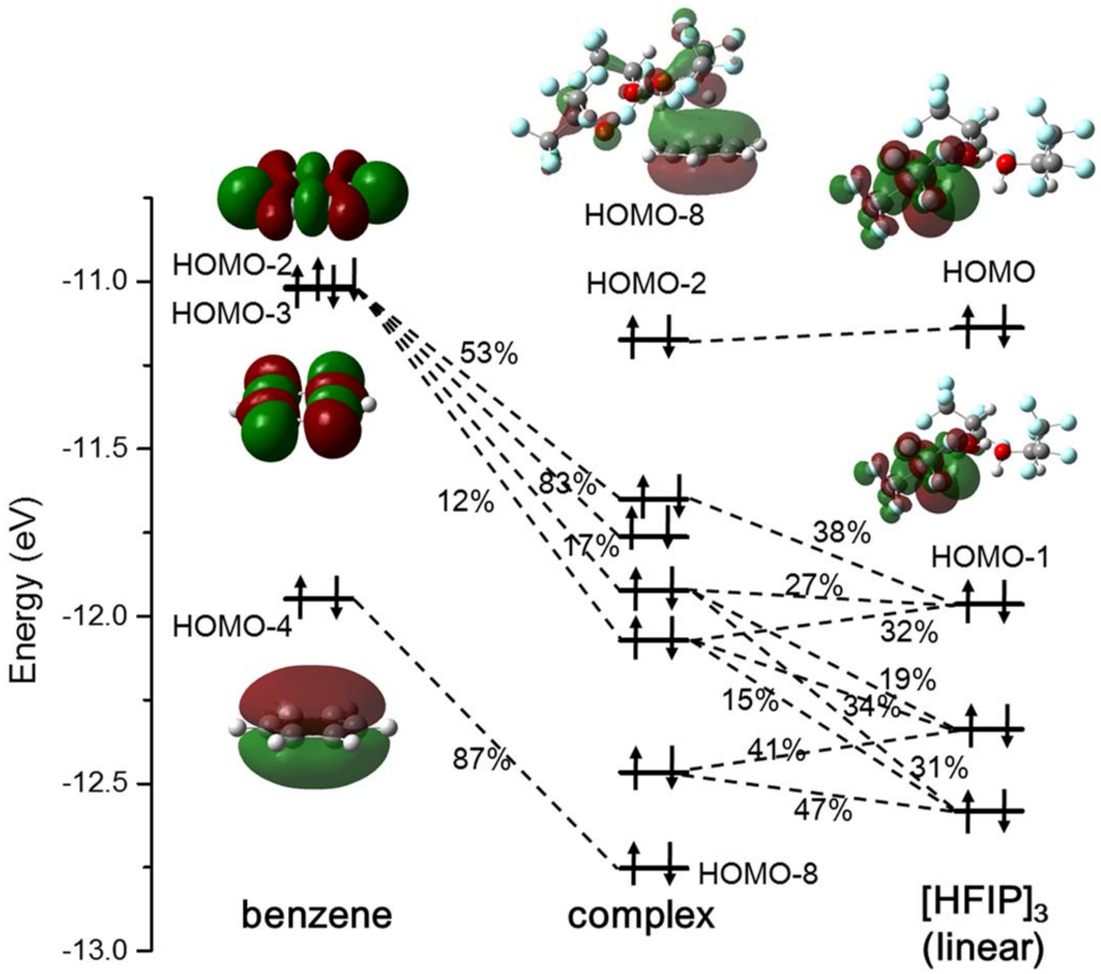

Figure S3. The charge decomposition analysis of the linear $[HFIP]_3$/PhH complex.

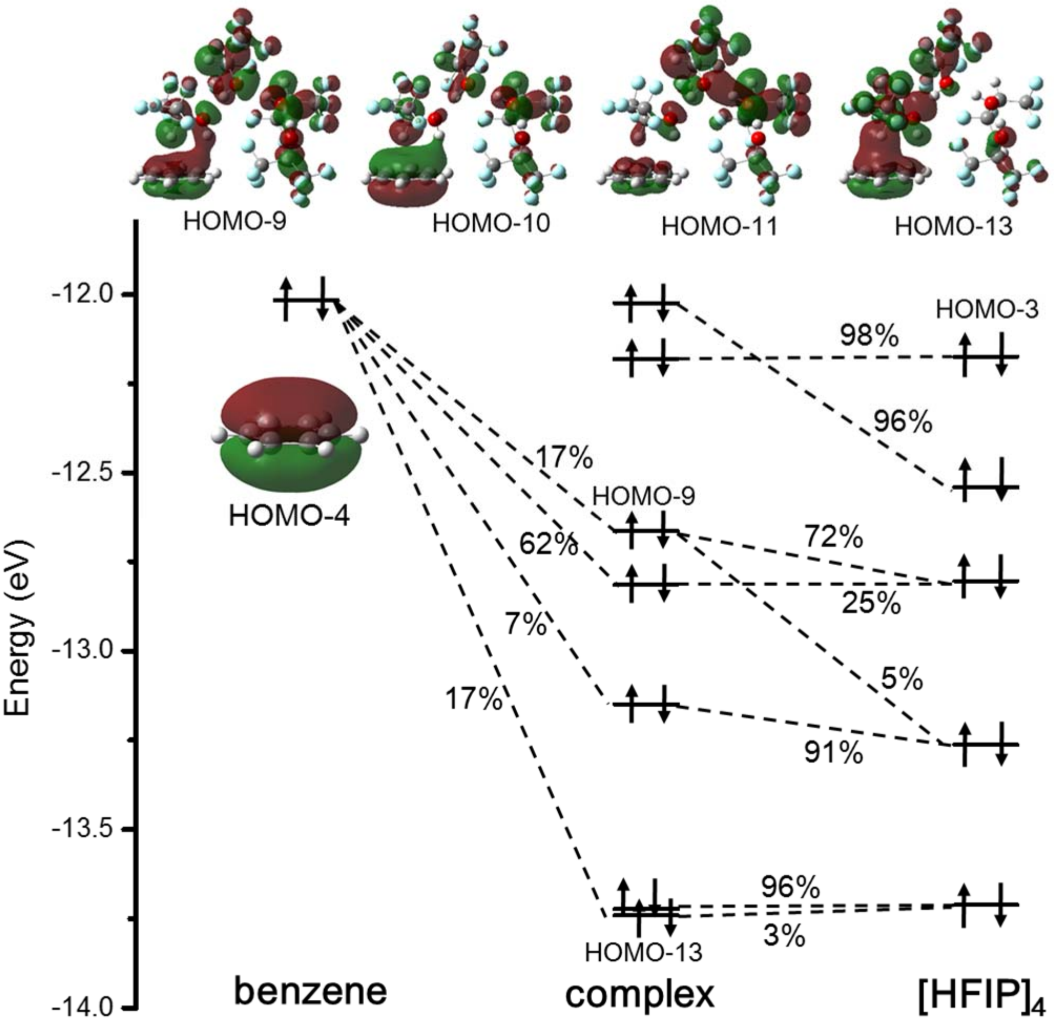

Figure S4. The charge decomposition analysis of the $[HFIP]_4$/PhH complex.

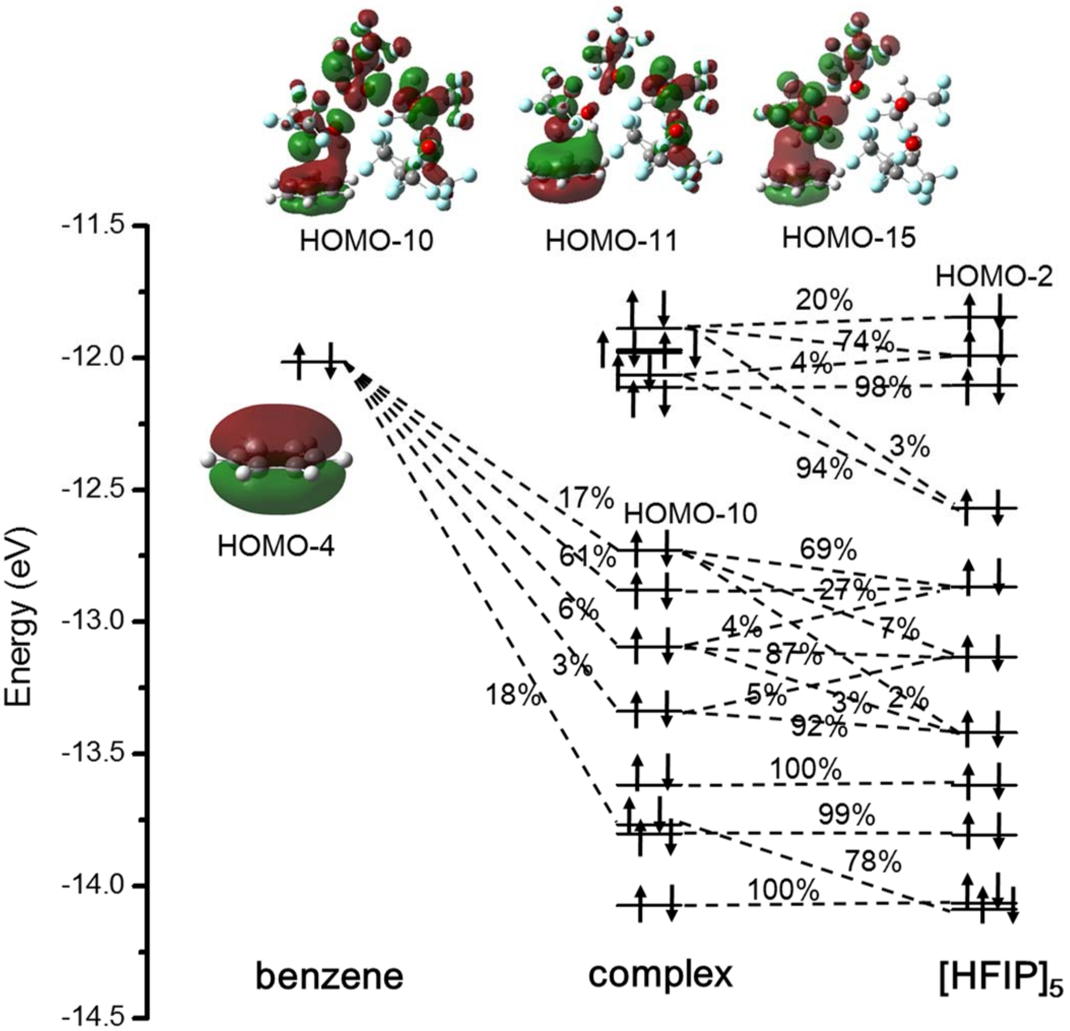

Figure S5. The charge decomposition analysis of the $[HFIP]_5$/PhH complex.

3. Molecular orbitals of complexes

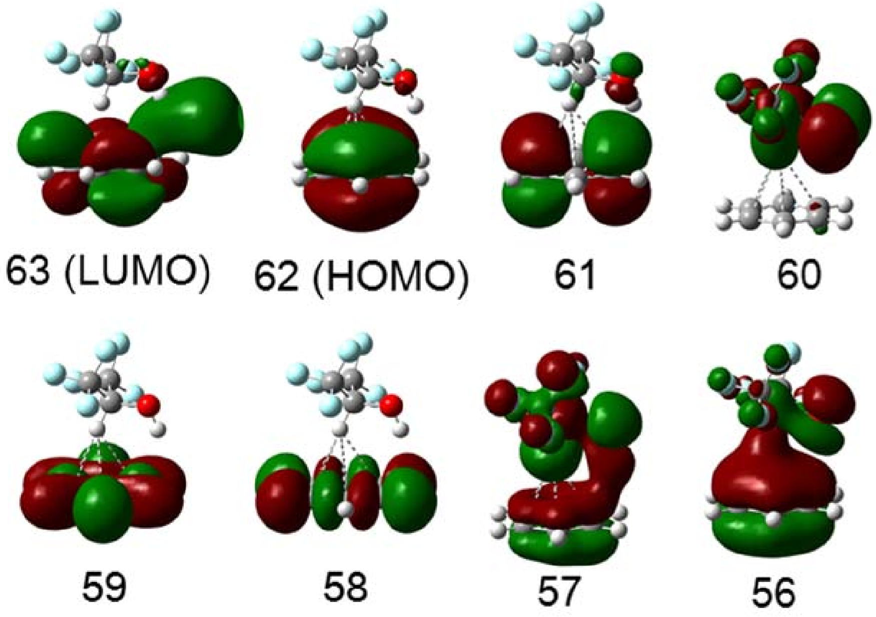

Figure S6. Molecular orbitals of the HFIP/PhH complex calculated by M06-2X/6-311++G(2d,2p) theory level. The isovalue is ± 0.02.

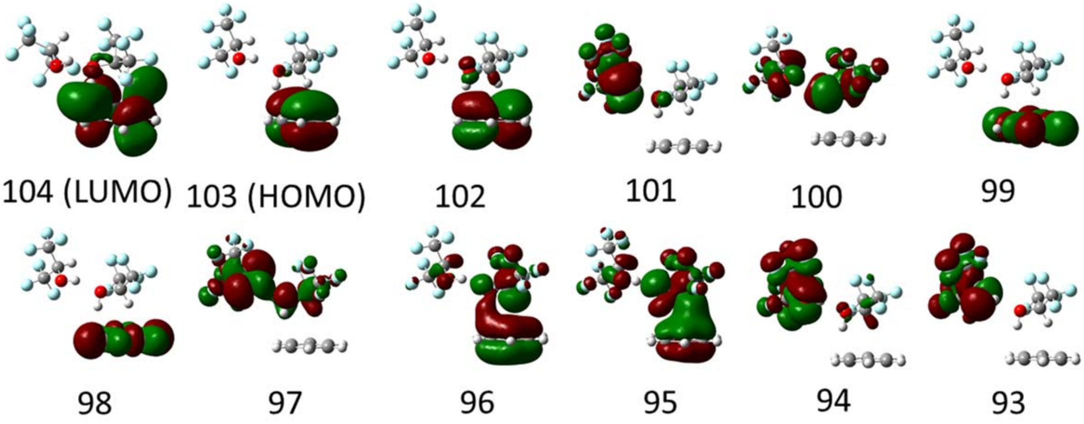

Figure S7. Molecular orbitals of the $[HFIP]_2$/PhH complex calculated by M06-2X/6-311++G(2d,2p) theory level. The isovalue is ± 0.02.

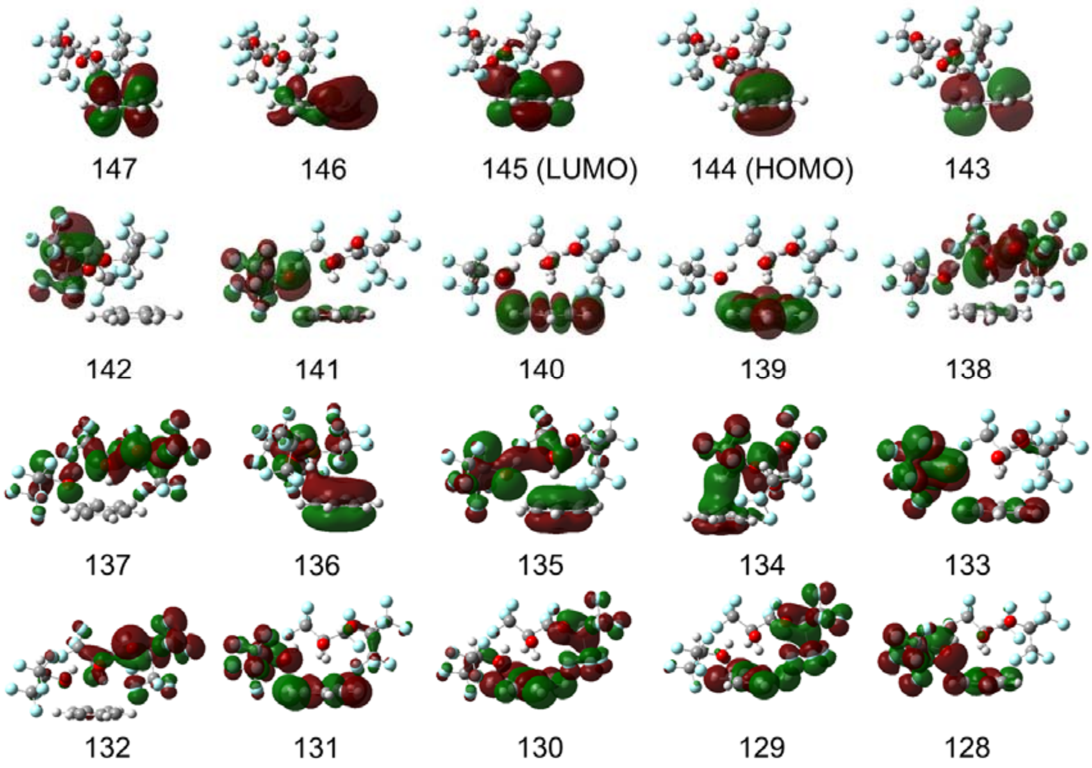

Figure S8. Molecular orbitals of the branched $[HFIP]_3$/PhH complex calculated by M06-2X/6-311++G(2d,2p) theory level. The isovalue is ± 0.02.

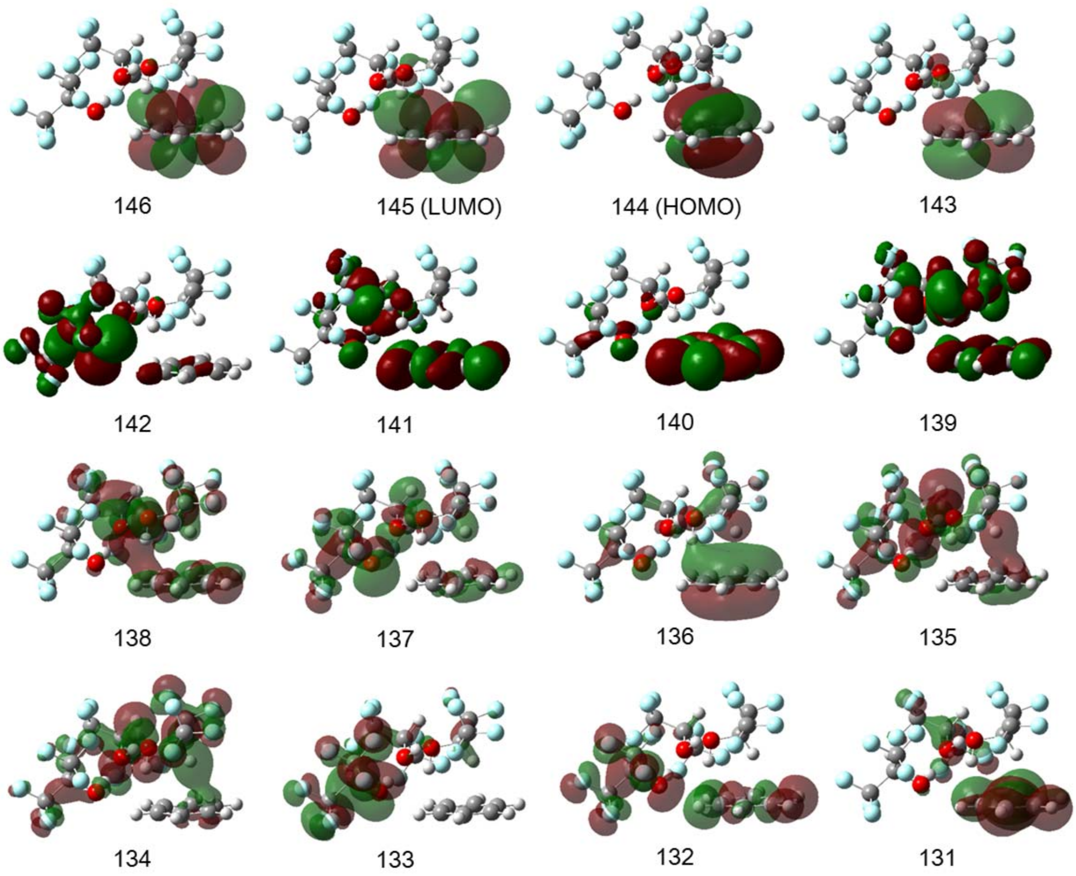

Figure S9. Molecular orbitals of the linear [HFIP]$_3$/PhH complex calculated by M06-2X/6-311++G(2d,2p) theory level. The isovalue is ± 0.02.

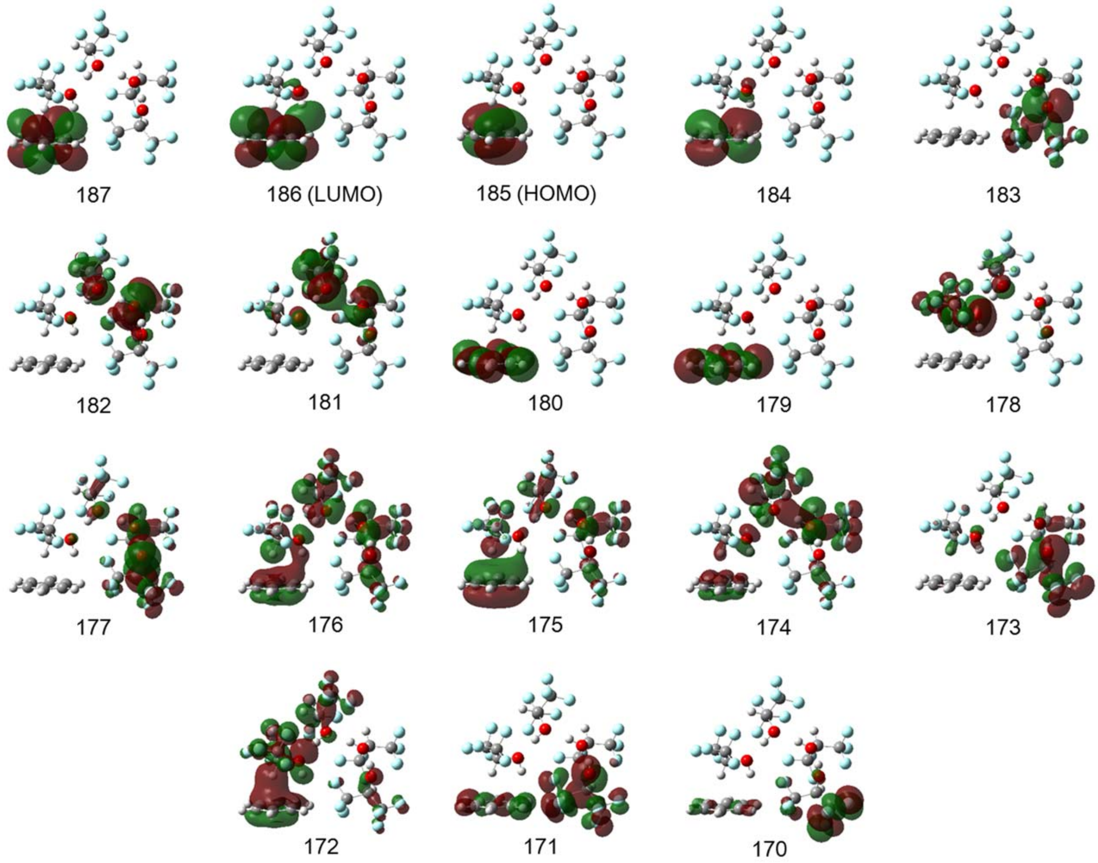

Figure S10. Molecular orbitals of the $[HFIP]_4$/PhH complex calculated by M06-2X/6-311++G(2d,2p) theory level. The isovalue is ± 0.02.

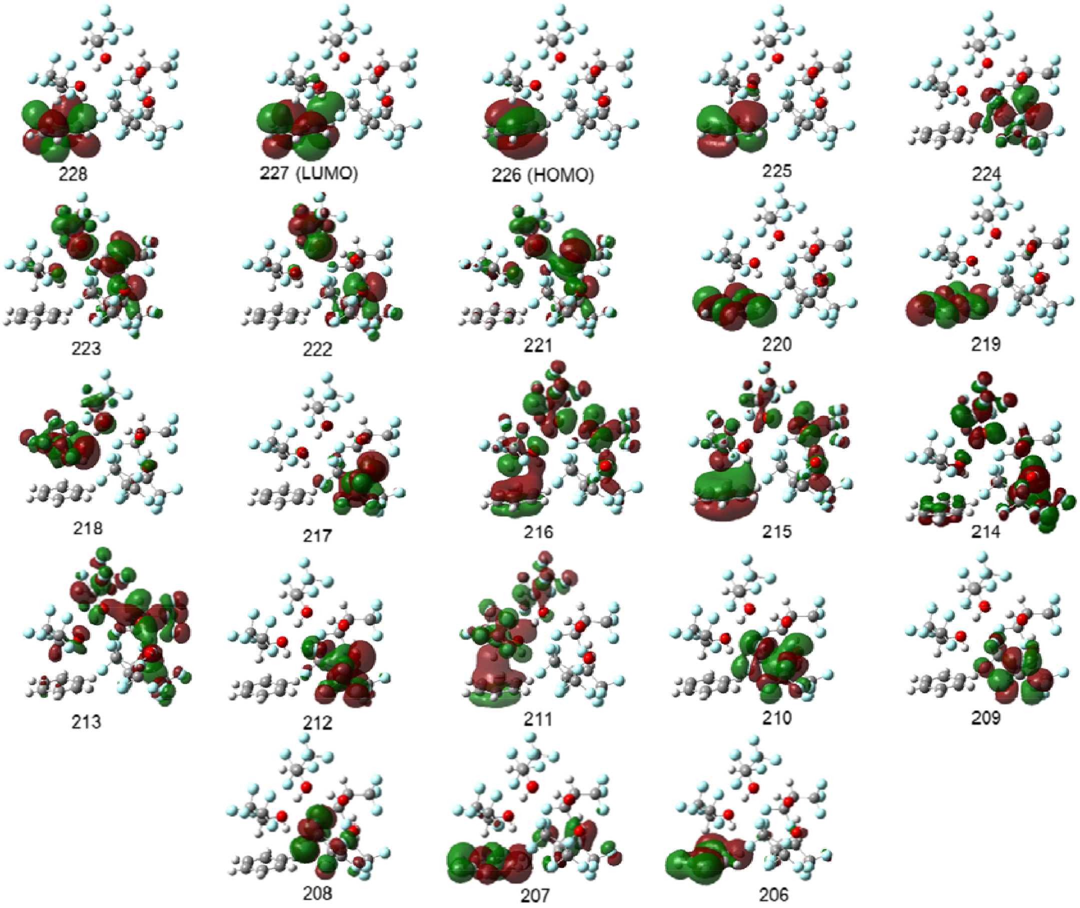

Figure S11. Molecular orbitals of the $[HFIP]_5$/PhH complex calculated by M06-2X/6-311++G(2d,2p) theory level. The isovalue is ± 0.02.

4. Optimized geometries and cartesian coordinates of complexes

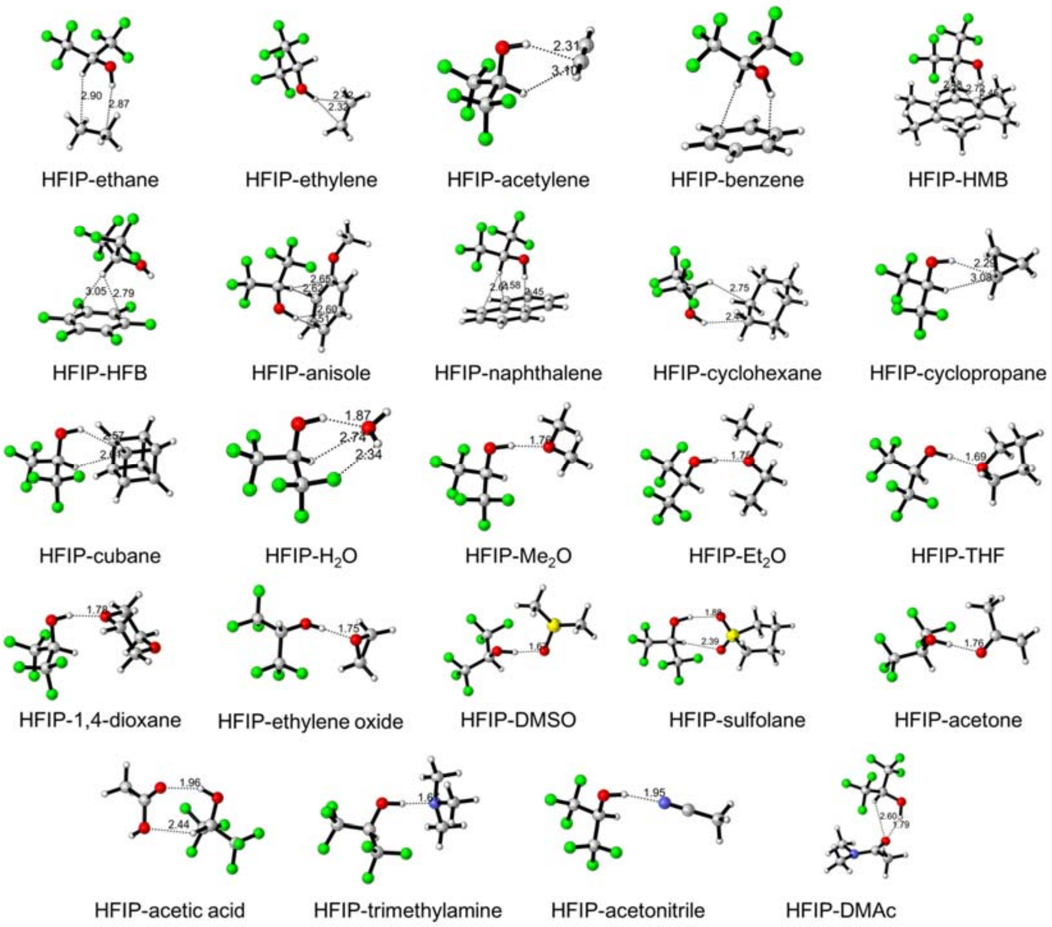

Figure S12. Optimized geometries of HFIP/H-bond acceptors calculated by M06-2X/6-311++G(2d,2p) theory level.

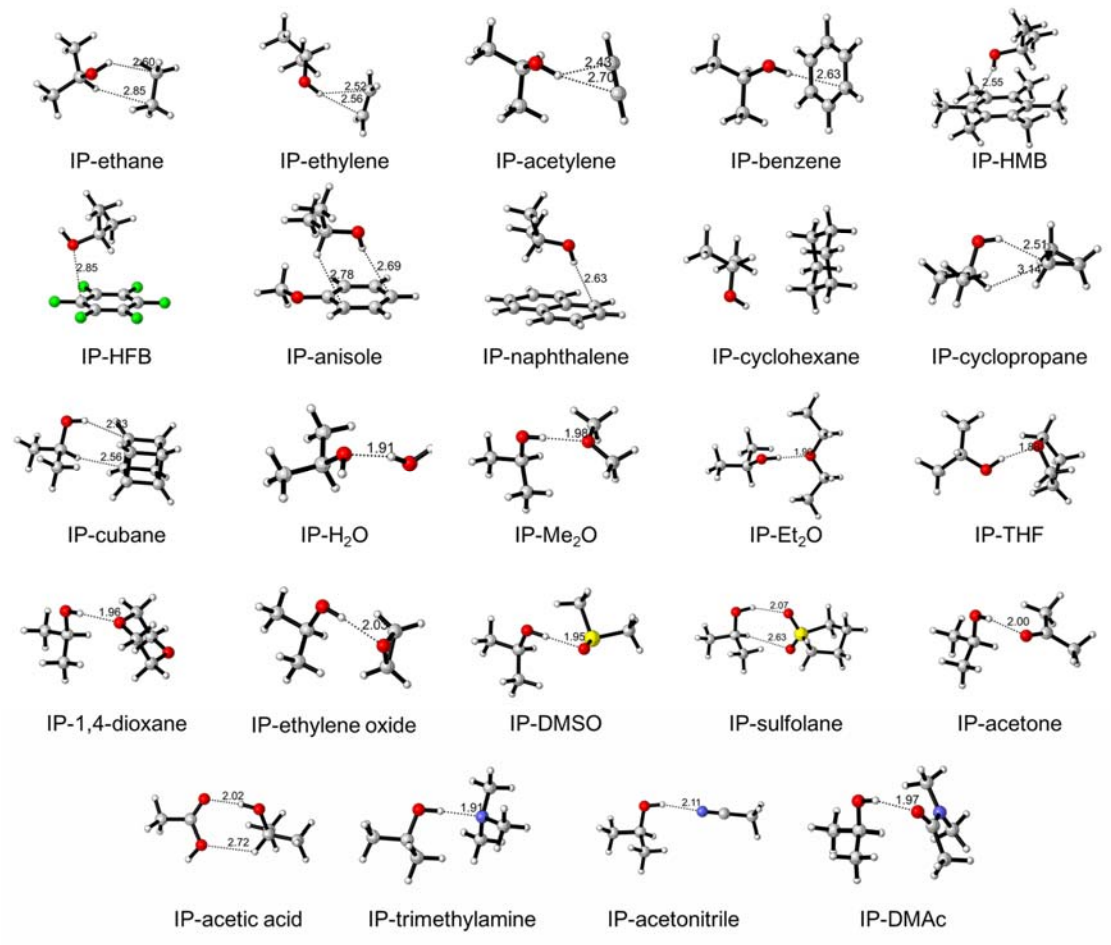

Figure S13. Optimized geometries of IP/H-bond acceptors calculated by M06-2X/6-311++G(2d,2p) theory level.

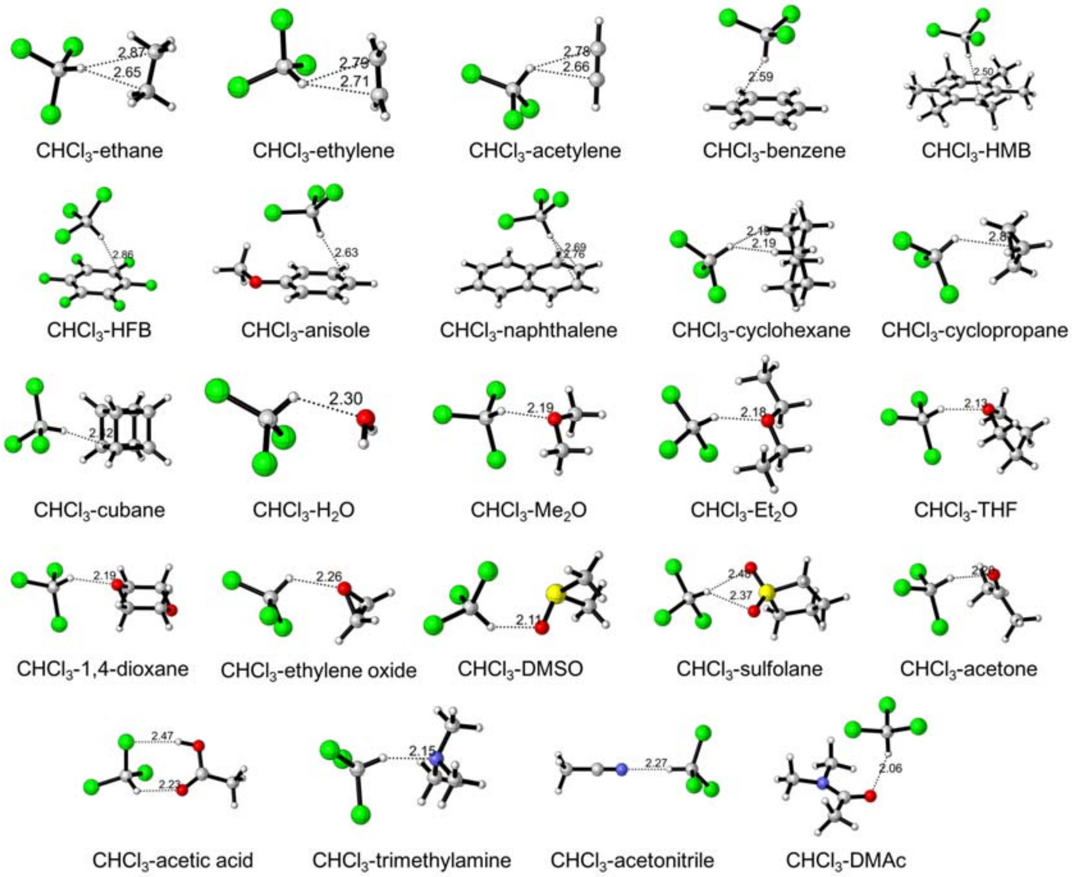

Figure S14. Optimized geometries of $CHCl_3$/H-bond acceptors calculated by M06-2X/6-311++G(2d,2p) theory level.

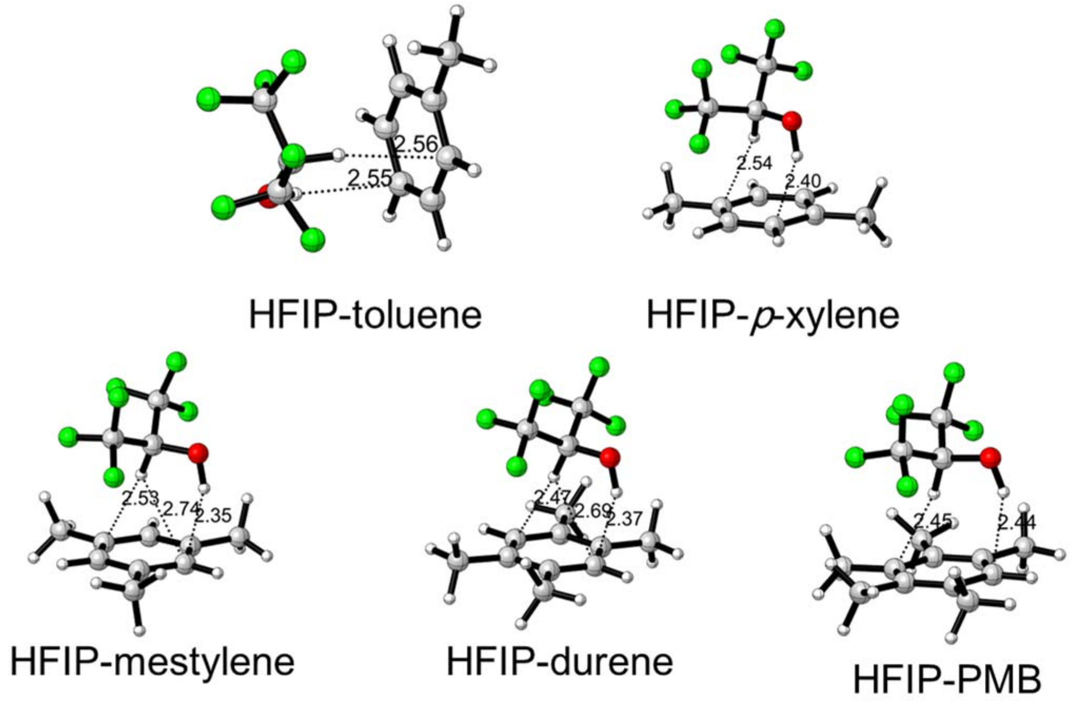

Figure S15. Optimized geometries of HFIP/$PhMe_n$ complexes (n = 1-6) calculated by M06-2X/6-311++G(2d,2p) theory level.

Cartesian coordinates of [HFIP]$_n$/PhH complexes

(1) 2HFIP-PhH

Name: 2HFIP-PhH

Charge: 0

Multiplicity: 1

E(UM062X) = -1811.888700 Ha

C	3.412369	2.693789	0.005651
C	2.984213	2.483401	-1.298837
C	1.622677	2.413899	-1.579951
C	0.689335	2.529229	-0.553805
C	1.121387	2.731417	0.752676
H	0.394652	2.822991	1.548806
H	1.290563	2.285504	-2.603783
H	-0.369302	2.459789	-0.769070
C	2.480148	2.824581	1.029338
H	2.814355	2.992106	2.045147
H	4.470009	2.757901	0.225406
H	3.707385	2.383253	-2.097627
F	0.797635	-2.567992	0.837099
F	2.082611	-1.510050	2.208221
O	1.092775	-0.691362	-1.082327
H	1.183511	0.159067	-1.536803
F	3.098533	-2.695046	-0.684184
F	3.756371	-0.829207	-1.533344
F	4.214794	-1.297780	0.519957
F	0.242479	-0.610114	1.528548
C	1.990716	-0.700532	-0.011234
H	2.259715	0.307363	0.315878
C	3.283121	-1.406583	-0.426486
C	1.281810	-1.374290	1.158597
F	-3.400501	1.939709	-0.271317
F	-4.006924	0.859855	1.493118
O	-1.609165	0.052689	-0.976073
H	-0.804674	-0.474514	-0.863633
F	-4.354351	-0.320783	-1.572821
F	-3.212699	-2.121971	-1.270282
F	-4.589597	-1.456879	0.245016
F	-1.983031	1.577337	1.304745
C	-2.557592	-0.264640	-0.014008
H	-2.168668	-0.892200	0.792135
C	-3.706445	-1.042222	-0.664257
C	-3.010790	1.046019	0.630304

(2) 3HFIP-PhH-linear

Name: 3HFIP-PhH-linear

Charge: 0

Multiplicity: 1

E(UM062X) = -2601.716997 Ha

C	-3.470215	-1.581731	2.789839
C	-2.272150	-2.224850	2.475747
C	-1.088840	-1.487499	2.383822
C	-1.110707	-0.105384	2.591708
C	-2.310792	0.539240	2.887277
H	-2.325596	1.616961	3.026806
H	-0.148190	-1.973451	2.134643
H	-0.194435	0.470338	2.491963
C	-3.488848	-0.202010	2.995864
H	-4.425131	0.298577	3.228672
H	-4.390286	-2.156087	2.862519
H	-2.257468	-3.296282	2.295119
F	-2.328986	1.083367	-2.301063
F	-4.294945	1.004491	-1.326217
O	-1.215364	-0.657452	-0.576505
H	-0.969442	-1.142201	0.245015
F	-3.085752	-1.672300	-2.605778
F	-2.772015	-2.859172	-0.791610
F	-4.624857	-1.716488	-1.041655
F	-2.567194	1.832151	-0.262595
C	-2.624268	-0.515213	-0.537550
H	-2.987206	-0.530436	0.497718
C	-3.288039	-1.700045	-1.271065
C	-2.969336	0.857270	-1.128447
F	1.540619	3.231017	1.715415
F	0.596855	4.713280	0.396505
O	0.542343	1.139704	0.169574
H	-0.204323	0.621161	-0.236784
F	3.005863	2.247371	-0.500341
F	1.805039	1.656199	-2.231925
F	2.044559	3.781359	-1.742626
F	-0.633503	3.257475	1.476286
C	0.616840	2.437308	-0.353171
H	-0.236663	2.646608	-1.009748
C	1.890764	2.549439	-1.209854
C	0.544023	3.429226	0.821573
F	3.844634	-2.882859	1.396788
F	5.011072	-1.813586	-0.124443
O	1.662742	-1.086038	0.961646
H	1.363854	-0.155758	0.829729

F	1.704361	-3.451751	-0.435996
F	1.157230	-1.772707	-1.729889
F	3.142104	-2.683645	-1.905830
F	4.284554	-0.750876	1.648208
C	2.674428	-1.299249	0.011977
H	2.900962	-0.379746	-0.543699
C	2.177464	-2.326127	-1.022375
C	3.967910	-1.706928	0.738195

(3) 3HFIP-PhH-branched

Name: 3HFIP-PhH-branched
Charge: 0
Multiplicity: 1
E(UM062X) = -2601.728991 Ha

C	0.301690	3.684676	-1.883183
C	0.643822	2.429991	-2.376652
C	-0.327170	1.438447	-2.484499
C	-1.634101	1.691655	-2.074952
C	-1.972714	2.945285	-1.576465
H	-2.988071	3.141495	-1.262425
H	-0.069372	0.472376	-2.900503
H	-2.381488	0.915396	-2.149171
C	-1.007118	3.943589	-1.490442
H	-1.273452	4.920766	-1.111868
H	1.053287	4.458139	-1.807674
H	1.660961	2.224170	-2.680044
F	-0.304707	0.501388	3.049028
F	-0.847337	2.573185	2.776555
O	0.241132	0.201224	0.392180
H	0.225374	0.292075	-0.573365
F	2.189905	1.106792	2.141359
F	2.328323	1.955749	0.173218
F	1.610667	3.162881	1.812114
F	-1.951304	1.018583	1.763851
C	0.179315	1.486007	0.950911
H	-0.255649	2.215008	0.265089
C	1.594024	1.944537	1.291956
C	-0.731988	1.399730	2.167878
F	-4.487662	-0.556998	-1.737450
F	-5.711150	-0.518662	0.039800
O	-2.231637	-1.055895	-0.325262
H	-1.571224	-0.782815	0.319709
F	-3.936363	-3.157718	-1.026906
F	-2.885001	-3.430782	0.834196
F	-4.988242	-2.963255	0.847357
F	-4.141084	0.934053	-0.225435
C	-3.466158	-1.240892	0.287515
H	-3.477367	-0.955889	1.341060
C	-3.834232	-2.722386	0.224081
C	-4.476785	-0.345919	-0.423867
F	4.594491	-3.157516	0.026980
F	5.825998	-1.409829	-0.261256
O	2.425509	-1.701028	0.584765
H	1.693917	-1.079091	0.689078
F	3.436200	-1.975234	-2.152544
F	2.132092	-0.321922	-1.703317
F	4.245375	0.013199	-1.948651
F	4.986564	-1.837894	1.680118
C	3.519857	-1.045642	0.044084
H	3.696400	-0.056638	0.474316
C	3.352020	-0.846329	-1.464061
C	4.748833	-1.886683	0.371224

(4) 4HFIP-PhH

Name: 4HFIP-PhH
Charge: 0
Multiplicity: 1
E(UM062X) = -3391.579303 Ha

C	3.980416	-3.496430	2.003628
C	2.752661	-2.974059	2.385432
C	1.631953	-3.178340	1.588263
C	1.747888	-3.887376	0.398840
C	2.977394	-4.408568	0.018758
H	3.061296	-4.959789	-0.905853
H	0.663018	-2.807340	1.902250
H	0.882831	-4.039322	-0.231248
C	4.091341	-4.220952	0.825205
H	5.048775	-4.626960	0.529657
H	4.850562	-3.331436	2.623334
H	2.672667	-2.407779	3.301488
F	3.051845	0.443983	-2.212003
F	4.709885	-0.955411	-2.108689
O	1.954865	-0.452193	0.069392
H	1.532961	-1.157187	0.580704
F	4.166983	1.334317	0.158532
F	3.911531	0.009395	1.855848
F	5.495930	-0.364130	0.425796
F	2.677591	-1.686465	-2.324302
C	3.271505	-0.837265	-0.228559
H	3.467001	-1.866562	0.065437

C	4.230063	0.048620	0.556171
C	3.440407	-0.755160	-1.738677
F	-0.724959	3.907623	-1.400734
F	1.082477	5.109101	-1.344194
O	0.511982	1.670730	-0.844975
H	1.043481	0.904159	-0.553940
F	0.094910	3.625661	1.248996
F	1.729315	2.231527	1.531240
F	2.148209	4.282029	0.975810
F	0.837863	3.584714	-2.865441
C	1.258378	2.839947	-0.682879
H	2.294557	2.718852	-1.012232
C	1.307472	3.260396	0.785788
C	0.608472	3.881424	-1.583221
F	-4.665655	0.847812	0.276615
F	-4.505661	2.453084	1.729917
O	-2.185325	1.133889	-0.576305
H	-1.255331	1.340178	-0.786497
F	-2.719382	-0.070175	2.037506
F	-0.745802	0.737296	1.667705
F	-2.137884	1.822078	2.927253
F	-4.373464	2.885250	-0.392477
C	-2.529824	1.824546	0.586355
H	-2.086311	2.821870	0.624138
C	-2.040072	1.072977	1.825949
C	-4.041411	2.003799	0.556867
F	-4.251890	-3.670476	-1.915866
F	-4.288653	-3.839131	0.245714
O	-3.178843	-1.203374	-1.837815
H	-2.946115	-0.296161	-1.594694
F	-1.522659	-3.547882	-1.336705
F	-0.849019	-1.661111	-0.516112
F	-1.728143	-3.152303	0.786529
F	-5.490490	-2.330162	-0.750142
C	-3.183432	-1.952004	-0.669534
H	-3.393177	-1.357574	0.222865
C	-1.820775	-2.602575	-0.439542
C	-4.313025	-2.968477	-0.781335

(5) 5HFIP-PhH

Name: 5HFIP-PhH

Charge: 0

Multiplicity: 1

E(UM062X) = -4181.421322 Ha

C	-2.307189	-4.223026	-3.461422
C	-2.024690	-2.878331	-3.655431
C	-0.809362	-2.354714	-3.229604
C	0.112378	-3.174914	-2.589827
C	-0.171899	-4.519741	-2.397558
H	0.549008	-5.152753	-1.902067
H	-0.568661	-1.314712	-3.418334
H	1.054072	-2.773590	-2.241976
C	-1.377656	-5.045509	-2.840747
H	-1.597990	-6.093695	-2.693574
H	-3.254444	-4.625369	-3.791661
H	-2.751554	-2.241090	-4.137213
F	-2.084007	-2.277898	2.063901
F	-2.344349	-4.346195	1.453621
O	-1.946221	-1.389518	-0.466524
H	-1.499511	-1.356952	-1.324341
F	-4.472511	-1.984310	0.698428
F	-4.329693	-2.142017	-1.459416
F	-4.422395	-3.938267	-0.251202
F	-0.507660	-3.291799	0.978260
C	-2.407516	-2.697155	-0.247691
H	-2.053061	-3.383428	-1.014175
C	-3.929274	-2.695951	-0.308337
C	-1.836364	-3.165988	1.082612
F	-2.161926	2.867706	2.724124
F	-4.062551	2.223651	3.552609
O	-1.867815	0.737224	1.235122
H	-1.883925	-0.048790	0.654823
F	-3.790629	2.759234	0.463965
F	-4.125377	0.758175	-0.296467
F	-5.360000	1.518344	1.311757
F	-2.254715	1.109095	3.985366
C	-3.125315	0.908115	1.818167
H	-3.552296	-0.037007	2.166357
C	-4.118025	1.499953	0.817954
C	-2.905898	1.790926	3.038891
F	1.379269	4.473752	-0.823492
F	-0.390773	5.683349	-1.167602
O	0.041115	2.537971	0.369589
H	-0.573158	1.899422	0.777692
F	-0.153639	2.900445	-2.528285
F	-1.716838	1.785324	-1.529940
F	-2.092963	3.815209	-2.192664
F	0.287869	5.293197	0.856261
C	-0.716725	3.511285	-0.282946
H	-1.618113	3.778046	0.273045
C	-1.172637	3.010498	-1.655047
C	0.150440	4.760121	-0.362513

F	4.896914	0.901297	-1.330279
F	3.979858	1.386003	-3.234562
O	2.689872	1.575532	0.054816
H	1.877820	2.001839	0.363261
F	2.808665	-0.952018	-1.390230
F	0.847404	-0.128427	-0.987460
F	1.677230	-0.012640	-2.985301
F	4.314405	2.942543	-1.758626
C	2.580849	1.434076	-1.321213
H	1.925639	2.177888	-1.780731
C	1.995824	0.069327	-1.679234
C	3.965647	1.658610	-1.915871
F	5.719724	-1.656975	3.237344
F	5.471357	-3.025969	1.573968
O	4.299772	0.276465	2.044172
H	3.825162	0.869902	1.446301
F	2.930259	-2.060920	3.165331
F	1.913907	-0.932145	1.618461
F	2.809812	-2.847918	1.145055
F	6.585783	-1.182982	1.310750
C	4.258904	-0.994052	1.482221
H	4.279354	-0.979311	0.391110
C	2.974551	-1.727208	1.871596
C	5.519955	-1.725218	1.919502

Cartesian coordinates of optimized geometries of HFIP/H-bond acceptors

(1) HFIP-acetone

Name: HFIP-acetone
Charge: 0
Multiplicity: 1
E(UM062X) = -789.824628917 Ha

C	3.141491	0.102364	1.303080
C	3.195097	-0.538233	-0.056456
C	4.400336	-0.243766	-0.905300
O	2.309733	-1.262198	-0.464108
H	3.333146	1.171956	1.216983
H	3.936250	-0.320095	1.920592
H	2.178093	-0.063998	1.775884
H	5.315349	-0.385690	-0.330604
H	4.367472	0.806138	-1.202663
H	4.401906	-0.873454	-1.789173
C	-0.781233	-0.328450	-0.218969
C	-0.523642	1.173051	-0.089355
C	-2.259343	-0.676839	-0.083655
O	-0.095614	-1.018532	0.765758
H	0.784728	-1.248692	0.413289
F	-0.990306	1.678006	1.047071
F	-1.051548	1.861108	-1.101309
F	0.802131	1.388646	-0.110793
F	-2.715151	-0.452218	1.143338
F	-3.004191	0.030135	-0.938336
F	-2.439268	-1.967862	-0.360051
H	-0.481154	-0.609423	-1.232848

(2) HFIP-acetonitrile

Name: HFIP-acetonitrile
Charge: 0
Multiplicity: 1
E(UM062X) = -789.824704832 Ha

C	5.160999	-0.302324	0.464549
C	3.799898	-0.549958	0.005885
N	2.732275	-0.742794	-0.355496
H	5.143614	0.405572	1.289593
H	5.749541	0.110028	-0.351353
H	5.609561	-1.234860	0.797895
C	-0.449699	-0.252074	-0.040851
C	-1.806916	-0.904346	0.193443
C	-0.519147	1.274690	-0.006192
O	0.016078	-0.683334	-1.271028
H	0.978036	-0.768636	-1.203569
F	-2.651367	-0.680788	-0.806375
F	-2.371091	-0.458854	1.319967
F	-1.652071	-2.223694	0.313294
F	-1.412134	1.766089	-0.858656
F	-0.817573	1.727102	1.214912
F	0.677549	1.771383	-0.335102
H	0.194065	-0.552941	0.790764

(3) HFIP-ethane

Name: HFIP-ethane
Charge: 0
Multiplicity: 1
E(UM062X) = -77.3199190459 Ha

C	-3.475221	-1.491482	-0.266412
C	-3.573402	-0.300223	-0.260418
H	-3.394302	-2.554037	-0.270328
H	-3.660946	0.761972	-0.255074
F	0.805208	1.764382	0.979654
F	0.557471	1.794456	-1.161490
O	-0.679614	-0.669463	1.096284
H	-1.629150	-0.725494	0.919340
F	2.027576	-0.719141	1.009892
F	1.154514	-2.213400	-0.269404
F	2.051925	-0.453865	-1.129129
F	-1.168976	1.820130	0.124615
C	-0.036476	-0.215765	-0.045730
H	-0.563502	-0.479175	-0.968942
C	1.327872	-0.902336	-0.102651
C	0.063121	1.313036	-0.021873

(4) HFIP-ethylene

Name: HFIP-ethylene
Charge: 0
Multiplicity: 1
E(UM062X) = -78.5682275383 Ha

C	3.282854	-0.954923	0.859681
C	3.747141	-0.723953	-0.360395
H	2.912274	-0.148088	1.481715
H	3.275842	-1.950565	1.286578
H	3.762010	0.275320	-0.778566
H	4.138542	-1.523003	-0.978372
F	-0.617934	1.734896	-1.037423
F	-0.420012	1.839422	1.107189
O	0.524280	-0.920189	-0.941842
H	1.469161	-0.980446	-0.738679
F	-2.137500	-0.578189	-1.103046
F	-1.634347	-2.056598	0.376426
F	-2.304837	-0.103712	0.992669
F	1.320850	1.528789	-0.121073
C	-0.121151	-0.294471	0.112988
H	0.277234	-0.576371	1.094417
C	-1.577207	-0.757594	0.085122
C	0.020813	1.228140	0.008432

(5) HFIP-acetylene

Name: HFIP-acetylene
Charge: 0
Multiplicity: 1
E(UM062X) = -789.768767615 Ha

C	-3.464303	-0.858317	0.903483
C	-3.662204	-0.484093	-0.560519
H	-4.256281	-1.502509	1.264902
H	-3.435677	0.007169	1.546983
H	-3.626131	-1.342239	-1.213912
H	-4.613367	0.009982	-0.726891
H	-2.531489	-1.394285	1.023442
H	-2.889118	0.214950	-0.869127
F	2.368452	-1.156627	-0.626647
F	1.700190	-1.397589	1.273480
O	-0.102978	-0.000886	-1.461938
H	-0.995265	-0.210652	-1.572591
F	1.331059	2.118192	-0.551150
F	-0.602816	2.166762	-0.159897
F	0.620597	1.699936	1.392110
F	0.720463	-2.265365	-0.282827
C	0.182501	0.004183	-0.164038
H	-0.637529	-0.318073	0.509046
C	0.413252	1.531376	0.139386
C	1.291949	-1.183989	0.069693

(6) HFIP-cubane

Name: HFIP-cubane
Charge: 0
Multiplicity: 1
E(UM062X) = -789.825129628 Ha

C	-1.210886	-0.061514	-0.311192
C	-2.128274	-1.196318	0.126370
C	-1.735428	1.315811	0.090858
O	-1.083432	-0.149058	-1.693391
H	-0.202450	0.143390	-1.943436
F	-3.271173	-1.206613	-0.546250
F	-2.408108	-1.110047	1.428145
F	-1.513759	-2.361851	-0.083426
F	-2.983003	1.526769	-0.310457
F	-1.684265	1.486412	1.412558
F	-0.959617	2.252256	-0.466838

H	-0.268730	-0.204179	0.222002
C	1.993493	0.704823	0.840970
C	3.554939	0.696960	0.830428
C	1.985321	0.712348	-0.721813
C	3.545482	0.700117	-0.730229
C	3.549650	-0.863846	0.825245
C	3.538884	-0.859518	-0.735413
C	1.977860	-0.858501	-0.728136
C	1.989567	-0.859895	0.835868
H	1.373469	1.334359	1.471197
H	4.184469	1.320780	1.454307
H	1.379650	1.376767	-1.333595
H	4.165874	1.326095	-1.361177
H	4.174034	-1.495611	1.446254
H	4.155481	-1.485963	-1.369585
H	1.351687	-1.497138	-1.344865
H	1.369657	-1.491168	1.464710

(7) HFIP-cyclohexane

Name: HFIP-cyclohexane
Charge: 0
Multiplicity: 1
E(UM062X) = -789.825507380 Ha

C	1.125001	-0.010082	-0.225832
C	1.614534	1.342587	0.288690
C	2.118871	-1.136408	0.028649
O	0.916197	0.037012	-1.598805
H	0.068557	0.456939	-1.775037
F	2.816615	1.663231	-0.171366
F	1.649242	1.375416	1.619940
F	0.753428	2.285949	-0.117069
F	3.219148	-1.008689	-0.700311
F	2.469877	-1.179282	1.314731
F	1.550125	-2.302504	-0.280391
H	0.222583	-0.255703	0.342482
C	-2.524586	-1.287137	0.775545
C	-2.172712	-1.117977	-0.703232
C	-2.284255	0.348134	-1.128187
C	-3.916616	-0.729962	1.076203
C	-4.030951	0.732970	0.646357
C	-3.677974	0.904992	-0.831152
H	-1.792119	-0.752153	1.390873
H	-2.870001	-1.708881	-1.303878
H	-5.036955	1.107015	0.838842
H	-3.733898	1.955844	-1.116431
H	-4.143026	-0.831962	2.137953
H	-2.463605	-2.338050	1.059504
H	-1.177271	-1.514418	-0.915245
H	-2.061888	0.456627	-2.193412
H	-1.557669	0.953772	-0.569920
H	-4.660574	-1.321795	0.534356
H	-3.347612	1.338496	1.250043
H	-4.409894	0.368375	-1.441729

(8) HFIP-cyclopropane

Name: HFIP-cyclopropane
Charge: 0
Multiplicity: 1
E(UM062X) = -789.824981196 Ha

C	-0.336975	-0.143719	0.006914
C	-1.534857	-1.080163	0.119995
C	-0.735053	1.331948	0.033113
O	0.311289	-0.442133	-1.182895
H	1.260386	-0.334477	-1.049269
F	-2.310564	-1.030681	-0.954685
F	-2.278626	-0.783986	1.187536
F	-1.101532	-2.333977	0.259984
F	-1.635097	1.633191	-0.894134
F	-1.221161	1.687387	1.222867
F	0.354990	2.074377	-0.194704
H	0.282496	-0.318051	0.891580
C	3.160849	-1.074809	-0.060896
C	3.138893	0.438826	0.017384
C	4.364008	-0.323997	0.419639
H	3.175224	1.000794	-0.904472
H	4.597693	-0.373496	1.471427
H	3.215354	-1.534801	-1.036699
H	2.588591	-1.631019	0.667159
H	2.557463	0.901740	0.800671
H	5.219211	-0.278940	-0.235917

(9) HFIP-1,4-dioxane

Name: HFIP-1,4-dioxane
Charge: 0
Multiplicity: 1

E(UM062X) = -789.824694610 Ha

O	1.981207	-0.317674	-1.079855
C	2.564199	-1.503258	-0.553994
C	2.837859	0.806171	-0.912279
H	3.465280	-1.745416	-1.128228
H	1.831405	-2.300664	-0.681711
H	2.307070	1.671623	-1.304858
H	3.754381	0.644979	-1.490619
C	2.918560	-1.305471	0.906615
C	3.173015	0.979677	0.554003
H	3.443887	-2.176745	1.296509
H	2.002818	-1.154187	1.495058
H	2.256375	1.205575	1.115576
H	3.883915	1.792784	0.695651
O	3.773918	-0.194514	1.062331
C	-1.028611	-0.167714	-0.150729
C	-2.284043	-0.976634	0.168490
C	-1.206987	1.333089	0.101402
O	-0.702439	-0.406445	-1.472420
H	0.265050	-0.324416	-1.556227
F	-3.265786	-0.744397	-0.691790
F	-2.734451	-0.707233	1.396320
F	-1.993856	-2.278241	0.120805
F	-2.259703	1.842703	-0.524080
F	-1.320473	1.604865	1.402579
F	-0.117069	1.975750	-0.343010
H	-0.253578	-0.500638	0.549940

(10) HFIP-DMAc

Name: HFIP-DMAc
Charge: 0
Multiplicity: 1
E(UM062X) = -789.824073915 Ha

C	-1.131598	-0.325963	0.106921
C	-2.635931	-0.377546	0.350399
C	-0.694139	1.006168	-0.503026
O	-0.798457	-1.373918	-0.732673
H	0.044053	-1.739748	-0.409042
F	-3.326383	-0.402171	-0.783879
F	-3.048882	0.676290	1.062689
F	-2.937985	-1.476642	1.040149
F	-1.356216	1.312740	-1.611390
F	-0.833301	2.016108	0.360273
F	0.611379	0.942698	-0.818856
H	-0.644863	-0.394550	1.083804
C	2.444646	-0.993285	0.000000
O	1.559744	-1.655993	0.543209
N	3.022831	0.051489	0.631436
C	2.456793	0.487470	1.897804
C	3.855481	1.026182	-0.050181
C	2.901509	-1.326874	-1.399279
H	1.651817	1.209169	1.730721
H	3.236505	0.961691	2.490071
H	2.063309	-0.370201	2.431534
H	4.583926	1.423633	0.654221
H	3.251522	1.853386	-0.432452
H	4.394110	0.571905	-0.873843
H	3.976380	-1.493974	-1.443235
H	2.650838	-0.513322	-2.080186
H	2.384223	-2.227240	-1.712637

(11) HFIP-DMSO

Name: HFIP-DMSO
Charge: 0
Multiplicity: 1
E(UM062X) = -789.824117481 Ha

C	-1.067481	-0.308869	-0.292771
C	-0.950075	1.207542	-0.146504
C	-2.474781	-0.813653	0.003817
O	-0.198051	-0.933442	0.582263
H	0.651245	-1.078603	0.107014
F	-1.265372	1.629616	1.074836
F	-1.703585	1.866303	-1.024254
F	0.327274	1.564823	-0.371232
F	-2.815404	-0.627337	1.275243
F	-3.383591	-0.200701	-0.760679
F	-2.543796	-2.119010	-0.253170
H	-0.861529	-0.536761	-1.342871
S	3.014332	0.093647	-0.360208
C	4.600337	-0.746601	-0.204501
C	2.716750	0.344587	1.400788
H	4.459321	-1.625354	0.420327
H	5.329097	-0.061073	0.222487
H	4.898680	-1.038993	-1.207239
H	2.730394	-0.627701	1.889150
H	1.736864	0.802669	1.498274
H	3.488191	1.002425	1.795159

O	2.054324	-0.998589	-0.788593

(12) HFIP-ethylene oxide

Name: HFIP-ethylene oxide
Charge: 0
Multiplicity: 1
E(UM062X) = -789.825308802 Ha

C	-0.568984	-0.183153	-0.536119
C	-0.372777	1.251246	-0.046913
C	-1.852843	-0.811303	-0.007214
O	0.476970	-0.984615	-0.113555
H	1.306820	-0.734378	-0.565326
F	-0.357334	1.342304	1.279685
F	-1.306401	2.076105	-0.514043
F	0.814707	1.689333	-0.492529
F	-1.829420	-0.966407	1.311535
F	-2.914229	-0.064284	-0.319338
F	-2.022367	-2.011287	-0.560326
H	-0.672435	-0.130561	-1.624865
O	3.016771	-0.408506	-0.760785
C	3.401599	0.208632	0.468418
C	3.647228	-1.215821	0.235745
H	2.574628	0.499269	1.103093
H	4.215947	0.913148	0.375246
H	2.994717	-1.940567	0.705252
H	4.640459	-1.551421	-0.027031

(13) HFIP-H2O

Name: HFIP-H2O
Charge: 0
Multiplicity: 1
E(UM062X) = -789.824936173 Ha

O	2.898479	1.612691	-0.397533
H	3.554422	2.311157	-0.437855
H	3.380258	0.786740	-0.305610
C	0.060679	0.397427	-0.117934
C	-1.462282	0.456180	-0.110453
C	0.587271	-1.031863	0.014193
O	0.511723	1.176954	0.934559
H	1.384982	1.517483	0.691161
F	-1.970231	0.125733	1.070412
F	-1.979718	-0.363245	-1.029673
F	-1.860940	1.694809	-0.398846
F	0.113896	-1.660865	1.081095
F	0.313089	-1.764940	-1.063617
F	1.927822	-0.989798	0.133593
H	0.389458	0.761749	-1.095410

(14) HFIP-HFB

Name: HFIP-HFB
Charge: 0
Multiplicity: 1
E(UM062X) = -789.825644150 Ha

C	1.480584	0.099423	0.117688
C	1.849092	-1.340571	-0.219465
C	2.511213	1.108570	-0.384409
O	1.350954	0.160522	1.500777
H	1.146618	1.060815	1.773517
F	2.943676	-1.742288	0.407577
F	2.036782	-1.477799	-1.531879
F	0.847189	-2.146904	0.138608
F	3.743213	0.837935	0.021411
F	2.507169	1.190314	-1.710632
F	2.175701	2.315118	0.101751
H	0.547331	0.317160	-0.416088
C	-2.311724	0.817185	-0.977974
C	-1.959854	1.464913	0.195495
C	-1.608074	0.726797	1.312651
C	-2.308897	-0.566857	-1.029220
F	-2.652029	1.520454	-2.046086
F	-1.951626	2.788454	0.246680
F	-1.238592	1.352663	2.424100
F	-2.646705	-1.186303	-2.147987
C	-1.958224	-1.303652	0.091333
C	-1.606292	-0.657134	1.264505
F	-1.256591	-1.356638	2.326831
F	-1.955801	-2.623251	0.037705

(15) HFIP-HMB

Name: HFIP-HMB
Charge: 0

Multiplicity: 1

E(UM062X) = -789.824454350 Ha

C	1.308786	0.044071	-0.445345
C	2.171193	1.227222	-0.021390
C	1.897791	-1.306448	-0.032790
O	1.151942	0.105441	-1.826667
H	0.242450	-0.145328	-2.026966
F	3.306193	1.307757	-0.704095
F	2.471273	1.159654	1.278772
F	1.493498	2.361070	-0.220350
F	3.170854	-1.452241	-0.386002
F	1.810748	-1.488275	1.287843
F	1.199427	-2.286363	-0.616385
H	0.364292	0.144784	0.091469
C	-1.751405	-1.295603	0.597604
C	-1.913084	-1.020038	-0.768123
C	-2.019667	0.306963	-1.203993
C	-1.623931	-0.244713	1.513222
C	-1.735931	-2.725196	1.081680
C	-1.952946	-2.157273	-1.762628
C	-2.323604	0.618611	-2.652942
C	-1.329469	-0.538860	2.964526
C	-1.718836	1.083058	1.074039
C	-1.877617	1.358905	-0.288528
C	-1.869490	2.785126	-0.781348
C	-1.650779	2.216475	2.068450
H	-2.876217	3.213661	-0.801915
H	-2.031707	1.912240	3.040658
H	-2.336864	-3.365389	0.439567
H	-2.144716	-2.802836	2.086727
H	-0.721590	-3.134694	1.102273
H	-1.582948	-1.851741	-2.740097
H	-2.969016	-2.537629	-1.903584
H	-1.331621	-2.986714	-1.432368
H	-2.852772	-0.200520	-3.133270
H	-1.424153	0.825710	-3.243369
H	-2.956002	1.501337	-2.729176
H	-0.780891	-1.471581	3.073503
H	-2.245098	-0.618769	3.558079
H	-0.717679	0.244885	3.406765
H	-1.461951	2.849733	-1.788404
H	-1.252466	3.416999	-0.146775
H	-2.246435	3.064812	1.738250
H	-0.624159	2.569279	2.209310

(16) HFIP-acetic acid

Name: HFIP-acetic acid

Charge: 0

Multiplicity: 1

E(UM062X) = -789.824543281 Ha

C	-0.634261	-0.367015	-0.120584
C	-2.112106	-0.537052	0.211577
C	-0.231691	1.109317	-0.166871
O	-0.407323	-0.976221	-1.344574
H	0.480645	-1.355568	-1.324524
F	-2.899224	-0.163393	-0.789575
F	-2.445101	0.179083	1.290592
F	-2.366302	-1.817754	0.477943
F	-1.055545	1.849956	-0.898560
F	-0.172574	1.639408	1.057940
F	0.994058	1.225651	-0.704451
H	-0.068013	-0.819002	0.696473
O	2.235940	-0.391071	1.383795
C	2.777129	-0.678656	0.184416
C	4.007306	0.095818	-0.191473
O	2.252148	-1.505609	-0.507940
H	4.767530	0.008294	0.584110
H	3.740088	1.147915	-0.298336
H	4.392369	-0.277394	-1.133290
H	2.705202	0.327931	1.817928

(17) HFIP- naphthalene

Name: HFIP-naphthalene

Charge: 0

Multiplicity: 1

E(UM062X) = -789.824820273 Ha

C	0.879283	2.654166	0.626822
C	0.956916	2.608907	-0.785381
C	1.624199	1.588490	-1.406515
C	2.228549	0.549569	-0.652885
C	2.154504	0.599160	0.763768
C	1.473201	1.680406	1.379138
C	2.892505	-0.537606	-1.270167
C	3.451296	-1.528907	-0.515472
C	3.375289	-1.482205	0.894232
C	2.743773	-0.444374	1.518222
H	0.347000	3.465095	1.105863

H	0.493971	3.394068	-1.369614
H	1.706401	1.559948	-2.487949
H	1.417297	1.711370	2.460664
H	2.942060	-0.572496	-2.351745
H	3.951121	-2.359897	-0.995382
H	3.818379	-2.277679	1.478753
H	2.680117	-0.405960	2.598912
C	-1.213735	-0.145254	-0.347142
C	-1.256281	-1.636361	-0.024166
C	-2.404652	0.630772	0.221239
O	-1.173396	-0.020402	-1.731832
H	-0.605903	0.728556	-1.947496
F	-2.230171	-2.266606	-0.670092
F	-1.426180	-1.840408	1.284252
F	-0.099732	-2.197139	-0.376653
F	-3.575573	0.105574	-0.122759
F	-2.353322	0.697150	1.552508
F	-2.367192	1.884294	-0.244055
H	-0.322825	0.253851	0.143685

(18) HFIP-Et2O

Name: HFIP-Et2O
Charge: 0
Multiplicity: 1
E(UM062X) = -789.823947299 Ha

O	-2.603891	0.104507	-0.095857
C	-3.496207	-0.991567	0.037658
C	-3.265381	1.358838	-0.160635
H	-4.185184	-0.991291	-0.814582
C	-2.696494	-2.271019	0.088796
H	-4.090534	-0.860787	0.949361
H	-3.879855	1.488049	0.737813
H	-3.935188	1.359833	-1.028002
C	-2.230525	2.452765	-0.270598
H	-3.365781	-3.124729	0.193070
H	-2.008826	-2.265537	0.934676
H	-2.115553	-2.395884	-0.824511
H	-1.575533	2.458323	0.600328
H	-2.719501	3.424046	-0.341953
H	-1.617516	2.312100	-1.161446
C	0.736131	0.015936	-0.086799
C	1.454432	-1.336259	-0.145468
C	1.708620	1.192284	0.028479
O	-0.114434	0.041979	1.004187
H	-1.039873	0.038913	0.688179
F	2.222482	-1.549813	0.916798
F	2.215183	-1.444851	-1.236730
F	0.545296	-2.313755	-0.195214
F	2.369182	1.189341	1.179259
F	2.601558	1.195258	-0.963340
F	1.024558	2.337965	-0.045905
H	0.212146	0.131902	-1.041987

(19) HFIP-Me2O

Name: HFIP-Me2O
Charge: 0
Multiplicity: 1
E(UM062X) = -789.824719090 Ha

O	2.796689	-0.325596	0.044976
C	3.413668	-1.576875	0.261585
C	3.714011	0.747874	0.035037
H	4.153027	-1.784960	-0.517755
H	2.633741	-2.335388	0.231251
H	3.906551	-1.597690	1.238019
H	4.455042	0.614799	-0.758997
H	4.229256	0.817868	0.997584
H	3.148552	1.659252	-0.141678
C	-0.391819	-0.165124	0.063686
C	-1.662042	-1.012498	0.078986
C	-0.679236	1.339583	0.050443
O	0.336640	-0.527207	-1.055181
H	1.281443	-0.400165	-0.847981
F	-2.334118	-0.934484	-1.062092
F	-2.480023	-0.644927	1.068280
F	-1.337564	-2.291616	0.277503
F	-1.478579	1.703595	-0.944854
F	-1.224903	1.751451	1.195419
F	0.480017	1.995808	-0.096542
H	0.134804	-0.367510	1.003346

(20) HFIP- anisole

Name: HFIP- anisole
Charge: 0
Multiplicity: 1
E(UM062X) = -789.824423226 Ha

C	1.127371	0.229425	0.297377
C	1.107180	-1.264600	0.638063
C	2.144382	0.568277	-0.790254
O	1.448155	0.981925	1.421458
H	0.626441	1.306579	1.807150
F	2.321562	-1.766688	0.837138
F	0.522835	-1.970124	-0.335869
F	0.398806	-1.461629	1.752828
F	3.395927	0.481070	-0.358158
F	2.009991	-0.241712	-1.842776
F	1.942621	1.818522	-1.209631
H	0.142734	0.471931	-0.109611
C	-2.148616	1.360524	-1.024431
C	-1.743091	2.381517	-0.187763
C	-1.643674	2.172794	1.188415
C	-1.945459	0.922595	1.704110
C	-2.344152	-0.120787	0.871157
C	-2.453195	0.100594	-0.498604
H	-2.232096	1.504364	-2.093367
H	-1.504505	3.350217	-0.606917
H	-1.349182	2.982798	1.843243
H	-1.876556	0.741962	2.770036
H	-2.560417	-1.088333	1.299097
O	-2.828380	-0.837619	-1.393844
C	-3.101505	-2.138278	-0.913915
H	-3.943531	-2.130660	-0.217059
H	-2.223864	-2.567479	-0.424622
H	-3.358339	-2.733136	-1.785583

(21) HFIP-sulfolane

Name: HFIP-sulfolane
Charge: 0
Multiplicity: 1
E(UM062X) = -789.824950946 Ha

C	-1.611612	-0.312411	-0.135807
C	-3.054559	-0.701073	0.157068
C	-1.389916	1.195953	-0.025020
O	-1.317987	-0.750391	-1.417754
H	-0.364548	-0.914923	-1.467644
F	-3.889722	-0.294334	-0.792543
F	-3.466028	-0.182789	1.318351
F	-3.152150	-2.027697	0.246901
F	-2.251580	1.902084	-0.749723
F	-1.457754	1.621930	1.234521
F	-0.156432	1.489820	-0.478897
H	-0.989948	-0.771774	0.637304
S	2.053843	-0.491731	0.156289
C	2.733056	1.147527	-0.203198
C	3.633944	-1.314840	0.427122
H	2.201750	1.858930	0.420067
H	2.513740	1.329069	-1.252450
H	3.546425	-2.318209	0.021783
H	3.782452	-1.345051	1.504604
O	1.461779	-1.040393	-1.057589
O	1.262769	-0.475969	1.367560
C	4.641619	-0.409772	-0.273316
C	4.230870	1.028143	0.078721
H	4.587247	-0.562890	-1.351882
H	5.656708	-0.631614	0.047655
H	4.791495	1.762591	-0.494720
H	4.423273	1.213319	1.136016

(22) HFIP-THF

Name: HFIP-THF
Charge: 0
Multiplicity: 1
E(UM062X) = -789.824583843 Ha

O	-2.257893	-0.230874	-0.501224
C	-3.146618	-1.355770	-0.478390
C	-2.933614	0.947805	-0.029894
H	-2.591177	-2.220049	-0.115044
H	-3.488461	-1.555077	-1.497606
H	-2.759593	1.751706	-0.743433
H	-2.499951	1.231489	0.932095
C	-4.299123	-0.937591	0.420818
C	-4.399363	0.554762	0.106075
H	-4.034025	-1.088593	1.468575
H	-5.214053	-1.487980	0.210671
H	-4.922870	0.705290	-0.839415
H	-4.910169	1.127718	0.877275
C	1.044570	-0.196392	-0.232608
C	2.322076	-1.009523	-0.034198
C	1.262947	1.306686	-0.028633
O	0.103632	-0.673751	0.659580
H	-0.788868	-0.515595	0.280234
F	2.720872	-1.020577	1.231540
F	3.322636	-0.531889	-0.779153

F	2.107872	-2.271952	-0.407404
F	1.816145	1.593858	1.144192
F	2.023690	1.835123	-0.987955
F	0.073996	1.923353	-0.078756
H	0.751118	-0.323004	-1.281385

(23) HFIP-trimethylamine

Name: HFIP-trimethylamine
Charge: 0
Multiplicity: 1
E(UM062X) = -789.823192802 Ha

N	2.733716	-0.325682	0.036084
C	3.374870	-1.628319	-0.125265
C	2.883028	0.156694	1.406078
C	3.285271	0.640426	-0.911650
H	2.928436	-2.342085	0.564998
H	4.453083	-1.571290	0.070284
H	3.219089	-1.985699	-1.141708
H	2.368193	1.109595	1.514230
H	3.939789	0.293692	1.669210
H	2.445994	-0.563359	2.096884
H	4.361897	0.782714	-0.754322
H	2.778549	1.595453	-0.791447
H	3.120696	0.285053	-1.927482
C	-0.662418	-0.207132	0.192888
C	-1.981511	-0.960267	0.066559
C	-0.833922	1.300452	0.003414
O	0.208782	-0.716624	-0.743882
H	1.151765	-0.553817	-0.432522
F	-2.470146	-0.916162	-1.166880
F	-2.905926	-0.464872	0.895894
F	-1.795089	-2.241058	0.390077
F	-1.400262	1.616776	-1.154049
F	-1.548642	1.854237	0.984337
F	0.382657	1.872921	0.029450
H	-0.328996	-0.345194	1.228729

(24) HFIP-Tol

Name: HFIP-Tol
Charge: 0
Multiplicity: 1
E(UM062X) = -789.824595284 Ha

C	0.872901	0.168622	-0.407348
C	1.812917	1.029259	0.425460
C	1.126187	-1.326495	-0.210296
O	1.047715	0.524295	-1.740816
H	0.187749	0.496684	-2.170279
F	3.061888	0.998578	-0.021746
F	1.816272	0.630781	1.701226
F	1.397508	2.296878	0.395785
F	2.413043	-1.648660	-0.304045
F	0.683982	-1.733047	0.984313
F	0.465025	-2.019261	-1.140747
H	-0.134637	0.369218	-0.042452
C	-2.406769	-0.994842	-0.245786
C	-2.313562	-0.442003	-1.517851
C	-2.278381	0.939031	-1.677967
C	-2.470111	-0.184230	0.885962
C	-2.522294	-0.788884	2.262527
C	-2.434852	1.199987	0.711534
C	-2.333990	1.759627	-0.555783
H	-1.513130	-0.991680	2.625364
H	-3.066075	-1.731758	2.255993
H	-3.003332	-0.115939	2.969711
H	-2.422419	-2.070900	-0.129597
H	-2.272265	-1.091429	-2.381972
H	-2.225073	1.374382	-2.667152
H	-2.480132	1.844870	1.579760
H	-2.304142	2.834337	-0.668631

Cartesian coordinates of optimized geometries of IP/H-bond acceptors

(1) IP-acetone

Name: IP-acetone
Charge: 0
Multiplicity: 1
E(UM062X) = -194.330724294 Ha

C	-1.649321	-1.451473	-0.073525
C	-1.987177	0.007959	0.095544
C	-3.228914	0.504027	-0.600423
O	-1.302070	0.753283	0.758903
H	-1.680514	-1.723679	-1.129053
H	-2.411471	-2.048272	0.431344
H	-0.671917	-1.669996	0.348841

H	-4.078717	-0.133527	-0.355389
H	-3.082718	0.441485	-1.680091
H	-3.432073	1.531796	-0.316761
C	1.695968	-0.009544	-0.212359
C	2.946810	-0.788910	-0.568936
C	1.923232	1.494151	-0.256773
O	1.306654	-0.424781	1.087240
H	0.490190	0.044005	1.302133
H	3.740524	-0.548885	0.139066
H	3.285689	-0.537770	-1.573615
H	2.755269	-1.859671	-0.518366
H	2.714801	1.765470	0.442497
H	2.214641	1.814517	-1.257738
H	1.013927	2.025452	0.025221
H	0.902115	-0.266195	-0.928401

(2) IP-AN

Name: IP-AN
Charge: 0
Multiplicity: 1
E(UM062X) = -194.330673478 Ha

C	4.171158	0.175055	0.225292
C	2.760651	-0.133212	0.019053
N	1.654021	-0.375613	-0.141744
H	4.265276	1.109369	0.773254
H	4.668814	0.271518	-0.736535
H	4.640788	-0.623762	0.793911
C	-1.785598	0.001789	0.276963
C	-3.264705	-0.271942	0.470331
C	-1.538145	1.376858	-0.328830
O	-1.284516	-1.019830	-0.568874
H	-0.325594	-0.932770	-0.592878
H	-3.771112	-0.238485	-0.495045
H	-3.714186	0.472424	1.127219
H	-3.414018	-1.260358	0.901462
H	-2.041064	1.446924	-1.294097
H	-1.917060	2.165545	0.322584
H	-0.471393	1.539165	-0.483635
H	-1.282643	-0.052922	1.250106

(3) IP-ethane

Name: IP-ethane
Charge: 0
Multiplicity: 1
E(UM062X) = -77.3199285959 Ha

C	-2.488011	-0.677888	-0.405990
C	-2.890086	0.382590	-0.030868
H	-2.119681	-1.622331	-0.729099
H	-3.260747	1.323817	0.296554
O	0.350809	-0.739200	0.910178
H	-0.551924	-0.432564	1.033578
C	0.905866	-0.043475	-0.199226
H	0.335114	-0.288594	-1.103314
C	2.326607	-0.547122	-0.358936
C	0.851882	1.459885	0.021739
H	1.406877	1.720463	0.923467
H	1.285001	1.993209	-0.824748
H	-0.180165	1.793232	0.140431
H	2.903188	-0.309498	0.535409
H	2.805367	-0.080867	-1.219337
H	2.332945	-1.627212	-0.494679

(4) IP-ethylene

Name: IP-ethylene
Charge: 0
Multiplicity: 1
E(UM062X) = -78.5682549886 Ha

C	2.368637	-0.185379	0.812203
C	2.897493	0.063665	-0.375917
H	1.950855	0.605869	1.423399
H	2.332830	-1.188499	1.219582
H	2.933176	1.067354	-0.781942
H	3.319662	-0.725788	-0.985830
H	-1.389178	1.592836	-1.138592
H	-1.381333	2.064229	0.572173
O	-0.459355	-0.891638	-0.754539
H	0.482576	-0.687594	-0.774466
H	-3.003230	-0.320872	-0.673513
H	-2.599481	-1.521244	0.560736
H	-3.053374	0.124492	1.042661
H	0.138289	1.685502	-0.250483
C	-1.069406	-0.049875	0.211390
H	-0.595788	-0.214802	1.189182
C	-2.523422	-0.467291	0.295689

C	-0.916663	1.414150	-0.171131

(5) IP-acetylene

Name: IP-acetylene
Charge: 0
Multiplicity: 1
E(UM062X) = -194.330915198 Ha

C	2.480291	-0.811082	-0.341707
C	2.472044	0.675510	0.002283
H	3.392081	-1.288255	0.015008
H	2.427114	-0.963052	-1.419391
H	2.514349	0.831964	1.081348
H	3.328199	1.190418	-0.431808
H	1.633713	-1.322990	0.114914
H	1.575115	1.166795	-0.379686
O	-0.497106	-0.227754	1.163057
H	0.223386	0.395327	1.285893
C	-1.036245	-0.045918	-0.141375
H	-0.259372	-0.258355	-0.886234
C	-1.533234	1.379093	-0.328643
C	-2.149433	-1.063654	-0.290437
H	-2.924005	-0.872941	0.452722
H	-2.593185	-1.004142	-1.283516
H	-1.764618	-2.070203	-0.136458
H	-2.311945	1.597136	0.402609
H	-1.941654	1.519452	-1.329561
H	-0.722868	2.097183	-0.191018

(6) IP-cubane

Name: IP-cubane
Charge: 0
Multiplicity: 1
E(UM062X) = -194.330796824 Ha

C	2.436890	-0.047382	-0.082839
C	3.573919	-0.693995	-0.849332
C	2.638052	1.452123	0.075695
O	2.387137	-0.694925	1.184278
H	1.603927	-0.391899	1.649430
H	4.511610	-0.538179	-0.315104
H	3.661971	-0.260099	-1.844716
H	3.406054	-1.765213	-0.944390
H	3.570384	1.645029	0.607107
H	2.682183	1.940144	-0.898599
H	1.818884	1.899728	0.639872
H	1.498359	-0.229474	-0.618530
C	-0.940065	0.955313	-0.635363
C	-2.495178	0.964604	-0.481191
C	-0.798535	0.491844	0.849022
C	-2.351654	0.498906	1.001535
C	-2.551139	-0.526116	-0.941374
C	-2.405473	-0.991303	0.541712
C	-0.850821	-1.003010	0.387872
C	-0.998367	-0.536467	-1.095784
H	-0.349047	1.734339	-1.105889
H	-3.152763	1.753624	-0.828627
H	-0.113017	0.919618	1.575603
H	-2.892154	0.914572	1.844582
H	-3.253154	-0.933886	-1.660011
H	-2.991610	-1.771112	1.014895
H	-0.187085	-1.790816	0.728539
H	-0.457410	-0.954090	-1.938106

(7) IP-cyclohexane

Name: IP-cyclohexane
Charge: 0
Multiplicity: 1
E(UM062X) = -194.330925445 Ha

C	2.287195	0.059898	0.200451
C	3.449628	-0.151801	1.149690
C	2.149134	-1.075248	-0.801208
O	2.528942	1.296167	-0.462250
H	1.846806	1.433065	-1.123686
H	4.377698	-0.231327	0.583171
H	3.310929	-1.064967	1.727356
H	3.535458	0.689121	1.835375
H	3.061903	-1.158861	-1.391788
H	1.971434	-2.023350	-0.292805
H	1.312334	-0.896764	-1.479780
H	1.361972	0.137705	0.784978
C	-1.527901	-1.446481	0.027060
C	-1.649467	-0.691554	-1.296884
C	-1.292331	0.784709	-1.121130
C	-2.386220	-0.796116	1.112830
C	-2.032487	0.681502	1.285300

C	-2.153326	1.436561	-0.038654
H	-0.480696	-1.438867	0.346722
H	-2.679441	-0.766702	-1.659026
H	-2.671274	1.138778	2.041934
H	-1.869608	2.481417	0.092991
H	-2.267642	-1.327194	2.058201
H	-1.810072	-2.491859	-0.105204
H	-1.015731	-1.151514	-2.057042
H	-1.396679	1.319110	-2.066454
H	-0.241742	0.862221	-0.820319
H	-3.440710	-0.880189	0.831920
H	-1.003162	0.763490	1.649154
H	-3.198670	1.428528	-0.362432

(8) IP-cyclopropane

Name: IP-cyclopropane
Charge: 0
Multiplicity: 1
E(UM062X) = -194.330652479 Ha

C	-1.496670	-0.030415	0.206527
C	-2.814504	-0.700180	0.543543
C	-1.678037	1.442779	-0.129472
O	-0.949548	-0.736392	-0.898543
H	-0.041189	-0.444394	-1.016495
H	-3.485622	-0.636847	-0.313507
H	-3.288292	-0.213162	1.395200
H	-2.656775	-1.750950	0.779371
H	-2.367563	1.544255	-0.967911
H	-2.079142	1.991712	0.723135
H	-0.727950	1.897304	-0.412809
H	-0.819822	-0.124236	1.065331
C	2.127896	-0.815465	-0.062809
C	1.991117	0.686642	-0.077829
C	3.255438	0.047161	0.416566
H	2.022219	1.192692	-1.031424
H	3.449325	0.077445	1.477378
H	2.249737	-1.322573	-1.008310
H	1.556877	-1.363741	0.671223
H	1.340961	1.139714	0.655676
H	4.132177	0.120785	-0.207666

(9) IP-1,4-dioxane

Name: IP-1,4-dioxane
Charge: 0
Multiplicity: 1
E(UM062X) = -194.330801202 Ha

O	-0.783516	0.192691	1.040834
C	-1.076000	-1.159438	0.714029
C	-1.912584	1.026909	0.832123
H	-1.848230	-1.538131	1.391956
H	-0.155364	-1.722311	0.864310
H	-1.602913	2.044807	1.062693
H	-2.715075	0.730247	1.515879
C	-1.557454	-1.255135	-0.719616
C	-2.390251	0.912347	-0.601954
H	-1.853422	-2.273971	-0.962028
H	-0.754171	-0.943167	-1.398502
H	-1.612363	1.285012	-1.279627
H	-3.300272	1.489878	-0.753474
O	-2.692920	-0.433146	-0.915578
C	2.212574	-0.083507	-0.200119
C	3.629904	-0.551656	-0.467404
C	1.942360	1.290331	-0.796225
O	2.040365	-0.071716	1.206983
H	1.145990	0.239620	1.389223
H	1.513697	-0.803851	-0.647366
H	2.061977	1.275989	-1.880266
H	2.638610	2.017073	-0.376169
H	0.925503	1.610828	-0.565397
H	3.820448	-0.617209	-1.538269
H	4.336154	0.152863	-0.027254
H	3.796707	-1.529421	-0.018633

(10) IP-DMAc

Name: IP-DMAc
Charge: 0
Multiplicity: 1
E(UM062X) = -194.330664341 Ha

C	-1.842865	-0.070517	0.230615
C	-3.008334	-0.836937	0.824719
C	-2.018123	1.435621	0.370629
O	-1.752889	-0.452419	-1.129586
H	-0.986140	0.001132	-1.511039
H	-3.926463	-0.563844	0.303808

H	-3.126299	-0.610651	1.883605
H	-2.854742	-1.909196	0.706617
H	-2.947848	1.738469	-0.111772
H	-2.057948	1.728042	1.421748
H	-1.196461	1.966371	-0.108237
H	-0.923473	-0.374668	0.752954
C	1.380044	0.643908	-0.401820
O	0.797210	0.842083	-1.461393
N	1.694302	-0.608564	0.022392
C	1.206226	-1.754544	-0.729421
C	2.166724	-0.900176	1.362322
C	1.786366	1.808053	0.476727
H	0.272410	-2.127803	-0.300642
H	1.958768	-2.541279	-0.700746
H	1.024982	-1.456933	-1.755710
H	2.820519	-1.770209	1.325602
H	1.334650	-1.121695	2.035876
H	2.733464	-0.068111	1.765715
H	2.862118	1.824061	0.645252
H	1.290110	1.757552	1.446200
H	1.487446	2.718947	-0.030769

(11) IP-DMSO

Name: IP-DMSO
Charge: 0
Multiplicity: 1
E(UM062X) = -553.172240795 Ha

C	-1.986590	0.025431	-0.190346
C	-3.185636	0.917296	-0.448598
C	-2.344359	-1.452042	-0.233442
O	-1.467496	0.386248	1.079146
H	-0.702615	-0.183978	1.244738
H	-3.945968	0.734555	0.311194
H	-3.614121	0.715196	-1.429787
H	-2.898718	1.966957	-0.398875
H	-3.104197	-1.666262	0.519013
H	-2.733347	-1.729369	-1.213855
H	-1.463012	-2.058198	-0.023970
H	-1.230054	0.221423	-0.966192
S	1.575360	-0.156037	-0.420410
C	3.359249	-0.268108	-0.171662
C	1.385599	1.558383	0.110725
H	3.567599	-0.048465	0.873006
H	3.861772	0.430318	-0.837434
H	3.646233	-1.288918	-0.407469
H	1.793880	1.649012	1.115307
H	0.317521	1.762717	0.126567
H	1.901229	2.208617	-0.592827
O	0.971054	-0.999095	0.671739

(12) IP-ethylene oxide

Name: IP-ethylene oxide
Charge: 0
Multiplicity: 1
E(UM062X) = -194.330779938 Ha

C	1.259726	-0.032381	-0.181592
C	2.636674	-0.519987	-0.588110
C	1.279388	1.416836	0.282172
O	0.805824	-0.891096	0.851447
H	-0.048945	-0.558829	1.148907
H	3.315209	-0.445328	0.261921
H	3.032590	0.078765	-1.407831
H	2.592308	-1.561684	-0.901214
H	1.970097	1.524581	1.118977
H	1.599023	2.079960	-0.522606
H	0.288278	1.728428	0.615631
H	0.589961	-0.119538	-1.049557
O	-1.919186	0.093548	0.683423
C	-2.205572	-0.812330	-0.377748
C	-2.205761	0.636053	-0.599235
H	-3.149276	-1.332782	-0.285415
H	-1.356697	-1.399738	-0.705152
H	-3.149264	1.161305	-0.665754
H	-1.363115	1.096103	-1.099778

(13) IP-H2O

Name: IP-H2O
Charge: 0
Multiplicity: 1
E(UM062X) = -194.330781233 Ha

O	2.615321	-0.061198	-0.381006
H	1.895379	-0.399813	0.169069
H	3.388515	-0.040694	0.183397
C	-0.598547	-0.005893	-0.249107

C	-0.423091	1.410111	0.258667
C	-2.057451	-0.418075	-0.331025
O	0.109224	-0.855322	0.667213
H	-0.053329	-1.770401	0.425163
H	-0.855089	1.501417	1.255588
H	-0.923480	2.112405	-0.406743
H	0.633855	1.666493	0.302626
H	-2.523332	-0.334461	0.651091
H	-2.595900	0.221824	-1.029468
H	-2.157056	-1.448490	-0.676598
H	-0.131389	-0.092980	-1.234991

(14) IP-HFB

Name: IP-HFB

Charge: 0

Multiplicity: 1

E(UM062X) = -194.330613026 Ha

C	2.194973	-0.079412	-0.956523
C	2.973299	-0.593647	-2.156551
C	2.708437	1.266838	-0.470419
O	2.197557	-1.035789	0.099036
H	3.102555	-1.165751	0.395251
H	4.029034	-0.706519	-1.899195
H	2.904259	0.102433	-2.992276
H	2.589383	-1.562004	-2.471782
H	3.768486	1.194470	-0.215739
H	2.600295	2.027818	-1.243187
H	2.168257	1.590881	0.418182
H	1.140862	0.012983	-1.230258
C	-0.623937	1.326772	0.685814
C	-0.134274	0.257911	1.414296
C	-0.482637	-1.036401	1.067896
C	-1.462446	1.105988	-0.394362
F	-0.279349	2.565358	1.013728
F	0.685699	0.474917	2.430403
F	-0.014799	-2.056286	1.762622
F	-1.931622	2.131857	-1.091697
C	-1.807055	-0.187026	-0.744369
C	-1.316152	-1.257458	-0.013578
F	-1.648124	-2.494025	-0.354396
F	-2.610120	-0.402864	-1.776939

(15) IP-HMB

Name: IP-HMB

Charge: 0

Multiplicity: 1

E(UM062X) = -194.330706918 Ha

C	-2.514365	-0.137528	-0.816641
C	-3.755476	-0.826096	-1.348303
C	-2.586914	1.372755	-0.978324
O	-1.413428	-0.671202	-1.533534
H	-0.597100	-0.342220	-1.135708
H	-3.863695	-0.615091	-2.413678
H	-4.647154	-0.473104	-0.829112
H	-3.675212	-1.905016	-1.218736
H	-2.715488	1.625422	-2.032452
H	-3.424209	1.787979	-0.413652
H	-1.665760	1.839151	-0.623227
H	-2.401936	-0.375527	0.250228
C	1.756132	0.797461	-0.414676
C	1.850145	-0.598855	-0.458236
C	1.098973	-1.375460	0.430382
C	0.862064	1.411064	0.470483
C	2.632356	1.642932	-1.307712
C	2.725764	-1.269898	-1.488958
C	1.231482	-2.877693	0.418665
C	0.695442	2.912146	0.441103
C	0.110109	0.633020	1.360374
C	0.205309	-0.762643	1.316587
C	-0.689165	-1.626340	2.173245
C	-0.794227	1.304342	2.366274
H	-0.175770	-1.984708	3.070418
H	-0.393092	2.267061	2.675727
H	3.585212	1.154249	-1.499798
H	2.851038	2.605020	-0.849548
H	2.160346	1.837900	-2.275475
H	2.292978	-2.214075	-1.813470
H	3.725742	-1.481409	-1.098187
H	2.841713	-0.650173	-2.374948
H	2.231815	-3.186384	0.122843
H	0.521678	-3.330102	-0.281246
H	1.039179	-3.300457	1.402483
H	0.805108	3.297718	-0.571264
H	1.438347	3.416049	1.066888
H	-0.288423	3.214057	0.793007
H	-1.022522	-2.501203	1.615644
H	-1.580843	-1.093305	2.493168
H	-0.904634	0.702367	3.264976

H	-1.795677	1.480174	1.957803

(16) IP-acetic acid

Name: IP-acetic acid
Charge: 0
Multiplicity: 1
E(UM062X) = -194.330847866 Ha

C	-1.871465	-0.152823	-0.177465
C	-3.354447	-0.131690	-0.494770
C	-1.595162	-0.712313	1.210903
O	-1.410432	1.182822	-0.286180
H	-0.478789	1.205558	-0.037760
H	-3.877309	0.488080	0.234453
H	-3.771551	-1.138188	-0.463787
H	-3.522500	0.289017	-1.484862
H	-2.109272	-0.109341	1.960382
H	-1.940973	-1.743700	1.291828
H	-0.526196	-0.689176	1.425450
H	-1.357875	-0.777860	-0.917223
O	1.350056	-0.858466	-0.694761
C	2.052093	0.088224	-0.038847
C	3.540166	-0.125057	0.035454
O	1.475589	1.021848	0.438252
H	3.757241	-1.070291	0.533066
H	3.990331	0.691368	0.588165
H	3.960054	-0.163342	-0.969788
H	1.928026	-1.549804	-1.030058

(17) IP-naphthalene

Name: IP-naphthalene
Charge: 0
Multiplicity: 1
E(UM062X) = -194.330849780 Ha

C	0.432283	2.493646	0.838079
C	0.882415	2.617425	-0.496986
C	1.399803	1.535301	-1.154361
C	1.492560	0.273978	-0.512011
C	1.035314	0.149385	0.826074
C	0.507427	1.290420	1.481404
C	2.018766	-0.867584	-1.167849
C	2.083324	-2.072486	-0.526203
C	1.621121	-2.198298	0.804331
C	1.109816	-1.115221	1.462189
H	0.015966	3.354037	1.342038
H	0.808779	3.572028	-0.998299
H	1.742269	1.625222	-2.176999
H	0.155610	1.188718	2.499953
H	2.366065	-0.767591	-2.187957
H	2.485105	-2.937159	-1.035415
H	1.672884	-3.158406	1.298252
H	0.753998	-1.206956	2.480388
C	-2.353823	-0.365386	0.014353
C	-1.796175	-1.592324	-0.690469
C	-3.858352	-0.442560	0.186896
O	-2.093645	0.814022	-0.734558
H	-1.141695	0.934763	-0.803277
H	-2.274830	-1.704970	-1.664092
H	-1.977317	-2.493026	-0.102530
H	-0.719963	-1.499045	-0.841591
H	-4.336339	-0.511871	-0.790773
H	-4.133727	-1.318500	0.773532
H	-4.229520	0.449564	0.688349
H	-1.884991	-0.276752	1.002201

(18) IP-Et2O

Name: IP-Et2O
Charge: 0
Multiplicity: 1
E(UM062X) = -194.330783802 Ha

O	1.465726	0.057620	0.105040
C	2.298493	-1.039285	-0.225998
C	2.104464	1.313538	-0.034737
H	3.181355	-1.031685	0.422439
C	1.499571	-2.310939	-0.045039
H	2.641467	-0.936216	-1.261390
H	2.490899	1.416108	-1.054740
H	2.954807	1.365192	0.653818
C	1.089119	2.393238	0.268363
H	2.101435	-3.177172	-0.315698
H	0.610324	-2.291134	-0.674663
H	1.184350	-2.415599	0.992092
H	0.261058	2.341668	-0.438286
H	1.548388	3.377922	0.196502
H	0.694976	2.265454	1.276019

C -2.036547 0.247518 0.076190
C -1.748896 -0.703899 1.228983
C -3.453008 0.101866 -0.444753
O -1.165609 -0.002367 -1.015482
H -0.258696 0.004019 -0.680370
H -1.894251 1.278228 0.427180
H -1.886080 -1.734541 0.897358
H -0.720665 -0.585524 1.573160
H -2.419516 -0.513896 2.067704
H -4.178007 0.324012 0.337896
H -3.612470 -0.919499 -0.792114
H -3.619482 0.778416 -1.281426

(19) IP-Me2O

Name: IP-Me2O
Charge: 0
Multiplicity: 1
E(UM062X) = -194.330766279 Ha

O -1.749748 -0.115973 0.339173
C -1.858215 -1.245004 -0.499568
C -2.660681 0.899814 -0.009128
H -2.843407 -1.709410 -0.399305
H -1.088438 -1.949445 -0.192749
H -1.700636 -0.967073 -1.546435
H -3.691439 0.541242 0.066370
H -2.480799 1.250030 -1.030399
H -2.515845 1.723580 0.684967
C 1.420467 -0.039333 -0.170901
C 2.856918 -0.495495 -0.337573
C 1.296658 1.477406 -0.194016
O 0.961003 -0.566708 1.062879
H 0.039342 -0.301032 1.161475
H 0.818726 -0.453495 -0.990874
H 3.265708 -0.148065 -1.286000
H 3.464457 -0.094656 0.474349
H 2.915467 -1.582117 -0.304907
H 1.645719 1.883549 -1.144087
H 1.892897 1.907336 0.611549
H 0.257320 1.776680 -0.053253

(20) IP-PhH

Name: IP-PhH
Charge: 0
Multiplicity: 1
E(UM062X) = -194.330817418 Ha

C -2.032065 -0.101402 0.129123
C -3.348498 -0.851990 0.075548
C -2.221174 1.392155 -0.091923
O -1.204317 -0.663618 -0.878096
H -0.327039 -0.273848 -0.811721
H -3.809671 -0.712946 -0.902767
H -4.032859 -0.485640 0.840151
H -3.183897 -1.916920 0.229468
H -2.690364 1.563214 -1.061343
H -2.852746 1.824200 0.685144
H -1.260721 1.908671 -0.079216
H -1.567902 -0.262584 1.109759
C 1.290197 0.900412 1.110184
C 1.669177 1.379313 -0.138777
C 2.165308 0.503177 -1.097790
C 1.407799 -0.453798 1.400005
C 1.902099 -1.329393 0.441265
C 2.281261 -0.851388 -0.807234
H 0.898488 1.580931 1.853885
H 1.574554 2.432210 -0.366170
H 2.455431 0.874405 -2.070673
H 1.981533 -2.384258 0.662448
H 2.657750 -1.534058 -1.555826
H 1.107359 -0.826952 2.369217

(21) IP- anisole

Name: IP- anisole
Charge: 0
Multiplicity: 1
E(UM062X) = -194.330675760 Ha

C 2.144860 -0.194047 0.072412
C 2.059804 0.911339 -0.969632
C 3.577790 -0.479799 0.477853
O 1.616186 -1.410315 -0.437421
H 0.668223 -1.306696 -0.567787
H 2.670045 0.652147 -1.835553
H 2.408781 1.860729 -0.561255
H 1.029681 1.036054 -1.305017
H 4.146322 -0.809268 -0.392429

H	4.048492	0.414509	0.885259
H	3.609516	-1.268385	1.227484
H	1.575055	0.108656	0.958127
C	-1.121164	-0.467172	1.345767
C	-1.472222	-1.753068	0.980458
C	-1.907869	-2.027000	-0.314957
C	-1.990543	-0.992954	-1.232908
C	-1.640029	0.308998	-0.879812
C	-1.197957	0.569878	0.414078
H	-0.772619	-0.237671	2.342685
H	-1.396412	-2.550630	1.705871
H	-2.174483	-3.033892	-0.600145
H	-2.325570	-1.190390	-2.241620
H	-1.705230	1.096628	-1.614690
O	-0.798306	1.790480	0.854131
C	-0.990222	2.893654	-0.008147
H	-2.041160	2.992772	-0.286995
H	-0.380316	2.805043	-0.908966
H	-0.678049	3.770091	0.550673

(22) IP-sulfolane

Name: IP-sulfolane
Charge: 0
Multiplicity: 1
E(UM062X) = -705.807766753 Ha

C	-2.829079	-0.036214	0.077636
C	-4.272684	-0.027993	0.542190
C	-2.308570	1.375283	-0.162205
O	-2.781548	-0.796291	-1.121001
H	-1.855337	-0.993956	-1.293355
H	-4.903544	0.418908	-0.227289
H	-4.379834	0.548447	1.460894
H	-4.618105	-1.044627	0.721048
H	-2.960102	1.896959	-0.864639
H	-2.261939	1.940000	0.769660
H	-1.308769	1.340201	-0.596885
H	-2.206323	-0.515639	0.838764
S	0.899178	-0.319453	0.248798
C	1.524630	1.259693	-0.382237
C	2.510563	-1.119219	0.422282
H	1.013151	2.048402	0.160872
H	1.259249	1.286513	-1.436605
H	2.397852	-2.144814	0.084826
H	2.752216	-1.086685	1.482285
O	0.158485	-0.991300	-0.804618
O	0.293174	-0.130838	1.547016
C	3.452587	-0.259150	-0.414283
C	3.034893	1.197557	-0.163233
H	3.334923	-0.502494	-1.471087
H	4.490708	-0.436732	-0.142626
H	3.552997	1.889328	-0.823537
H	3.271082	1.475131	0.864821

(23) IP-THF

Name: IP-THF
Charge: 0
Multiplicity: 1
E(UM062X) = -194.330796062 Ha

O	-1.279499	0.474742	-0.971812
C	-1.969877	-0.780052	-0.956865
C	-1.585320	1.206620	0.225607
H	-1.324469	-1.521510	-1.424131
H	-2.893916	-0.691976	-1.534739
H	-1.984270	2.181136	-0.052060
H	-0.655141	1.346906	0.779964
C	-2.256237	-1.055828	0.510715
C	-2.578703	0.352643	1.013889
H	-1.355084	-1.436212	0.992145
H	-3.068055	-1.764474	0.656937
H	-3.603193	0.619627	0.753397
H	-2.456292	0.467471	2.088041
C	2.359179	-0.120511	-0.331069
C	3.370259	-0.830558	0.547233
C	2.326168	1.378160	-0.068134
O	1.093410	-0.704279	-0.063857
H	0.415914	-0.214263	-0.551804
H	2.622477	-0.289714	-1.382069
H	2.086010	1.563360	0.980102
H	3.290289	1.835976	-0.290801
H	1.568621	1.860958	-0.688121
H	3.121127	-0.671100	1.596948
H	3.356590	-1.901065	0.349575
H	4.375291	-0.451669	0.363716

(24) IP-trimethylamine

Name: IP-trimethylamine
Charge: 0
Multiplicity: 1
E(UM062X) = -194.330577660 Ha

N	1.633862	-0.025953	-0.042991
C	2.348510	-1.240417	-0.412487
C	1.454220	0.040463	1.400349
C	2.328391	1.153958	-0.533444
H	1.779103	-2.109499	-0.085353
H	3.348848	-1.279347	0.042305
H	2.455689	-1.285845	-1.495121
H	0.888415	0.935846	1.658396
H	2.416156	0.068399	1.932281
H	0.892882	-0.831509	1.735163
H	3.337290	1.241420	-0.104313
H	1.764180	2.048403	-0.271647
H	2.415133	1.104599	-1.617674
C	-1.869488	-0.040033	0.219028
C	-3.293190	-0.544019	0.079783
C	-1.736610	1.411700	-0.222216
O	-1.046511	-0.883761	-0.564159
H	-0.142388	-0.521476	-0.541426
H	-3.605268	-0.473545	-0.962706
H	-3.975853	0.044883	0.692157
H	-3.355354	-1.586934	0.386051
H	-2.043812	1.508646	-1.264234
H	-2.357103	2.066973	0.390081
H	-0.700173	1.744468	-0.140705
H	-1.573696	-0.113636	1.274870

Cartesian coordinates of optimized geometries of $CHCl_3$/H-bond acceptors

(1) CHCl3-acetone

Name: CHCl3-acetone
Charge: 0
Multiplicity: 1
E(UM062X) = -1419.28307753 Ha

C	2.578688	1.224957	-0.427328
C	2.343628	0.041717	0.476169
C	3.038606	-1.243313	0.103657
O	1.624459	0.115973	1.445197
H	2.041085	1.052327	-1.362882
H	3.636709	1.322240	-0.670491
H	2.203135	2.131672	0.036749
H	4.111145	-1.129772	0.272447
H	2.897594	-1.453333	-0.956851
H	2.658068	-2.062468	0.705962
C	-1.113090	-0.053280	0.220323
Cl	-2.787523	-0.509896	0.544555
Cl	-0.387539	-1.142779	-0.980083
Cl	-1.006846	1.625199	-0.326460
H	-0.537961	-0.141852	1.130355

(2) CHCl3-acetonitrile

Name: CHCl3-acetonitrile
Charge: 0
Multiplicity: 1
E(UM062X) = -132.737915411 Ha

C	-4.923985	-0.005473	-0.077184
C	-3.471120	-0.013168	-0.205536
N	-2.330953	-0.019261	-0.306522
H	-5.202165	0.344075	0.914132
H	-5.309918	-1.011240	-0.224824
H	-5.354217	0.655497	-0.825660
C	0.974773	-0.005364	-0.062354
Cl	1.096047	-0.698357	1.562128
Cl	1.518144	1.679669	-0.057127
Cl	1.901738	-0.962826	-1.228359
H	-0.065827	-0.023741	-0.350458

(3) CHCl3-ethane

Name: CHCl3-ethane
Charge: 0
Multiplicity: 1
E(UM062X) = -1419.28313727 Ha

C	0.396368	-0.013265	0.163898
Cl	0.274690	1.538337	-0.679137
Cl	0.209954	-1.358345	-0.969946
Cl	1.921211	-0.137392	1.047358
H	-0.418276	-0.066419	0.871093
C	-2.937597	0.071420	0.024986

C	-3.183714	-0.154018	1.171872
H	-2.716169	0.272096	-0.996211
H	-3.415426	-0.354702	2.189906

(4) CHCl3-ethylene

Name: CHCl3-ethylene
Charge: 0
Multiplicity: 1
E(UM062X) = -1419.28313733 Ha

C	0.484403	-0.021282	-0.178138
Cl	0.434123	1.644299	0.414534
Cl	2.052384	-0.388668	-0.904550
Cl	0.106809	-1.162387	1.122077
H	-0.280388	-0.123563	-0.934043
C	-2.897689	0.477570	-0.195400
C	-2.940489	-0.643987	-0.898560
H	-2.532423	0.491170	0.823504
H	-3.227495	1.420579	-0.610705
H	-3.309910	-0.661475	-1.915310
H	-2.613512	-1.585671	-0.475904

(5) CHCl3-acetylene

Name: CHCl3-acetylene
Charge: 0
Multiplicity: 1
E(UM062X) = -1419.28314897 Ha

C	-0.549945	-0.019238	-0.141806
Cl	-0.854327	1.682074	0.230131
Cl	-0.115118	-0.917661	1.318158
Cl	-1.943919	-0.752637	-0.943681
H	0.289221	-0.063731	-0.820522
C	2.770394	0.737572	-0.357449
C	3.064926	-0.740121	-0.599956
H	3.692451	1.301454	-0.223602
H	2.166785	0.882083	0.538997
H	3.599391	-1.174538	0.243656
H	3.676793	-0.878804	-1.490281
H	2.237920	1.186901	-1.197368
H	2.152369	-1.322841	-0.733947

(6) CHCl3-cubane

Name: CHCl3-cubane
Charge: 0
Multiplicity: 1
E(UM062X) = -1419.28314078 Ha

C	-1.529028	-0.680853	-0.948097
C	-3.087938	-0.673623	-0.871515
C	-1.514488	0.872158	-0.748193
C	-3.073514	0.874454	-0.672367
C	-3.015193	-0.873080	0.674551
C	-3.000691	0.674959	0.873705
C	-1.441307	0.671488	0.802000
C	-1.455937	-0.880859	0.602256
H	-0.941356	-1.226740	-1.679632
H	-3.746296	-1.213313	-1.542797
H	-0.915200	1.574896	-1.318882
H	-3.720288	1.578146	-1.184040
H	-3.614677	-1.572881	1.245650
H	-3.588362	1.218919	1.604452
H	-0.781831	1.210487	1.475464
H	-0.809093	-1.584640	1.117010
C	1.735360	-0.001069	-0.027096
Cl	2.052151	-0.306974	1.684945
Cl	2.332804	1.591581	-0.508631
Cl	2.423650	-1.277475	-1.038306
H	0.667230	-0.007578	-0.174812

(7) CHCl3-cyclohexane

Name: CHCl3-cyclohexane
Charge: 0
Multiplicity: 1
E(UM062X) = -1419.28313555 Ha

C	2.184510	1.172798	1.012061
C	2.583553	1.323152	-0.452581
C	2.059575	0.161012	-1.294955
C	2.638555	-0.173515	1.576832
C	2.114379	-1.340557	0.739473
C	2.508654	-1.188798	-0.730856
H	1.096263	1.258183	1.101100
H	3.676179	1.349375	-0.517684

H	2.483133	-2.286745	1.136229
H	2.082418	-2.002724	-1.318937
H	2.318339	-0.279747	2.614560
H	2.603819	1.988407	1.602621
H	2.219947	2.271949	-0.848817
H	2.391856	0.264774	-2.328625
H	0.966640	0.199322	-1.317925
H	3.732673	-0.200384	1.579450
H	1.021052	-1.386215	0.814488
H	3.597356	-1.266630	-0.814721
C	-1.561668	0.005107	0.139505
Cl	-2.946330	-0.144684	1.227017
Cl	-1.480877	-1.369950	-0.971968
Cl	-1.613660	1.533810	-0.747641
H	-0.660265	0.009249	0.735450

(8) CHCl3-cyclopropane

Name: CHCl3-cyclopropane
Charge: 0
Multiplicity: 1
E(UM062X) = -1419.28315087 Ha

C	-2.702732	0.755172	0.796788
C	-2.891913	-0.007083	-0.481192
C	-2.701234	-0.746564	0.810866
H	-3.873918	-0.012292	-0.928113
H	-1.767945	-1.269329	0.959387
H	-3.560252	1.255231	1.219815
H	-1.770787	1.281971	0.934564
H	-2.073194	-0.012214	-1.185078
H	-3.557717	-1.240614	1.242769
C	0.823018	-0.000215	0.174036
Cl	2.404545	0.000619	0.962600
Cl	0.603296	1.456182	-0.804958
Cl	0.602507	-1.457359	-0.804189
H	0.065067	-0.001128	0.944960

(9) CHCl3-1,4-dioxane

Name: CHCl3-1,4-dioxane
Charge: 0
Multiplicity: 1
E(UM062X) = -1419.28307258 Ha

O	-1.236008	-0.089543	-0.738888
C	-1.898095	-1.264741	-0.293748
C	-1.653067	1.034185	0.025262
H	-1.616954	-1.470513	0.744770
H	-1.556419	-2.082470	-0.926332
H	-1.138423	1.903130	-0.380507
H	-1.352684	0.894692	1.070160
C	-3.399980	-1.088574	-0.392903
C	-3.156800	1.200855	-0.066738
H	-3.918378	-1.959210	0.004510
H	-3.686091	-0.947939	-1.442018
H	-3.438350	1.418445	-1.103889
H	-3.494552	2.014616	0.572244
O	-3.815765	0.027503	0.370913
C	1.753561	-0.053259	-0.161361
Cl	1.275266	-0.793933	1.375626
Cl	1.946069	1.697553	0.027593
Cl	3.237070	-0.787322	-0.779461
H	0.949418	-0.222286	-0.862085

(10) CHCl3-DMAc

Name: CHCl3-DMAc
Charge: 0
Multiplicity: 1
E(UM062X) = -1419.28302973 Ha

C	-1.927590	-0.663657	-0.695792
O	-1.091097	-0.749784	-1.585398
N	-2.331981	0.538149	-0.203843
C	-1.659688	1.746061	-0.648475
C	-3.104834	0.683893	1.013228
C	-2.555292	-1.910193	-0.112859
H	-0.913764	2.064081	0.085431
H	-2.392996	2.542782	-0.769777
H	-1.169142	1.555613	-1.596620
H	-3.755048	1.553148	0.919910
H	-2.448915	0.836563	1.875844
H	-3.726760	-0.185599	1.192388
H	-3.641369	-1.887739	-0.188293
H	-2.286477	-2.017572	0.938129
H	-2.169857	-2.757358	-0.669057
C	1.488954	-0.061759	-0.104499
Cl	0.577513	-0.232119	1.414417
Cl	2.108173	1.588798	-0.270298

Cl	2.803124	-1.237910	-0.171923
H	0.797898	-0.257848	-0.914811

(11) CHCl3-DMSO

Name: CHCl3-DMSO
Charge: 0
Multiplicity: 1
E(UM062X) = -1419.28297416 Ha

S	2.109501	-0.171834	-0.094304
C	3.675363	-1.008712	0.234711
C	2.756905	1.512765	-0.069398
H	4.064977	-0.638215	1.180249
H	4.365929	-0.823763	-0.585333
H	3.452351	-2.069323	0.308371
H	3.227239	1.680198	0.897055
H	1.901490	2.170938	-0.193360
H	3.460503	1.645667	-0.888443
O	1.310230	-0.314727	1.170450
C	-1.490530	-0.054502	0.200175
Cl	-3.130688	0.097428	0.839252
Cl	-1.348263	-1.469619	-0.851663
Cl	-1.025211	1.418135	-0.680332
H	-0.786024	-0.176690	1.010425

(12) CHCl3-ethylene oxide

Name: CHCl3-ethylene oxide
Charge: 0
Multiplicity: 1
E(UM062X) = -1419.28305913 Ha

O	2.065146	0.189842	-0.977031
C	2.751896	0.563439	0.209786
C	3.017531	-0.720450	-0.444340
H	2.140011	0.582569	1.102653
H	3.456184	1.375572	0.087902
H	2.595815	-1.622282	-0.019890
H	3.914651	-0.837593	-1.037664
C	-0.822962	-0.022729	-0.225446
Cl	-0.702737	1.612876	0.437911
Cl	-0.233916	-1.216312	0.948108
Cl	-2.482660	-0.388564	-0.707670
H	-0.178294	-0.074565	-1.088679

(13) CHCl3-H2Obsse

Name: CHCl3-H2Obsse
Charge: 0
Multiplicity: 1
E(UM062X) = -76.4241122226 Ha

C	-0.222012	-0.000007	0.228355
Cl	0.193073	-1.456783	-0.692975
Cl	0.193008	1.456860	-0.692902
Cl	-1.931089	-0.000061	0.666731
H	0.381192	-0.000040	1.123452
O	2.681834	-0.000032	1.082979
H	2.880769	-0.761770	0.533966
H	2.880579	0.761842	0.534110

(14) CHCl3-HFB

Name: CHCl3-HFB
Charge: 0
Multiplicity: 1
E(UM062X) = -1419.28314937 Ha

C	1.892152	-0.000137	-0.398562
Cl	2.052939	1.456834	0.592410
Cl	3.058908	0.000076	-1.722678
Cl	2.052312	-1.457893	0.591387
H	0.899304	0.000226	-0.827889
C	-1.266378	1.200012	0.793968
C	-1.038514	0.000159	1.447708
C	-1.266879	-1.199678	0.794074
C	-1.714975	1.200316	-0.516616
F	-1.048084	2.344856	1.417765
F	-0.594358	0.000134	2.692703
F	-1.048931	-2.344535	1.417967
F	-1.931810	2.346182	-1.142618
C	-1.940589	0.000229	-1.171606
C	-1.715521	-1.199910	-0.516476
F	-1.932802	-2.345708	-1.142436
F	-2.375767	0.000242	-2.421833

(15) CHCl3-HMB

Name: CHCl3-HMB
Charge: 0
Multiplicity: 1
E(UM062X) = -1419.28313708 Ha

C	1.312098	0.033433	-1.462151
C	1.347469	-1.196567	-0.791790
C	1.480903	-1.229780	0.602395
C	1.514745	-0.033452	1.331672
C	1.485712	1.196409	0.660247
C	1.352687	1.229821	-0.734258
C	-1.712616	0.000501	0.112255
Cl	-2.298771	-0.001654	1.783270
Cl	-2.229345	-1.458681	-0.746297
Cl	-2.232043	1.459824	-0.744090
H	-0.636301	0.001913	0.143150
C	1.588389	-2.555696	1.320152
H	0.604518	-2.949196	1.590626
H	2.080598	-3.299544	0.699121
H	2.169221	-2.463273	2.233907
C	1.531345	-0.070389	2.843026
H	1.007423	-0.945353	3.218996
H	2.549984	-0.097532	3.238708
H	1.036672	0.801570	3.262531
C	1.598582	2.486592	1.439605
H	0.617542	2.860582	1.745729
H	2.194970	2.352872	2.338201
H	2.076149	3.262942	0.847837
C	1.200483	2.554293	-1.446363
H	0.707242	3.285691	-0.811306
H	2.166988	2.969076	-1.744085
H	0.593462	2.450439	-2.341805
C	1.228152	0.069288	-2.970627
H	0.189830	0.088408	-3.314018
H	1.724120	0.948875	-3.372283
H	1.705749	-0.800735	-3.413107
C	1.189518	-2.483768	-1.568274
H	0.556236	-2.338349	-2.439818
H	2.151439	-2.869506	-1.915632
H	0.722086	-3.254311	-0.961084

(16) CHCl3-acetic acid

Name: CHCl3-acetic acid
Charge: 0
Multiplicity: 1
E(UM062X) = -1419.28298263 Ha

O	-2.345949	0.738271	0.772801
C	-2.540227	-0.003696	-0.325309
C	-3.949665	-0.502813	-0.425415
O	-1.666130	-0.227684	-1.122434
H	-4.633671	0.344007	-0.434091
H	-4.181413	-1.104146	0.452049
H	-4.062527	-1.091308	-1.328455
H	-1.421204	1.029070	0.778731
C	1.205885	-0.096582	-0.240688
Cl	0.740356	-1.219209	1.042004
Cl	0.933576	1.584027	0.284584
Cl	2.887271	-0.327768	-0.716958
H	0.559035	-0.273619	-1.086399

(17) CHCl3-naphthalene

Name: CHCl3-naphthalene
Charge: 0
Multiplicity: 1
E(UM062X) = -1419.28314751 Ha

C	-0.739112	2.726261	-0.673530
C	-0.534506	2.642048	0.723897
C	-0.936580	1.531195	1.413244
C	-1.559943	0.448153	0.742996
C	-1.781431	0.540085	-0.656332
C	-1.350631	1.702540	-1.344246
C	-1.946270	-0.733453	1.423799
C	-2.540089	-1.762562	0.749394
C	-2.779782	-1.662151	-0.640524
C	-2.407267	-0.540840	-1.325898
H	-0.409286	3.605209	-1.209250
H	-0.052556	3.458347	1.243231
H	-0.769444	1.455541	2.479714
H	-1.510759	1.764188	-2.412813
H	-1.757697	-0.806330	2.486963
H	-2.828349	-2.661069	1.276579
H	-3.253628	-2.482982	-1.160365
H	-2.576668	-0.464668	-2.392061
C	1.631267	-0.231893	-0.093147
Cl	3.119907	0.275474	-0.905252

Cl	0.955831	-1.678551	-0.848194
Cl	1.919171	-0.502365	1.633419
H	0.910991	0.567980	-0.189465

(18) CHCl3-Et2O

Name: CHCl3-Et2O
Charge: 0
Multiplicity: 1
E(UM062X) = -1419.28303764 Ha

O	1.920452	0.000364	-0.468458
C	2.474447	1.176125	0.088376
C	2.475036	-1.174919	0.088790
H	3.546621	1.214879	-0.135850
C	1.756118	2.372102	-0.494300
H	2.359397	1.150802	1.178341
H	2.359647	-1.149432	1.178717
H	3.547304	-1.213032	-0.135104
C	1.757691	-2.371501	-0.493846
H	2.196850	3.296199	-0.123103
H	0.703280	2.360693	-0.213390
H	1.827749	2.364872	-1.581023
H	0.704728	-2.360664	-0.213405
H	2.198858	-3.295209	-0.122187
H	1.829792	-2.364557	-1.580540
C	-1.130771	-0.000066	-0.098988
Cl	-0.576830	-0.000526	1.584909
Cl	-2.075474	-1.455155	-0.449643
Cl	-2.076481	1.454601	-0.448558
H	-0.243624	0.000450	-0.719047

(19) CHCl3-Me2O

Name: CHCl3-Me2O
Charge: 0
Multiplicity: 1
E(UM062X) = -155.001705353 Ha

O	-2.160748	0.011653	-0.798690
C	-2.554260	1.107112	-0.002217
C	-2.831929	-1.170034	-0.425593
H	-3.633758	1.271284	-0.074610
H	-2.030696	1.985620	-0.369576
H	-2.287254	0.940224	1.046416
H	-3.909995	-1.069666	-0.584022
H	-2.646556	-1.408529	0.626051
H	-2.451433	-1.976038	-1.048175
C	0.814918	-0.041922	-0.161526
Cl	1.058178	1.690060	0.117089
Cl	2.288052	-0.793072	-0.784637
Cl	0.280370	-0.841428	1.327261
H	0.021115	-0.151587	-0.885672

(20) CHCl3-PhH

Name: CHCl3-PhH
Charge: 0
Multiplicity: 1
E(UM062X) = -1419.28314940 Ha

C	2.133196	0.250741	-1.344620
C	2.139862	-1.060803	-0.881769
C	2.171277	-1.315954	0.484531
C	2.197170	-0.259224	1.388335
C	2.191853	1.052349	0.925558
C	2.160165	1.307051	-0.441020
H	2.100514	0.448813	-2.406830
H	2.109926	-1.881772	-1.584351
H	2.167943	-2.335264	0.844527
H	2.212965	-0.457439	2.450919
H	2.205814	1.873722	1.628204
H	2.146640	2.326370	-0.800649
C	-1.108232	0.000080	0.015842
Cl	-1.767443	-0.368150	1.616205
Cl	-1.580513	-1.231852	-1.162347
Cl	-1.606335	1.610990	-0.518911
H	-0.032597	-0.006660	0.092924

(21) CHCl3- anisole

Name: CHCl3- anisole
Charge: 0
Multiplicity: 1
E(UM062X) = -1419.28314736 Ha

C	-1.773551	0.895088	-1.297796
C	-1.324951	2.171219	-1.013449

C	-1.082075	2.560667	0.304398
C	-1.297900	1.651773	1.326668
C	-1.747583	0.360355	1.056067
C	-1.988783	-0.018248	-0.261791
H	-1.956967	0.571866	-2.312506
H	-1.156246	2.867469	-1.823138
H	-0.727565	3.557124	0.523366
H	-1.106146	1.934756	2.352397
H	-1.893160	-0.332549	1.869958
O	-2.418467	-1.246473	-0.637176
C	-2.508532	-2.237719	0.366219
H	-3.261170	-1.976259	1.112608
H	-1.542142	-2.384695	0.853770
H	-2.804264	-3.151556	-0.138906
C	1.449229	-0.096337	-0.037550
Cl	0.952877	-1.578001	-0.867894
Cl	1.746052	-0.411051	1.679762
Cl	2.877484	0.609509	-0.805110
H	0.641264	0.617083	-0.111610

(22) CHCl3-sulfolane

Name: CHCl3-sulfolane
Charge: 0
Multiplicity: 1
E(UM062X) = -1419.28308224 Ha

S	1.487037	0.307367	0.353788
C	2.098156	-0.758352	-0.978505
C	3.108577	0.836143	0.945377
H	1.554468	-0.489169	-1.878322
H	1.851218	-1.772939	-0.675199
H	3.049339	0.898898	2.027600
H	3.289839	1.817242	0.511488
O	0.856544	-0.510280	1.372235
O	0.752767	1.432050	-0.187550
C	4.062825	-0.222733	0.404603
C	3.604699	-0.507127	-1.034041
H	3.987460	-1.128990	1.007079
H	5.094153	0.120866	0.441389
H	4.125297	-1.359308	-1.465057
H	3.811351	0.361586	-1.660233
C	-2.078993	0.028663	0.116097
Cl	-3.261736	-0.830429	1.111671
Cl	-1.407985	-1.056820	-1.120263
Cl	-2.797224	1.455306	-0.633614
H	-1.263723	0.343258	0.749481

(23) CHCl3-THF

Name: CHCl3-THF
Charge: 0
Multiplicity: 1
E(UM062X) = -232.416560039 Ha

O	-1.485213	0.196905	-1.080748
C	-2.129042	-1.065911	-0.881710
C	-2.044225	1.175429	-0.199907
H	-1.364940	-1.833512	-0.754421
H	-2.717753	-1.299634	-1.771468
H	-2.152160	2.106696	-0.752514
H	-1.361213	1.338242	0.638855
C	-3.017276	-0.901278	0.347597
C	-3.363750	0.587145	0.280721
H	-2.448686	-1.112383	1.253433
H	-3.887119	-1.553269	0.322489
H	-4.147433	0.762222	-0.457029
H	-3.683443	1.001250	1.234006
C	1.364259	-0.029227	-0.148462
Cl	0.678941	-0.811093	1.290496
Cl	1.661852	1.687685	0.160920
Cl	2.845214	-0.846340	-0.658801
H	0.622538	-0.096081	-0.931244

(24) CHCl3-trimethylamine

Name: CHCl3-trimethylamine
Charge: 0
Multiplicity: 1
E(UM062X) = -1419.28299224 Ha

N	2.102651	0.000604	0.242675
C	2.529065	-1.390722	0.259085
C	2.871990	0.784064	1.196403
C	2.231963	0.556301	-1.097107
H	2.392163	-1.806182	1.256829
H	3.588254	-1.497161	-0.020145
H	1.926008	-1.966826	-0.441776
H	2.516621	1.813743	1.196995
H	3.945450	0.785668	0.953703

H	2.745993	0.373394	2.197354
H	3.272943	0.525169	-1.452675
H	1.894151	1.591634	-1.099567
H	1.610324	-0.009418	-1.790781
C	-1.030991	0.002615	0.113798
Cl	-1.292475	1.603805	-0.599047
Cl	-2.209582	-0.320769	1.391062
Cl	-1.097667	-1.254656	-1.136604
H	-0.027316	-0.010248	0.526285

Cartesian coordinates of optimized geometries of HFIP/PhMen complexes (n = 1-6)

(1) HFIP-Dur

Name: HFIP-Dur
Charge: 0
Multiplicity: 1
E(UM062X) = -789.824192527 Ha

C	-1.139797	-0.000483	-0.381326
C	-1.845053	-1.273478	0.067805
C	-1.847620	1.271734	0.066631
O	-1.050145	-0.002518	-1.770711
H	-0.116943	0.015011	-2.004558
F	-3.067793	-1.389135	-0.437909
F	-1.935293	-1.338871	1.397944
F	-1.133266	-2.331887	-0.337907
F	-3.073242	1.381746	-0.433163
F	-1.931055	1.342334	1.396980
F	-1.141041	2.330818	-0.346980
H	-0.167477	0.001299	0.108365
C	1.952388	-1.221769	0.784846
C	1.907443	0.000157	1.451699
C	1.950932	1.222726	0.785996
C	2.070308	-1.222129	-0.610973
C	2.123771	-2.516442	-1.374930
C	2.139393	0.001498	-1.275110
C	2.069269	1.224482	-0.609900
H	1.219646	-3.104558	-1.211165
H	2.968406	-3.127293	-1.052609
H	2.223984	-2.336470	-2.443269
C	1.860322	-2.517292	1.542935
H	1.812922	-0.000395	2.531394
C	1.857179	2.517495	1.545198
H	2.262166	0.001724	-2.354078
C	2.122387	2.519642	-1.372564
H	1.772187	-2.337922	2.611986
H	2.741240	-3.138062	1.372016
H	0.991961	-3.094955	1.221183
H	0.988207	3.094479	1.223825
H	2.737382	3.139491	1.375035
H	1.769004	2.337073	2.614063
H	2.967195	3.130093	-1.049918
H	1.218496	3.107917	-1.207971
H	2.222460	2.340808	-2.441137

(2) HFIP-Mes

Name: HFIP-Mes
Charge: 0
Multiplicity: 1
E(UM062X) = -789.824504724 Ha

C	-1.041806	-0.038113	-0.368122
C	-1.968639	-1.159194	0.079583
C	-1.537270	1.344919	0.045655
O	-0.926250	-0.112340	-1.753307
H	0.002037	0.011112	-1.975858
F	-3.130413	-1.147017	-0.562542
F	-2.215821	-1.087509	1.390109
F	-1.381174	-2.335507	-0.158679
F	-2.787523	1.581757	-0.339839
F	-1.473376	1.505316	1.370095
F	-0.755447	2.275928	-0.510279
H	-0.093883	-0.195586	0.146507
C	2.098239	1.028264	0.891436
C	2.199298	1.168015	-0.487296
C	2.244408	0.012574	-1.272934
C	2.046554	-0.230057	1.494175
C	1.899161	-0.347707	2.987289
C	2.084541	-1.359644	0.684599
C	2.175244	-1.256082	-0.704945
H	0.884138	-0.085551	3.290948
H	2.580145	0.329481	3.501134
H	2.099953	-1.362068	3.325152
H	2.052704	1.915326	1.512342
C	2.281906	2.528020	-1.126287
H	2.350320	0.108371	-2.349377
H	2.031326	-2.342137	1.138617
C	2.158046	-2.492554	-1.563047
H	1.677846	2.573291	-2.031856

H	3.311839	2.759044	-1.401758
H	1.930130	3.300855	-0.446504
H	2.769518	-3.280350	-1.125730
H	2.529322	-2.283802	-2.564534
H	1.140288	-2.876620	-1.653051

(3) HFIP-PMB

Name: HFIP-PMB
Charge: 0
Multiplicity: 1
E(UM062X) = -789.824102754 Ha

C	1.251389	-0.008837	-0.436304
C	2.041995	1.197642	0.049032
C	1.740660	-1.325006	0.157101
O	1.329143	-0.058034	-1.825294
H	0.429434	-0.086543	-2.165750
F	3.294490	1.201947	-0.391097
F	2.069294	1.256360	1.383849
F	1.448965	2.313039	-0.389966
F	3.035088	-1.540677	-0.054085
F	1.522088	-1.369452	1.475784
F	1.062278	-2.336890	-0.392733
H	0.234705	0.127696	-0.075011
C	-1.852706	1.256990	0.531182
C	-1.888764	-0.010206	1.135131
C	-1.884586	-1.178445	0.358233
C	-1.878682	1.356901	-0.865499
C	-1.853986	2.689809	-1.569286
C	-1.929376	0.187154	-1.619239
C	-1.917552	-1.078065	-1.038803
H	-0.910963	3.210399	-1.396624
H	-2.657170	3.339514	-1.221577
H	-1.970311	2.555161	-2.642510
C	-1.759080	2.496676	1.386737
C	-1.883353	-0.119711	2.640119
C	-1.828430	-2.529252	1.027798
H	-1.986978	0.266201	-2.699943
C	-1.945840	-2.299551	-1.922251
H	-1.013173	2.375089	2.171624
H	-2.711692	2.715131	1.874434
H	-1.479844	3.366982	0.801021
H	-1.554023	-3.310979	0.326254
H	-2.793291	-2.795160	1.465644
H	-1.092081	-2.536339	1.830548

H	-2.775838	-2.957424	-1.664345
H	-1.026931	-2.879747	-1.827442
H	-2.056908	-2.012306	-2.965943
H	-2.410981	-1.009097	2.974754
H	-2.356238	0.741605	3.104178
H	-0.860828	-0.181446	3.023576

(4) HFIP-Tol

Name: HFIP-Tol
Charge: 0
Multiplicity: 1
E(UM062X) = -789.824595284 Ha

C	0.872901	0.168622	-0.407348
C	1.812917	1.029259	0.425460
C	1.126187	-1.326495	-0.210296
O	1.047715	0.524295	-1.740816
H	0.187749	0.496684	-2.170279
F	3.061888	0.998578	-0.021746
F	1.816272	0.630781	1.701226
F	1.397508	2.296878	0.395785
F	2.413043	-1.648660	-0.304045
F	0.683982	-1.733047	0.984313
F	0.465025	-2.019261	-1.140747
H	-0.134637	0.369218	-0.042452
C	-2.406769	-0.994842	-0.245786
C	-2.313562	-0.442003	-1.517851
C	-2.278381	0.939031	-1.677967
C	-2.470111	-0.184230	0.885962
C	-2.522294	-0.788884	2.262527
C	-2.434852	1.199987	0.711534
C	-2.333990	1.759627	-0.555783
H	-1.513130	-0.991680	2.625364
H	-3.066075	-1.731758	2.255993
H	-3.003332	-0.115939	2.969711
H	-2.422419	-2.070900	-0.129597
H	-2.272265	-1.091429	-2.381972
H	-2.225073	1.374382	-2.667152
H	-2.480132	1.844870	1.579760
H	-2.304142	2.834337	-0.668631

(5) HFIP-Xyl

Name: HFIP-Xyl
Charge: 0
Multiplicity: 1
E(UM062X) = -789.818707413 Ha

C	-0.886977	-0.403426	-0.256647
C	-1.751077	-0.917646	0.875297
C	-1.535999	0.816525	-0.845589
O	-0.636121	-1.382405	-1.203894
H	0.262205	-1.286907	-1.587835
F	-2.868802	-1.495126	0.431392
F	-2.076559	0.079688	1.685895
F	-1.048586	-1.790065	1.620463
F	-2.803050	0.608470	-1.171124
F	-1.456391	1.861236	0.022899
F	-0.951411	1.201710	-1.964484
H	0.019445	-0.054383	0.187705
C	1.994012	1.243879	-0.693079
C	2.276532	-0.030770	-1.209493
C	2.558040	-1.154231	-0.383923
C	1.978901	1.504157	0.596066
C	1.645550	2.884653	1.060953
C	2.258821	0.488223	1.475175
C	2.521809	-0.829070	1.019438
H	0.759350	2.864369	1.691811
H	1.454521	3.545397	0.220078
H	2.461273	3.293993	1.652625
H	1.827997	2.076327	-1.362653
H	2.326011	-0.160373	-2.279510
C	2.817276	-2.566789	-0.916647
H	2.248794	0.699286	2.536399
H	2.698770	-1.605851	1.741041
H	3.810529	-2.904694	-0.632170
H	2.722816	-2.555542	-1.969043
H	2.079125	-3.258640	-0.521952

Cartesian coordinates of optimized geometries of HFIP/PhH conformers

(1) HFIP-PhH-1-SP

Name: HFIP-PhH-1-SP
Charge: 0
Multiplicity: 1
E(UM062X) = -789.824774823 Ha

C	-2.623215	1.230503	-0.235021
C	-2.556443	0.843275	1.098417
C	-2.543830	-0.506994	1.430240
C	-2.585740	-1.471330	0.429460
C	-2.656258	-1.085492	-0.904627
C	-2.683395	0.265891	-1.235943
H	-2.630293	2.279894	-0.493708
H	-2.509387	1.592352	1.876223
H	-2.490632	-0.807583	2.467263
H	-2.562010	-2.520725	0.687116
H	-2.694625	-1.835454	-1.682541
H	-2.764065	0.568291	-2.272565
F	2.722477	-1.198492	-0.541697
F	1.821925	-1.111549	1.416366
O	0.557092	-0.119188	-1.726543
H	-0.349372	0.088761	-1.969772
F	2.443466	1.527137	-0.285809
F	0.432494	2.271378	-0.489553
F	1.101016	1.487329	1.403068
F	0.955530	-2.353068	-0.119689
C	0.659324	-0.048878	-0.340303
H	-0.296275	-0.189551	0.166698
C	1.182322	1.322960	0.081061
C	1.565194	-1.187783	0.108290

(2) HFIP-PhH-2-SC

Name: HFIP-PhH-2-SC
Charge: 0
Multiplicity: 1
E(UM062X) = -789.827572997 Ha

C	2.687886	1.203204	0.585746
C	2.293212	1.201705	-0.747243
C	2.093093	-0.002741	-1.411672
C	2.289317	-1.206105	-0.744150
C	2.683781	-1.205153	0.588895
C	2.882592	-0.000441	1.254206
H	2.834400	2.140118	1.104722
H	2.128446	2.137297	-1.263370
H	1.768516	-0.003504	-2.442844
H	2.121318	-2.142556	-1.257653
H	2.826279	-2.141087	1.110784
H	3.181943	0.000400	2.292918
F	-2.549610	-1.391035	-0.043018

F	-0.864584	-1.311295	-1.389883
O	-1.212098	0.000426	1.915452
H	-2.175733	0.002423	1.917084
F	-2.546636	1.395327	-0.042490
F	-0.685619	2.336877	0.495895
F	-0.861909	1.312400	-1.389520
F	-0.690536	-2.336543	0.495291
C	-0.756007	0.000193	0.599785
H	0.332405	-0.001247	0.613828
C	-1.213122	1.273924	-0.107229
C	-1.215875	-1.272391	-0.107596

(3) HFIP-PhH-3-AP

Name: HFIP-PhH-3-AP
Charge: 0
Multiplicity: 1
E(UM062X) = -789.826954850 Ha

C	2.011716	-0.118137	1.418917
C	2.326912	1.095050	0.815712
C	3.032922	1.113001	-0.380832
C	3.423282	-0.080674	-0.975993
C	3.109872	-1.293507	-0.374112
C	2.404027	-1.313064	0.824254
H	1.447200	-0.133269	2.340722
H	2.007444	2.022041	1.270804
H	3.266672	2.055481	-0.855478
H	3.962139	-0.066359	-1.912805
H	3.405542	-2.222071	-0.841760
H	2.149174	-2.255748	1.288011
F	-0.858929	-1.524683	0.879251
F	-2.900922	-0.904835	0.548293
O	0.045623	-0.227436	-1.340127
H	0.662968	-0.349312	-0.603676
F	-0.800357	1.203153	1.154847
F	-0.648537	2.289815	-0.698211
F	-2.583880	1.724802	0.058528
F	-1.926285	-2.217401	-0.856586
C	-1.230659	0.021398	-0.867770
H	-1.892859	0.136504	-1.726000
C	-1.317985	1.327035	-0.072173
C	-1.743932	-1.168675	-0.056986

5. Single crystal x-ray diffraction of HFIPBz

Crystal Structure of $C_{10}H_6F_6O_2$

The low temperature [173(2)°K] single-crystal X-ray experiments were performed on a SuperNova diffractometer with Cu K_α radiation. Unit cell was obtained and refined by 1138 reflections with 5.3° < θ < 64.1°. No decay was observed in data collection. Raw intensities were corrected for Lorentz and polarization effects, and for absorption by empirical method. Direct phase determination yielded the positions of all non-hydrogen atoms. All non-hydrogen atoms were subjected to anisotropic refinement. The hydrogen atoms were generated geometrically with C-H bonds of 0.93-0.98 Å according to criteria described in the SHELXTL manual (Bruker, 1997). They were included in the refinement with $U_{iso}(H) = 1.2U_{eq}$ of their parent atoms. The final full-matric least-square refinement on F^2 converged with $R1 = 0.0740$ and $wR2 = 0.1202$ for 885 observed reflections [$I \geq 2\sigma(I)$]. The final difference electron density map shows no features. Details of crystal parameters, data collection and structure refinement are given in Table 1.

Data collection was controlled by CrysAlis^{Pro} (Rigaku, 2016). Computations were performed using the SHELXTL NT ver. 5.10 program package (Bruker, 1997) on an IBM PC 586 computer. Analytic expressions of atomic scattering factors were employed, and anomalous dispersion corrections were incorporated (*International Tables for X-ray Crystallography*, 1989). Crystal drawings were produced with XP (Bruker, 1997).

References

Bruker. (1997) SHELXTL. Structure Determination Programs, Version 5.10, Bruker AXS Inc.,6300 Enterprise Lane, Madison, WI 53719-1173, USA.

International Tables for X-ray Crystallography: (1989) Vol. C (Kluwer Academic Publishers, Dordrecht) Tables 4.2.6.8 and 6.1.1.4.

Rigaku. (2016) CrysAlis^{Pro}, Data Collection and Process Software for Rigaku Oxford Diffraction X-ray Diffractometer, Version 5.4, February, 2016. Rigaku Corporation, 9009, New Trails Drive, The Woodlands, TX77381, USA.

Table 1. Details of Data Collection, Processing and Structure Refinement

Sample code	**2021-1-26**		
Molecular formula	$C_{10}H_6F_6O_2$		
Molecular weight	272.15		
Color and habit	colorless block		
Crystal size	0.20 × 0.20 × 0.30 mm		
Crystal system	monoclinic		
Space group	*C*2/*m* (No. 12)		
Unit cell parameters	*a* = 18.4882(8) Å	α = 90.00°	
	b = 7.2780(3) Å	β = 117.625(4)°	
	c = 9.3093(4) Å	γ = 90.00°	
	V = 1109.83(9) $Å^3$	*Z* = 4	*F*(000) = 544
Density (calcd)	1.629 g/cm^3		
Diffractometer	SuperNova, Dual, Cu at zero, AtlasS2		
Radiation	Cu K_α, λ = 1.54184 Å		
Temperature	173(2)°K		
Scan type	ω-scan		
Data collection range	-21 < *h* < 21, -7 < *k* < 8, -10 < *l* < 10; θ_{max} = 64.8°		
Reflections measured	Total: 1794	Unique (*n*): 993	Observed [I ≥ 2σ(I)]: 885
Absorption coefficient	1.621 mm^{-1}		
Minimum and maximum transmission	0.536, 1.000		
No. of variables, *p*	97		
Weighting scheme	$w = \dfrac{1}{\sigma^2(F_o^2) + (0.001P)^2 + 4.5P}$	$P = (F_o^2 + 2F_c^2)/3$	
$R1 = \dfrac{\Sigma\|\|F_o\|-\|F_c\|\|}{\Sigma\|F_o\|}$ (for all reflections)	0.0794	0.0740 (for observed data)	
$wR2 = \sqrt{\dfrac{\Sigma[w(F_o^2 - F_c^2)^2]}{\Sigma w(F_o^2)^2}}$ (for all reflections)	0.1223	0.1202 (for observed data)	

Goof = S = $\sqrt{\frac{\Sigma[w(F_o^2 - F_c^2)^2]}{n - p}}$	1.185
Largest and mean Δ/σ	0.004, 0.001
Residual extrema in final difference map	-0.470 to 0.483 *e* Å^{-3}

Table 2. Atomic coordinates and equivalent isotropic temperature factors* (Å^2)

Atoms	x	y	z	$U_{eq.}$
F(1)	0.1699(2)	0.1856(6)	0.5016(5)	0.1555(19)
F(2)	0.0977(3)	0.3125(4)	0.2755(7)	0.184(2)
F(3)	0.04153(16)	0.1828(4)	0.4014(3)	0.0945(10)
O(1)	0.16892(17)	0.0000	0.2474(4)	0.0418(8)
O(2)	0.08676(19)	0.0000	-0.0221(4)	0.0668(12)
C(1)	0.2310(3)	0.0000	0.0761(5)	0.0346(10)
C(2)	0.2254(3)	0.0000	-0.0780(6)	0.0454(12)
C(3)	0.2957(3)	0.0000	-0.0947(6)	0.0525(13)
C(4)	0.3710(3)	0.0000	0.0405(7)	0.0538(14)
C(5)	0.3767(3)	0.0000	0.1937(7)	0.0518(13)
C(6)	0.3067(3)	0.0000	0.2121(6)	0.0419(11)
C(7)	0.1541(3)	0.0000	0.0885(5)	0.0393(11)
C(8)	0.0997(3)	0.0000	0.2755(6)	0.0410(11)
C(9)	0.1027(3)	0.1719(7)	0.3652(7)	0.0754(13)

*$U_{eq.}$ defined as one third of the trace of the orthogonalized **U** tensor.

Table 3. Bond lengths (Å) and bond angles (°)

F(1)-C(9)	1.305(6)	C(1)-C(7)	1.479(6)
F(2)-C(9)	1.296(6)	C(2)-C(3)	1.378(6)
F(3)-C(9)	1.324(4)	C(3)-C(4)	1.378(7)
O(1)-C(7)	1.373(5)	C(4)-C(5)	1.381(7)
O(1)-C(8)	1.419(5)	C(5)-C(6)	1.381(6)
O(2)-C(7)	1.192(5)	C(8)-C(9)$^{\#1}$	1.491(5)
C(1)-C(6)	1.386(6)	C(8)-C(9)	1.491(5)
C(1)-C(2)	1.390(6)		
C(7)-O(1)-C(8)	116.8(3)	O(1)-C(7)-C(1)	111.4(4)
C(6)-C(1)-C(2)	120.2(4)	O(1)-C(8)-C(9)$^{\#1}$	107.5(3)
C(6)-C(1)-C(7)	122.0(4)	O(1)-C(8)-C(9)	107.5(3)
C(2)-C(1)-C(7)	117.8(4)	C(9)$^{\#1}$-C(8)-C(9)	114.1(5)
C(3)-C(2)-C(1)	119.6(5)	F(2)-C(9)-F(1)	108.5(5)
C(4)-C(3)-C(2)	120.3(5)	F(2)-C(9)-F(3)	107.6(4)
C(3)-C(4)-C(5)	120.2(4)	F(1)-C(9)-F(3)	106.7(4)
C(6)-C(5)-C(4)	120.1(5)	F(2)-C(9)-C(8)	109.2(4)
C(5)-C(6)-C(1)	119.6(4)	F(1)-C(9)-C(8)	112.3(4)
O(2)-C(7)-O(1)	122.5(4)	F(3)-C(9)-C(8)	112.3(4)
O(2)-C(7)-C(1)	126.1(4)		

Symmtry transformation code: #1 (x, $-y$, z).

Table 4. Anisotropic thermal parameters* (Å^2)

Atoms	U_{11}	U_{22}	U_{33}	U_{23}	U_{13}	U_{12}
F(1)	0.110(2)	0.219(5)	0.159(3)	-0.141(3)	0.081(2)	-0.076(3)
F(2)	0.318(6)	0.0572(19)	0.339(6)	0.039(3)	0.290(6)	0.030(3)
F(3)	0.110(2)	0.0853(19)	0.142(2)	-0.0098(18)	0.103(2)	0.0109(17)
O(1)	0.0309(16)	0.060(2)	0.0386(17)	0.000	0.0198(14)	0.000
O(2)	0.0322(18)	0.129(4)	0.0377(19)	0.000	0.0153(16)	0.000
C(1)	0.032(2)	0.034(2)	0.042(2)	0.000	0.021(2)	0.000
C(2)	0.041(3)	0.053(3)	0.046(3)	0.000	0.023(2)	0.000
C(3)	0.056(3)	0.062(3)	0.056(3)	0.000	0.039(3)	0.000
C(4)	0.044(3)	0.058(3)	0.075(4)	0.000	0.042(3)	0.000
C(5)	0.033(3)	0.061(3)	0.063(3)	0.000	0.023(2)	0.000
C(6)	0.037(2)	0.047(3)	0.043(3)	0.000	0.020(2)	0.000
C(7)	0.034(2)	0.049(3)	0.037(2)	0.000	0.018(2)	0.000
C(8)	0.033(2)	0.055(3)	0.042(3)	0.000	0.023(2)	0.000
C(9)	0.089(3)	0.067(3)	0.111(4)	-0.010(3)	0.080(3)	-0.004(3)

*The exponent takes the form: $-2\pi^2\Sigma\Sigma U_{ij}h_ih_j\mathbf{a}_i^*\mathbf{a}_j^*$

Table 5. Coordinates and isotropic temperature factors* (Å^2) for H atoms

Atoms	x	y	z	$U_{eq.}$
H(2)	0.1745	0.0000	-0.1693	0.055
H(3)	0.2922	0.0000	-0.1976	0.063
H(4)	0.4182	0.0000	0.0284	0.065
H(5)	0.4277	0.0000	0.2846	0.062
H(6)	0.3104	0.0000	0.3152	0.050
H(8)	0.0500	0.0000	0.1711	0.049

*The exponent takes the form: $-8\pi^2 U \sin^2\theta/\lambda^2$

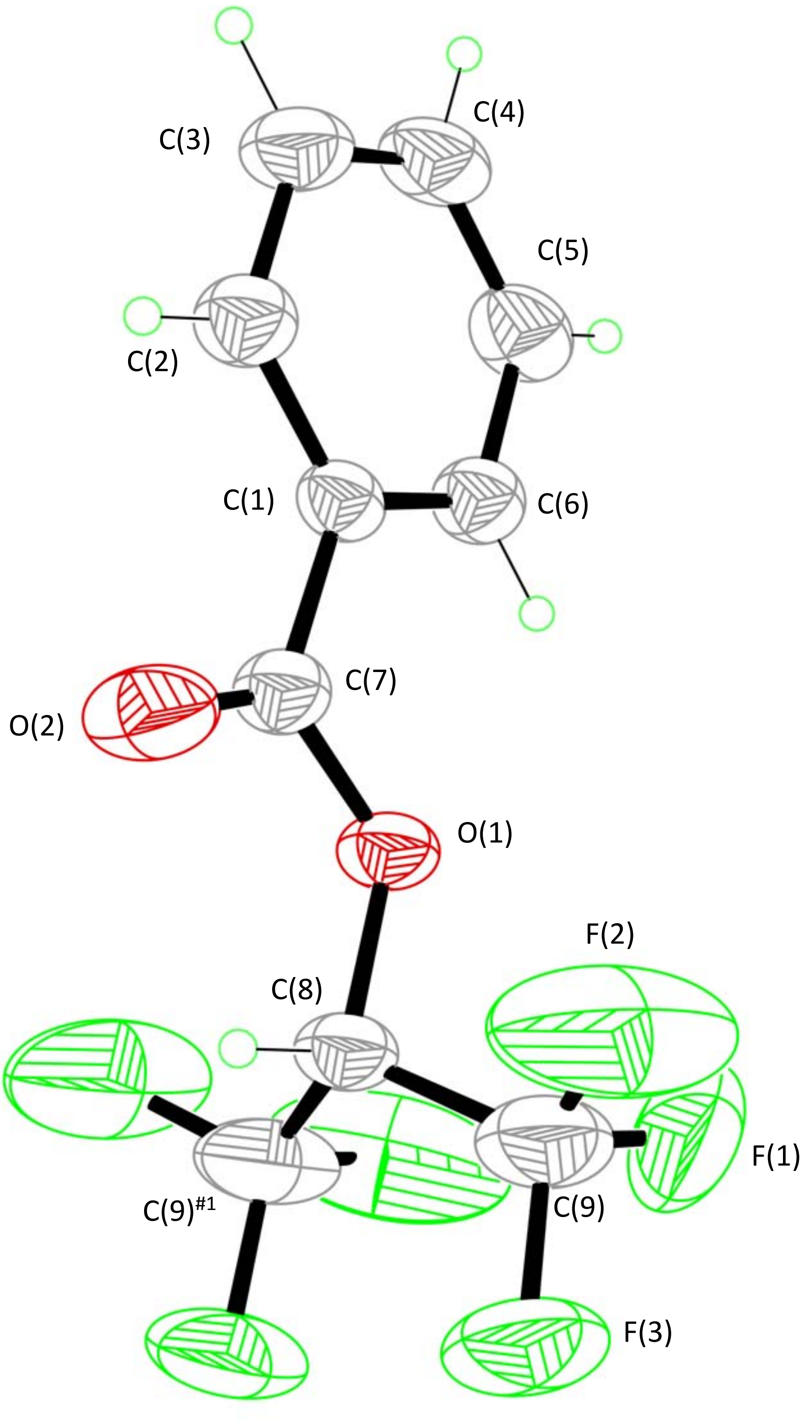

ORTEP drawing of $C_{10}H_6F_6O_2$ with 50% probability ellipsoids, showing the atomic numbering scheme.

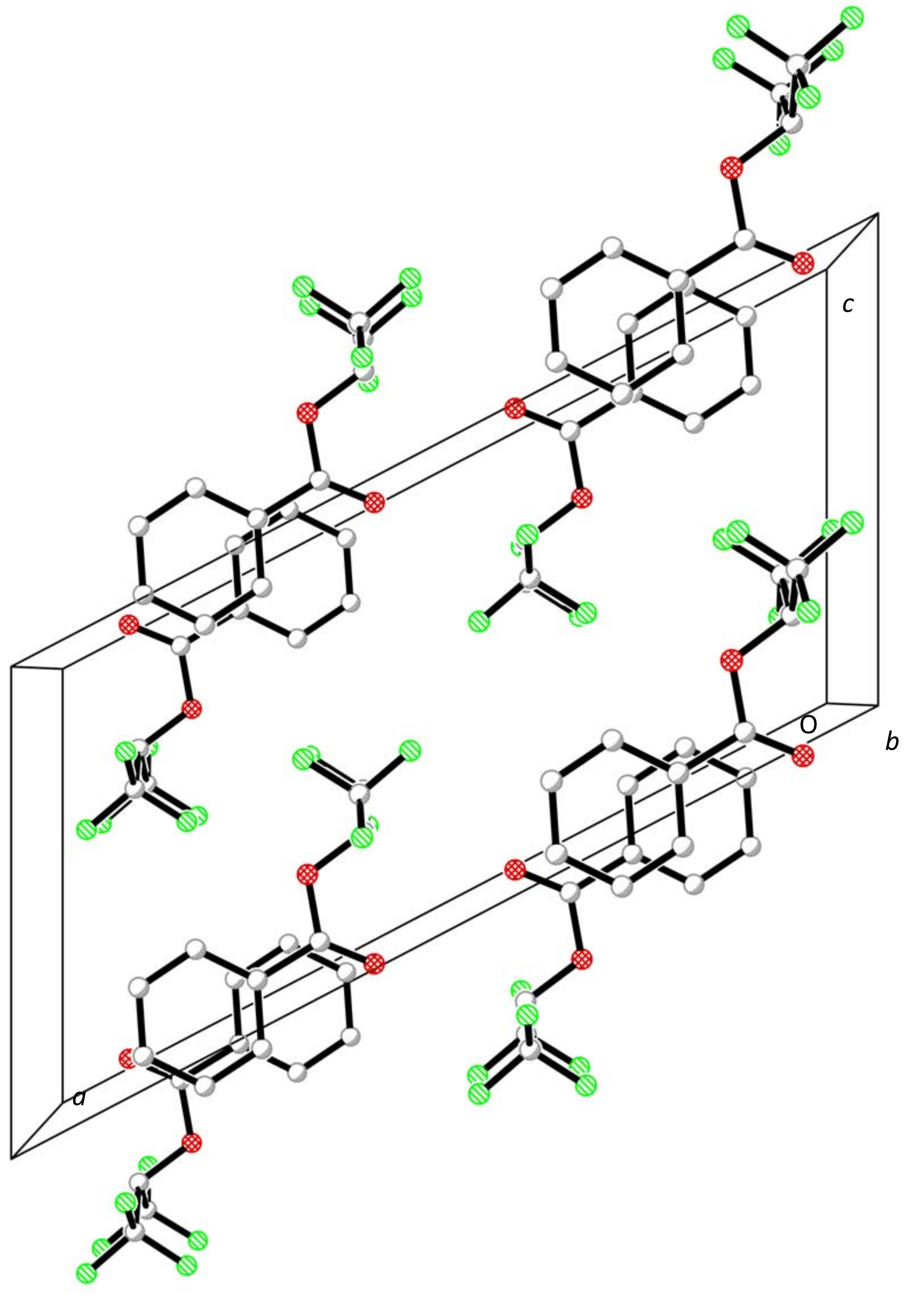

A packing view along the *b* direction
